# Supplementary material for: Aetiology of vaginal discharge, urethral discharge, and genital ulcer in sub-Saharan Africa: A systematic review and meta-regression
Source: PLoS Med. 2024 May 20;21(5):e1004385. doi: 10.1371/journal.pmed.1004385 (PMC11104670; doi:10.1371/journal.pmed.1004385)
Supplement: S1 Appendix — (PDF) [file pmed.1004385.s001.pdf]

Aetiology of vaginal discharge, urethral discharge, and genital ulcer in sub-Saharan Africa: A systematic review and meta-regression

Supplementary material

Julia Michalow[1*], Magdalene K Walters[1†], Olanrewaju Edun[1†], Max Wybrant[1], Bethan Davies[2], Tendesayi Kufa[3], Thabitha Mathega[3], Sungai T Chabata[4], Frances M Cowan[4,5], Anne Cori[1], Marie-Claude Boily[1], Jeffrey W Imai-Eaton[1,6]

[1] MRC Centre for Global Infectious Disease Analysis, School of Public Health, Imperial College London, London, United Kingdom

[2] MRC Centre for Environment and Health, Department of Epidemiology and Biostatistics, School of Public Health, Imperial College London, London, United Kingdom

[3] Centre for HIV & STI, National Institute for Communicable Diseases, Johannesburg, South Africa

[4] Centre for Sexual Health and HIV AIDS Research (CeSHHAR), Harare, Zimbabwe

[5] Department of International Public Health, Liverpool School of Tropical Medicine, Liverpool, United Kingdom

[6] Center for Communicable Disease Dynamics, Department of Epidemiology, Harvard T.H. Chan School of Public Health, Boston, MA, USA

* Correspondence to:

Julia Michalow
MRC Centre for Global Infectious Disease Analysis
School of Public Health
Imperial College London
London
United Kingdom
j.michalow21@imperial.ac.uk

[†] Contributed equally

Table of Contents

Table A: Search terms for symptom, pathogen, and sub-Saharan Africa, by database. 3

Table B: Sub-Saharan African countries included in the analysis, classified by region. 5

Table C: Variables extracted from included reports. 5

Text A: Preparation and extraction of unpublished data..6

Table D: Surveys by National Institute for Communicable Diseases in South Africa. 6

Text B: Adjusting reported proportions to account for diagnostic test performance 7

Table E: Performance of diagnostic tests among women with vaginal discharge. 8

Table F: Performance of diagnostic tests among men with urethral discharge. 9

Table G: Performance of diagnostic tests among adults with genital ulcer. 10

Table H: Study critical appraisal tool. 11

Fig A: Number of studies included per symptom by country in sub-Saharan Africa. 12

Table I: Summary of participant and study characteristics for studies included in the analysis of regional trends in aetiology, among adults of mixed or unmeasured HIV status. 13

Table J: Summary of participant and study characteristics for studies included in the analysis to assess the effect of HIV status, among adults of known HIV status. 14

Table K: Summary of participant and study characteristics for studies included in the analysis to assess the effect of age, among youth < 25 years and adults ≥ 25 years. 15

Table L: Generalized linear mixed-effects models on the odds of vaginal discharge, urethral discharge, and genital ulcer among adults of mixed or unmeasured HIV status in sub-Saharan Africa. 16

Table M: Generalized linear mixed-effects models on the odds of vaginal discharge, urethral discharge, and genital ulcer among adults of known HIV status in sub-Saharan Africa. 17

Table N: Generalized linear mixed-effects models on the odds of vaginal discharge, urethral discharge, and genital ulcer among youth under 25 years and adults 25 years and older in sub-Saharan Africa. 18

Fig B: Estimated diagnosed proportion per pathogen among symptomatic adults of mixed HIV status in sub-Saharan Africa, by region over time. 19

Table O: Critical appraisal of reports included in the review. 20

Table P: Summary of critical appraisal criteria for included reports. 26

Fig C: Effect of critical appraisal criteria on the odds of diagnosis per pathogen, among symptomatic adults of mixed or unmeasured HIV status in sub-Saharan Africa. 28

Table Q: Estimated diagnosed proportion per pathogen in sub-Saharan Africa, with and without diagnostic test performance adjustment. 29

Fig D: Estimated diagnosed proportion per pathogen among symptomatic adults of mixed or unmeasured HIV status in sub-Saharan Africa over time, with and without diagnostic test performance adjustment. 30

Fig E: Estimated diagnosed proportion per pathogen among symptomatic adults of mixed or unmeasured HIV status in sub-Saharan Africa over time, using NAAT with and without diagnostic test performance adjustment. 31

Table A: Search terms for symptom, pathogen, and sub-Saharan Africa, by database.

**MEDLINE search strategy**<br>Search conducted 20 December 2023 with 1881 articles retrieved.
**Symptoms domain:**<br>exp ulcer/ or exp vaginal discharge/ or exp urethritis/ or exp uterine cervicitis/ OR<br>(GUD or ((genital* or anogenital or penile or penis or vagin*) adj2 (ulcer* or vesicle* or papule* or chancr* or granulom* or sore* or lesion*)) or ((genital* or penile or penis or urethra* or vagin* or cervic* or cervix) adj2 discharge) or urethritis or cervicitis or vaginitis).ab,kw,ti
**AND RTI pathogen domain:**<br>exp Sexually Transmitted Diseases/ or exp Chancroid/ or exp Haemophilus ducreyi/ or exp Chlamydia infections/ or exp Chlamydia trachomatis/ or exp Lymphogranuloma Venereum/ or exp Gonorrhea/ or exp Neisseria gonorrhoeae/ or exp Syphilis/ or exp Treponema pallidum/ or exp Herpes Simplex/ or exp Herpesvirus 1, Human/ or exp Herpesvirus 2, Human/ or exp HIV infections/ or exp Trichomonas Infections/ or exp Trichomonas vaginalis/ or exp Mycoplasma genitalium/ or exp Vaginosis, Bacterial/ or exp Candidiasis, Vulvovaginal/ or exp Candida albicans/ OR<br>(sexually transmitted disease* or sexually transmitted infection* or STI* or STD* or chancroid* or ducreyi or chlamydia* or Lymphogranuloma Venereum or LGV or gonorrhoea* or gonorrhoeae* or gonorrhea* or syphili* or treponema palli* or herpe* or herpes simplex* or HSV* or HSV type* or HIV* or human immunodeficiency virus or trichomonas* or trichomoniasis* or vaginalis* or mycoplasma or genitalium* or vaginosis* or bacterial vaginosis* or candida* or candidiasis* or albicans).ab,kw,ti
**AND Sub-Saharan Africa domain:**<br>exp Africa south of the Sahara/ OR<br>(Africa* or Afriq* or Angola*or Benin* or Botswana* or Motswana* or Batswana* or Burkina* or Burundi* or Cabo Verde* or Cap-Vert* or Cape verde* or Camero* or Central African Republic* or Republique centrafricaine or Chad* or Comor* or Congo* or Democratic Republic of Congo or Republique democratique du Congo DRC or RDC or Cote d'Ivoire or Ivory Coast or Ivorian* or Guinea* or Eritrea* or Erythree*or Ethiop* or Gabon* or Gambi* or Ghana* or Guine* or Equatorial Guinea* or Guinee Equatoriale or Equatoguinean* or Guinea-Bissau* or Kenya* or Lesotho* or Basotho* or Liberia* or Madagas* or Malawi* or Mali* or Mauritani* or Mauri* or Mozambi* or Namibi* or Niger* or Nigeria* or Rwanda* or Rouanda* or Ruanda* or Sao Tome* or Senegal* or Seychel* or Sierra Leone* or Somali* or South Africa* or Afrique du Sud or South Sudan or Soudan de sud or Sudan* or Swazi* or eSwatini* or Tanzani* or Togo* or Uganda* or Ouganda* or Zambi* or Zimbabwe*).mp
**Embase search strategy**<br>Search conducted 20 December 2023 with 2835 articles retrieved.
**Symptoms domain:**<br>exp genital ulcer/ or exp vagina discharge/ or exp urethritis/ or exp urethral discharge/ or exp uterine cervicitis/ OR<br>(GUD or ((genital* or anogenital or penile or penis or vagin*) adj2 (ulcer* or vesicle* or papule* or chancr* or granulom* or sore* or lesion*)) or ((genital* or penile or penis or urethra* or vagin* or cervic* or cervix) adj2 discharge) or urethritis or cervicitis or vaginitis).ab,kw,ti
**AND RTI pathogen domain:**<br>exp sexually transmitted disease/ or exp ulcus molle/ or exp Haemophilus ducreyi/ or exp Chlamydia/ or exp Chlamydia trachomatis/ or exp lymphogranuloma venereum/ or exp gonorrhea/ or exp Neisseria gonorrhoeae/ or exp syphilis/ or exp Treponema pallidum/ or<br>exp Herpes simplex virus/ or exp Herpes simplex virus 1/ or exp Herpes simplex virus 2/ or exp Human immunodeficiency virus/ or exp Trichomonas/ or Trichomonas vaginalis/ or exp trichomoniasis/ or exp Mycoplasma genitalium/ or exp vaginitis/ or exp vagina candidiasis/ or Candida albicans/ OR<br>(sexually transmitted disease* or sexually transmitted infection* or STI* or STD* or chancroid* or ducreyi or chlamydia* or Lymphogranuloma Venereum or LGV or gonorrhoea* or gonorrhoeae* or gonorrhea* or chancre* or syphili* or treponema palli* or herpe* or herpes simplex* or HSV* or HSV type* or HIV* or human immunodeficiency virus or trichomonas* or trichomoniasis* or vaginalis* or mycoplasma or genitalium* or vaginosis* or bacterial vaginosis* or candida* or candidiasis* or albicans).ab,kw,ti
**AND Sub-Saharan Africa domain**<br>exp Africa south of the Sahara/ or exp africa, eastern/ or exp africa, western/ or exp africa, central/ or exp africa, southern/ OR<br>(Africa* or Afriq* or Angola*or Benin* or Botswana* or Motswana* or Batswana* or Burkina* or Burundi* or Cabo Verde* or Cap-Vert* or Cape verde* or Camero* or Central African Republic* or Republique centrafricaine or Chad* or Comor* or Congo* or Democratic Republic of Congo or Republique democratique du Congo DRC or RDC or Cote d'Ivoire or Ivory Coast or Ivorian* or Guinea* or Eritrea* or Erythree*or Ethiop* or Gabon* or Gambi* or Ghana* or Guine* or Equatorial Guinea* or Guinee Equatoriale or Equatoguinean* or Guinea-Bissau* or Kenya* or Lesotho* or Basotho* or Liberia* or Madagas* or Malawi* or Mali* or Mauritani* or Mauri* or Mozambi* or Namibi* or Niger* or Nigeria* or Rwanda* or Rouanda* or Ruanda* or Sao Tome* or Senegal* or Seychel* or Sierra Leone* or Somali* or South Africa* or Afrique du Sud or South Sudan or Soudan de sud or Sudan* or Swazi* or eSwatini* or Tanzani* or Togo* or Uganda* or Ouganda* or Zambi* or Zimbabwe*).mp
**Global Health search strategy**<br>Search conducted 20 December 2023 with 1566 articles retrieved.
**Symptoms domain:**<br>exp genital ulcers/ or urethritis/ or exp cervicitis/ OR<br>(GUD or ((genital* or anogenital or penile or penis or vagin*) adj2 (ulcer* or vesicle* or papule* or chancr* or granulom* or sore* or lesion*)) or ((genital* or penile or penis or urethra* or vagin* or cervic* or cervix) adj2 discharge) or urethritis or cervicitis or vaginitis).ab,ti,mp.
**AND RTI pathogen domain:**

exp sexually transmitted disease/ or exp chancroid/ or exp Haemophilus ducreyi/ or exp Chlamydia/ or exp Chlamydia trachomatis/ or exp gonorrhoea/ or exp Neisseria gonorrhoeae/ or exp syphilis/ or exp Treponema pallidum/ or exp herpes simplex virus/ or exp Human herpesvirus 1/ or exp Human herpesvirus 2/ or exp human immunodeficiency virus/ or exp Trichomonas/ or Trichomonas vaginalis/ or exp trichomoniasis/ or exp Mycoplasma genitalium/ or exp bacterial vaginitis/ or exp Candida albicans/ OR<br>(sexually transmitted disease* or sexually transmitted infection* or STI* or STD* or chancroid* or ducreyi or chlamydia* or Lymphogranuloma Venereum or LGV or gonorrhoea* or gonorrhoeae* or gonorrhea* or chancre* or syphili* or treponema palli* or herpe* or herpes simplex* or HSV* or HSV type* or HIV* or human immunodeficiency virus or trichomonas* or trichomoniasis* or vaginalis* or mycoplasma or genitalium* or vaginosis* or bacterial vaginosis* or candida* or candidiasis* or albicans).ab,ti,mp.
**AND Sub-Saharan Africa domain**<br>exp Africa south of the Sahara/ or exp Central Africa/ or exp East Africa/ or exp Southern Africa/ or exp West Africa/ OR (Africa* or Afriq* or Angola*or Benin* or Botswana* or Motswana* or Batswana* or Burkina* or Burundi* or Cabo Verde* or Cap-Vert* or Cape verde* or Camero* or Central African Republic* or Republique centrafricaine or Chad* or Comor* or Congo* or Democratic Republic of Congo or Republique democratique du Congo DRC or RDC or Cote d'Ivoire or Ivory Coast or Ivorian* or Guinea* or Eritrea* or Erythree*or Ethiop* or Gabon* or Gambi* or Ghana* or Guine* or Equatorial Guinea* or Guinee Equatoriale or Equatoguinean* or Guinea-Bissau* or Kenya* or Lesotho* or Basotho* or Liberia* or Madagas* or Malawi* or Mali* or Mauritani* or Mauri* or Mozambi* or Namibi* or Niger* or Nigeria* or Rwanda* or Rouanda* or Ruanda* or Sao Tome* or Senegal* or Seychel* or Sierra Leone* or Somali* or South Africa* or Afrique du Sud or South Sudan or Soudan de sud or Sudan* or Swazi* or eSwatini* or Tanzani* or Togo* or Uganda* or Ouganda* or Zambi* or Zimbabwe*).mp
**Web of Science search strategy**<br>Search conducted 20 December 2023 with 1498 articles retrieved.
**Symptoms domain:**<br>TS=(((genital* or anogenital or penile or penis or vagin*) near/2 (ulcer* or vesicle* or papule* or chancr* or granulom* or sore* or lesion*)) or ((genital* or penile or penis or urethra* or vagin* or cervic* or cervix) near/2 discharge) or GUD or urethritis or cervicitis or vaginitis)
**AND RTI pathogen domain:**<br>TS=("sexually transmitted disease*" or "sexually transmitted infection*" or STI* or STD* or chancroid* or ducreyi or chlamydia* or "Lymphogranuloma Venereum" or LGV or gonorrhoea* or gonorrhoeae* or gonorrhea* or chancre* or syphili* or "treponema palli*" or herpe* or "herpes simplex*" or HSV* or HSV type* or HIV* or "human immunodeficiency virus" or trichomonas* or trichomoniasis* or vaginalis* or mycoplasma or genitalium* or vaginosis* or bacterial vaginosis* or candida* or candidiasis* or albicans)
**AND Sub-Saharan Africa domain**<br>TS=(Africa* or Afriq* or Angola*or Benin* or Botswana* or Motswana* or Batswana* or Burkina* or Burundi* or Cabo Verde* or Cap-Vert* or Cape verde* or Camero* or Central African Republic* or Republique centrafricaine or Chad* or Comor* or Congo* or Democratic Republic of Congo or Republique democratique du Congo DRC or RDC or Cote d'Ivoire or Ivory Coast or Ivorian* or Guinea* or Eritrea* or Erythree*or Ethiop* or Gabon* or Gambi* or Ghana* or Guine* or Equatorial Guinea* or Guinee Equatoriale or Equatoguinean* or Guinea-Bissau* or Kenya* or Lesotho* or Basotho* or Liberia* or Madagas* or Malawi* or Mali* or Mauritani* or Mauri* or Mozambi* or Namibi* or Niger* or Nigeria* or Rwanda* or Rouanda* or Ruanda* or Sao Tome* or Senegal* or Seychel* or Sierra Leone* or Somali* or South Africa* or Afrique du Sud or South Sudan or Soudan de sud or Sudan* or Swazi* or eSwatini* or Tanzani* or Togo* or Uganda* or Ouganda* or Zambi* or Zimbabwe*)

Table B: Sub-Saharan African countries included in the analysis, classified by region.

Region	Countries
Eastern Africa	Burundi, Comoros, Djibouti, Eritrea, Ethiopia, Kenya, Madagascar, Malawi, Mauritius, Mozambique, Rwanda, Seychelles, Somalia, South Sudan, Uganda, United Republic of Tanzania, Zambia, Zimbabwe
Southern Africa	Botswana, Eswatini, Lesotho, Namibia, South Africa
Western Africa	Benin, Burkina Faso, Cabo Verde, Cote d'Ivoire, Gambia, Ghana, Guinea-Bissau, Guinea, Liberia, Mali, Mauritania, Niger, Nigeria, Senegal, Sierra Leone, Togo
Central Africa	Angola, Cameroon, Central African Republic, Chad, Congo, Democratic Republic of Congo, Equatorial Guinea, Gabon, Sao Tome and Principe

Countries classified according to UN M49 Standard.[1]

Table C: Variables extracted from included reports.

Category	Extracted variables
Study characteristics	• Authors<br>• Publication title<br>• Publication year<br>• Years and duration of data collection<br>• Countries of study<br>• Subnational regions or cities of study<br>• Study site names<br>• Study site types<br>• Study design<br>• Study sampling method
Participant characteristics	• Study population<br>• Study population age (mean, median, range)<br>• Study population HIV status<br>• Study population HIV prevalence
Diagnostic methods	• Diagnostic test and specimen for each infection<br>• Number tested for each infection<br>• Number positive for each infection<br>• Proportion diagnosed positive with each infection

Text A: Preparation and extraction of unpublished data

The literature search identified two databases. The first, from the South Africa National Institute for Communicable Diseases (NICD), covered surveillance among primary healthcare clinic attendees in South Africa from 2006 to 2022. These data were reported in several publications,[2–14] yet the database included unpublished surveillance rounds and enabled additional outcome stratification. The second was the Centre for Sexual Health and HIV/AIDS Research (CeSHHAR) female sex worker population size estimation study in Zimbabwe in 2017. Conference abstracts reporting study results prompted request for special tabulation. Data extracted from each database, following approaches outlined below, were combined with published study data prior to analysis.

South Africa NICD data extraction

Data were available from 13 routine aetiologic surveillance rounds, which were cross-sectional aetiologic prevalence surveys among clinic attendees examined and diagnosed with vaginal discharge, urethral discharge, and genital ulcer syndromes (Table E). Variables were standardised and combined into a single database. We extracted simple (unweighted) diagnosed proportions, stratified by year, sex, HIV status, and age group. We calculated HIV-prevalence and mean age for each stratum. Prior full-text publications of these data were excluded from our review.

Table D: Surveys by National Institute for Communicable Diseases in South Africa.

Year(s)	Province	Facilities	Number participants
2006-2007	Gauteng<br>Northern Cape	Alexandra Health Centre<br>Betty Gaetsewe Clinic, City Clinic, Masakhane Clinic	529 (4.0%)<br>416 (3.2%)
2006-2007	Western Cape	Spencer Road Clinic	243 (1.9%)
2007-2008	Free State	Central Park Clinic, Pelonomi Poly Clinic	360 (2.7%)
2007-2009	Gauteng	Alexandra Health Centre	992 (7.5%)
2010	Gauteng	Alexandra Health Centre	1 057 (8.0%)
2010-2012	Gauteng<br>Limpopo	Alexandra Health Centre<br>Rethabile Heath Centre	720 (5.5%)<br>402 (3.0%)
2011-2012	Gauteng	Alexandra Health Centre	722 (5.5%)
2013-2014	Gauteng	Alexandra Health Centre	546 (4.0%)
2013-2014	KwaZulu-Natal<br>Mpumalanga	Central Durban Clinic<br>Nelspruit Clinic	406 (3.1%)<br>243 (1.9%)
2014-2016	Eastern Cape<br>Gauteng<br>KwaZulu-Natal<br>Mpumalanga<br>North West	Gqebera Clinic<br>Alexandra Health Clinic<br>East Boom Clinic, Phoenix Clinic<br>Hluvukani Clinic, Kabokweni Clinic<br>Jouberton Clinic	92 (0.7%)<br>735 (5.6%)<br>438 (3.3%)<br>436 (3.3%)<br>227 (1.7%)
2017	Eastern Cape<br>Free State<br>Gauteng<br>Western Cape	Zwide Clinic<br>Heidedal Clinic<br>Alexandra Clinic<br>Khayelitsha Site B Clinic, Khayelitsha Site C Clinic	259 (1.9%)<br>129 (1.0%)<br>373 (2.8%)<br>223 (1.7%)
2018-2019	Gauteng<br>Limpopo<br>Northern Cape	Alexandra Clinic<br>Rethabile Health Centre<br>Kimberly Central Clinic	356 (2.7%)<br>139 (1.1%)<br>209 (1.6%)
2020-2022	Gauteng<br>KwaZulu-Natal<br>Western Cape	Alexandra Health Centre<br>Prince Cyril Zulu Communicable Disease Centre<br>Spencer Road Clinic	855 (6.5%)<br>1 032 (7.8%)<br>399 (3.8%)
**Total**			13 159 (100%)

CeSHHAR female sex worker survey data extraction

The female sex worker population size estimation study in Zimbabwe included 2507 women (1497 Harare, 808 Bulawayo, 202 Shamva) recruited using respondent-driven sampling (RDS).[15] A random sample of one third of participants were offered genital examination and STI testing, of whom 80 were identified as having abnormal vaginal discharge. Data were survey-weighted using RDS weights to determine diagnosed proportions and 95% CIs, with stratification by HIV status and age group. We calculated weighted HIV-prevalence and mean age for each stratum. We determined numerators and denominators for each proportion that accounted for the sampling design, using the method described by Stannah et al. (2023).[16] For each observation, we estimated a design effect using the ratio of variances of the RDS-adjusted proportion and simple random sample proportion, which was used to derive the effective sample size and number of individuals who tested positive. Ethical approval for the study was obtained from the Medical Research Council of Zimbabwe (MRCZ/A/1942) and London School of Hygiene & Tropical Medicine (#9686).

Text B: Adjusting reported proportions to account for diagnostic test performance

Diagnostic test performance

We classified diagnostic tests from included studies into broad categories. Antibody tests included enzyme immunoassays or enzyme-linked immunosorbent assays. Culture included any culture test with a valid medium. Nucleic acid amplification tests (NAAT) included polymerase chain reaction, strand displacement assay, transcription mediated assay, ligase chain reaction, and GeneXpert. Treponemal tests for syphilis included Treponema pallidum Particle Agglutination Assay, Treponema pallidum Hemagglutination Assay, Fluorescent Treponemal Antibody Absorption, Microhemagglutination Assay for Treponema pallidum, and point of care rapid tests. Non-treponemal tests for syphilis included the Venereal Disease Research Laboratory test and Rapid Plasma Reagin test. Syphilis tests were categorised regardless of titre due to variations in reporting.

In studies aimed at detecting candidiasis, extractions initially labelled *Candida albicans* according to study descriptions were reclassified as *Candida* species if the diagnostic tests (culture, wet mount, and gram stain) were not suitable for identifying *Candida albicans*. Studies that described detecting *Gardnerella vaginalis* with diagnostic tests (Amsel Criteria or Nugent Score) appropriate for detecting bacterial vaginosis were reclassified as such.

Point value sensitivity and specificity estimates for each diagnostic test category were compiled from literature, with priority given to performance characteristics published by the WHO (Tables S5 – S7).[17,18] In cases where multiple sources were available with wide variation, we calculated a weighted mean for the sensitivity and specificity. We made the following assumptions where data were lacking on performance characteristics. For Candida species, we assumed that the accuracy of gram stain was equivalent to wet mount, although the sensitivity of the former test is likely higher. For *Trichomonas vaginalis*, we assumed that the accuracy of culture using a urine sample was equivalent to that of culture with a genital fluid sample,[18,19] and that the accuracy of wet mount using a urine sample was equivalent to that of wet mount with a genital fluid sample.[18,20] In studies that reported using two diagnostic tests to identify a pathogen, we determined a combined sensitivity and specificity estimate: if either test in the study could be positive, we used the lower sensitivity and lower specificity of the two tests, and if both tests had to be positive, we used the lower sensitivity and higher specificity of the two tests.

Diagnostic test performance varies based on factors such as disease stage, sample handling, and performer competency. For example, WHO advises that darkfield microscopy is used in specialised laboratories by experienced microscopists.[21] However, we lacked data to evaluate such influencing factors.

Bayesian estimation

The Rogan-Gladen estimator (Eq. 1) is commonly used to estimate the true prevalence (p^{true}) of infection based on observed prevalence ($p^{observed}$) measured by an imperfect diagnostic, accounting for the sensitivity and specificity of a given diagnostic test.[22] However, direct application of the equation can result in negative values for p^{true} when $p^{observed}$ is lower than the probability of a false positive result (1 - $specificity$). We therefore used a Bayesian approach to estimate p^{true} in Eq. 1 by accounting for the observed sample size (N) and number of positive test results (n) (Eq 2.), and applying a uniform prior probability for p_{true} between 0 and 1 (Eq. 3).[23,24]

$$p^{observed} = p^{true} * \ sensitivity + (1 - p^{true}) * \ (1 - specificity) \quad \text{(Eq. 1)}$$

$$n \sim binomial(N, p^{observed}) \quad \text{(Eq. 2)}$$

$$p^{true} \sim uniform(0,1) \quad \text{(Eq. 3)}$$

The model was fit using Markov Chain Monte Carlo, specifically the NUTS (No-U-Turn Sampler) algorithm, with four chains that each ran for 2000 iterations. Convergence was assessed qualitatively using trace plots of model parameters and quantitatively using the Gelman-Rubin statistic (R-hat). The posterior mean estimate ($\widehat{p^{true}}$) and standard error (se^{true}) were used to calculate adjusted numerators ($n^{adjusted}$) and denominators ($N^{adjusted}$) for each observation (Eq. 4 and 5), which were then used for pooling in generalized linear mixed-effects models.

$$N^{adjusted} = \frac{\widehat{p^{true}}\left(1-\widehat{p^{true}}\right)}{(se^{true})^2} \quad \text{(Eq. 4)}$$

$$n^{adjusted} = p^{true} * \ N^{adjusted} \quad \text{(Eq. 5)}$$

Table E: Performance of diagnostic tests among women with vaginal discharge.

Pathogen	Test	Specimen	Number studies	Sensitivity (%)	Specificity (%)	Source
BV	Amsel criteria	Genital fluid	9	51.0	98.0	Dadhwal 2010[25]
	Nugent score	Genital fluid	16	87.0	95.0	Thomason 1992[26] Schwebke 1996[27]
	Amsel criteria and Nugent score	Genital fluid	2	51.0	98.0	Combined test estimate*
	Amsel criteria or Nugent score	Genital fluid	1	51.0	95.0	Combined test estimate*
CA	Germ tube	Genital fluid	7	98.9	98.5	Hoppe 1999[28]
	NAAT	Genital fluid	2	90.9	94.1	Gaydos 2017[29]
CS	Culture	Genital fluid	8	67.0	66.0	WHO 1999[17]
	Gram stain	Genital fluid	11	54.6	89.6	Assumed equivalent to wet mount, but sensitivity likely higher.
	Wet mount	Genital fluid	6	54.6	89.6	Chatwani 2007[30] Schwebke 2018[31]
	Gram stain and culture	Genital fluid	6	54.6	89.6	Combined test estimate*
	Wet mount and culture	Genital fluid	3	54.6	89.6	Combined test estimate*
CT	Culture	Genital fluid	1	63.4	100.0	WHO 2011[18]
	DFA	Genital fluid	5	82.0	98.5	WHO 1999[17]
	ELISA	Genital fluid	6	65.0	100.0	WHO 2011[18]
	NAAT	Genital fluid	18	88.6	99.6	WHO 2011[18]
	NAAT	Urine	2	87.0	99.8	WHO 2011[18]
	Rapid antigen test	Genital fluid	1	48.0	98.0	Grillo-Ardila 2020[32]
	ELISA and NAAT	Genital fluid	1	65.0	99.6	Combined test estimate*
	Culture and DFA	Genital fluid	1	97.0	99.8	Combined test estimate*
MG	NAAT	Genital fluid	7	93.3	97.6	Gaydos 2019[33]
NG	Culture	Genital fluid	26	75.7	100.0	WHO 2011[18]
	NAAT	Genital fluid	15	93.3	99.2	WHO 2011[18]
	NAAT	Urine	1	91.6	100.0	WHO 2011[18]
TV	Culture	Genital fluid	6	68.8	100.0	WHO 2011[18]
	NAAT	Genital fluid	17	95.0	98.0	WHO 2011[18]
	Wet mount	Genital fluid	33	52.0	100.0	WHO 2011[18]
	Culture and NAAT	Genital fluid	1	68.8	100.0	Combined test estimate*
	Culture or NAAT	Genital fluid	1	68.8	98.0	Combined test estimate*
	Culture and wet mount	Genital fluid	2	52.0	100.0	Combined test estimate*

Number of studies indicates the total count of studies included within the systematic review that used each diagnostic test and specimen combination for the detection of a specific pathogen.

* Combined sensitivity and specificity estimate determined for studies reporting two diagnostic tests to identify a pathogen. If either test could be positive, the lower sensitivity and lower specificity values were used from the two tests. If both tests had to be positive, the lower sensitivity and higher specificity values were used from the two tests.

Table F: Performance of diagnostic tests among men with urethral discharge.

Pathogen	Test	Specimen	Number studies	Sensitivity (%)	Specificity (%)	Source
CT	Culture	Genital fluid	1	63.4	100.0	WHO 2011[18]
	DFA	Genital fluid	1	82.0	98.5	WHO 2011[18]
	ELISA	Genital fluid	10	84.0	98.0	WHO 1999[17]
	ELISA	Urine	1	84.0	98.0	WHO 1999[17]
	NAAT	Genital fluid	9	87.5	99.2	WHO 2011[18]
	NAAT	Urine	7	87.8	99.3	WHO 2011[18]
	Culture and DFA	Genital fluid	2	63.4	100.0	Combined test estimate*
MG	NAAT	Genital fluid	4	98.1	99.9	Gaydos 2019[33]
	NAAT	Urine	4	89.4	99.1	Gaydos 2019[33]
NG	Culture	Genital fluid	30	87.6	100.0	WHO 2011[18]
	Gram stain	Genital fluid	3	87.6	100.0	WHO 2011[18]
	NAAT	Genital fluid	7	96.1	99.0	WHO 2011[18]
	NAAT	Urine	6	80.9	99.9	WHO 2011[18]
	Gram stain and culture	Genital fluid	2	87.6	100.0	Combined test estimate*
	Gram stain or NAAT	Genital fluid and urine	1	87.6	99.9	Combined test estimate*
	Culture or NAAT	Genital fluid	1	87.6	99.0	Combined test estimate*
TV	Culture	Genital fluid	4	74.5	100.0	WHO 2011[18]
	Culture	Urine	1	74.5	100.0	Performance of urine culture and urethral swab culture assumed equivalent, based on WHO 2011[18] and Kaydos-Daniels 2004[19]
	NAAT	Genital fluid	7	81.6	97.7	WHO 2011[18]
	NAAT	Urine	4	96.0	97.7	WHO 2011[18]
	Rapid antigen	Genital fluid	1	92.5	97.5	Gaydos 2017[34]
	Wet mount	Genital fluid	5	44.0	100.0	WHO 2011[18]
	Wet mount	Urine	1	44.0	100.0	Performance of urine wet mount and urethral swab wet mount assumed equivalent due to insufficient performance data, although urine wet mount expected to be substantially less sensitive, based on WHO 2011[18] and Hobbs 2006[20]
	Culture or NAAT	Genital fluid and urine	1	44.0	97.7	Combined test estimate*
	Culture and wet mount	Genital fluid	2	44.0	100.0	Combined test estimate*
	Culture or wet mount	Urine	1	44.0	100.0	Combined test estimate*

Number of studies indicates the total count of studies included within the systematic review that used each diagnostic test and specimen combination for the detection of a specific pathogen.

* Combined sensitivity and specificity estimate determined for studies reporting two diagnostic tests to identify a pathogen. If either test could be positive, the lower sensitivity and lower specificity values were used from the two tests. If both tests had to be positive, the lower sensitivity and higher specificity values were used from the two tests.

Table G: Performance of diagnostic tests among adults with genital ulcer.

Pathogen	Test	Specimen	Number studies	Sensitivity (%)	Specificity (%)	Source
HD	Culture	Genital ulcer	30	73.0	100.0	WHO 1999[17]
	NAAT	Genital ulcer	28	95.1	99.9	Paz-Bailey 2005[35]
	Culture or NAAT	Genital ulcer	1	73.0	99.9	Combined test estimate*
	Culture and NAAT	Genital ulcer	1	73.0	100.0	Combined test estimate*
HSV	Culture	Genital ulcer	16	75.0	100.0	Legoff 2014[36]
	DFA	Genital ulcer	1	80.0	95.0	Legoff 2014[36]
	NAAT	Genital ulcer	14	98.0	100.0	Legoff 2014[36]
HSV-1	DFA	Genital ulcer	1	80.0	95.0	Legoff 2014[36]
	NAAT	Genital ulcer	14	99.0	99.0	Arshad 2019[37]
HSV-2	DFA	Genital ulcer	2	80.0	95.0	Legoff 2014[36]
	NAAT	Genital ulcer	21	100.0	98.5	Arshad 2019[37]
LGV	DFA	Genital ulcer	2	95.5	99.4	Diagnostic Hybrids 2008[38]
	NAAT	Genital ulcer	8	95.5	98.5	Touati 2021[39]
TP	Darkfield	Genital ulcer	10	80.0	98.5	WHO 1999[17]
	DFA	Genital ulcer	1	86.5	94.5	Peeling 2004[40]
	NAAT	Genital ulcer	30	91.0	99.0	WHO 1999[17]
	Non-treponemal	Blood	6	96.6	97.5	Mean sensitivity and specificity, from Hambie 1983[41], Perryman 1982[42], and Parham 1984[43] regardless of titre due to varied reporting
	Treponemal	Blood	3	93.1	99.4	Mean sensitivity and specificity from Cantor 2016[44], Sena 2010[45], and Park 2019[46]
	Non-treponemal and treponemal	Blood	20	100.0	100.0	Estimated performance; non-treponemal test performance regardless of titre due to varied reporting
	Darkfield or treponemal	Genital ulcer and blood	1	80.0	95.7	Combined test estimate*
	Darkfield, or non-treponemal and treponemal	Genital ulcer and blood	1	80.0	98.5	Combined test estimate*
	NAAT, or non-treponemal and treponemal	Genital ulcer and blood	1	91.0	99.0	Combined test estimate*

Number of studies indicates the total count of studies included within the systematic review that used each diagnostic test and specimen combination for the detection of a specific pathogen.

* Combined sensitivity and specificity estimate determined for studies reporting two diagnostic tests to identify a pathogen. If either test could be positive, the lower sensitivity and lower specificity values were used from the two tests. If both tests had to be positive, the lower sensitivity and higher specificity values were used from the two tests.

Table H: Study critical appraisal tool.

Criteria	Category	Definition of yes	Yes	No	Unclear
1. Was the study specifically designed to estimate the outcome/s of interest?	Study design	The primary objective of the study was to determine sexually transmitted / reproductive tract infection aetiology among individuals symptomatic with genital ulcer, vaginal discharge and/or urethral discharge.	☐	☐	☐
2. Were the criteria for inclusion in the sample clearly defined?	Selection bias	The inclusion/exclusion criteria clearly define the population being recruited into the study. This includes their sex, age group (age range, or classified as youth or adult), and population type (e.g. general population, key population, symptomatic clinic attendee).	☐	☐	☐
3. Were study participants and their setting described in sufficient detail to determine representativeness of the target population?	Selection bias	There is a clear description of study participant characteristics, including their sex, age group and HIV status. The study site locations are described. The name of the health facility is reported, or details of the facility are described in terms of urban/rural and sector (private, government, non-profit) classification.	☐	☐	☐
4. Were study participants recruited in an appropriate way?	Selection bias	The study used either consecutive or random sampling methods for participant recruitment. If it is claimed that a random sample was chosen, the method of randomization should be stated. It is acceptable if studies do not say 'consecutive' but instead describe consecutive enrolment, i.e., 'all patients from …. till …. were included'.	☐	☐	☐
5. Was the participation rate adequate?	Selection bias	The participation rate in the study was at least 80%. Participation is defined as providing specimens for STI testing, among those identified as having symptoms.	☐	☐	☐
6. Were objective, standard criteria used to define participant symptoms?	Measurement bias	There is a clear, consistently applied definition to identify participants with the symptom(s) of focus in the study. Specifically, all participants were examined by a healthcare provider to identify their symptoms. The classification of symptoms is not based solely on participant self-reporting.	☐	☐	☐
7. Were the outcomes measured in a reliable and consistent way?	Measurement bias	All data were collected directly from the participants. Proxy data (i.e. medical records) were not used in any instances. The same mode of sample collection and diagnosis was used for all participants.	☐	☐	☐
8. Were multiple pathogens assessed to prevent potential misdiagnoses?	Measurement bias	Symptomatic participants were tested for at least 3 different sexually transmitted / reproductive tract infection pathogens.	☐	☐	☐
9. Was the sample size sufficient to measure the outcome?	Study precision	The sample size is justified through a calculation, or the sample includes at least 100 participants.	☐	☐	☐
10. Was the reporting of the outcome(s) unambiguous?	Reporting quality	The numerators, denominator(s) and proportion are clearly reported for each infection. The results are internally consistent. The reported proportion was not different when calculated from reported numerators and denominators.	☐	☐	☐

Tool adapted from the Joanna Briggs Institute appraisal tool for prevalence studies.[47]

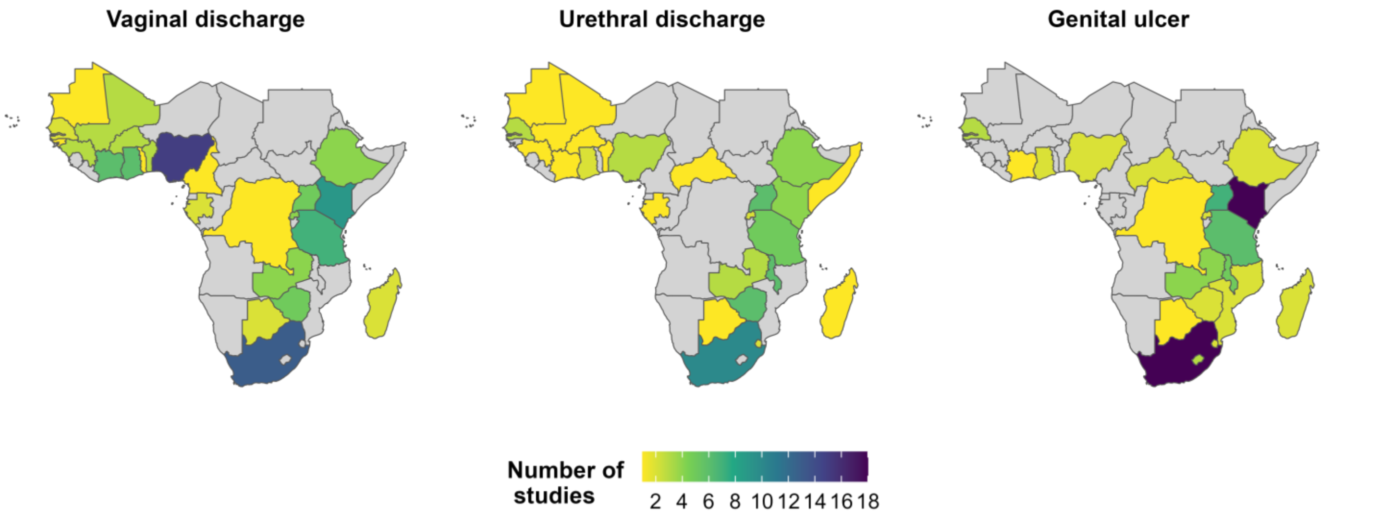

Fig A: Number of studies included per symptom by country in sub-Saharan Africa.

Number of studies among individuals with vaginal discharge, urethral discharge, and genital ulcer that were included in the systematic review. Total studies were 87 for vaginal discharge, 55 for urethral discharge, and 80 for genital ulcer. Source for base map data is Natural Earth.[48]

Table I: Summary of participant and study characteristics for studies included in the analysis of regional trends in aetiology, among adults of mixed or unmeasured HIV status.

		Vaginal discharge (N=78)	Urethral discharge (N=50)	Genital ulcer (N=74)	All symptoms (N=176)
**Population characteristics**					
**Population group**[1]	Symptomatic clinic attendee	54 (69.2%)	45 (90.0%)	59 (79.7%)	136 (77.3%)
	General	24 (30.8%)	2 (4.0%)	5 (6.8%)	30 (17.0%)
	Higher-risk general	2 (2.6%)	3 (6.0%)	7 (9.5%)	10 (5.7%)
	Key populations	6 (7.7%)	1 (2.0%)	7 (9.5%)	13 (7.4%)
	Youth	-	-	-	-
**Sex**	Male	-	50 (100.0%)	19 (25.7%)	53 (30.1%)
	Female	78 (100.0%)	-	19 (25.7%)	79 (44.9%)
	Mixed	-	-	36 (48.6%)	44 (25.0%)
**Mean or median age**	< 25 years	7 (9.0%)	2 (4.0%)	1 (1.4%)	7 (4.0%)
	≥ 25 years	17 (21.8%)	12 (24.0%)	21 (28.4%)	45 (25.6%)
	NR	54 (69.2%)	36 (72.0%)	52 (70.3%)	124 (70.5%)
**HIV prevalence**	< 25%	6 (7.7%)	1 (2.0%)	4 (5.4%)	11 (6.3%)
	≥ 25%	8 (10.3%)	6 (12.0%)	34 (45.9%)	40 (22.7%)
	NR	64 (82.1%)	43 (86.0%)	36 (48.6%)	125 (71.0%)
**Study characteristics**					
**Region**[1]	Central Africa	4 (5.1%)	2 (4.0%)	1 (1.4%)	6 (3.4%)
	Eastern Africa	30 (38.5%)	27 (54.0%)	44 (59.5%)	82 (46.6%)
	Southern Africa	10 (12.8%)	11 (22.0%)	22 (29.7%)	41 (23.3%)
	Western Africa	33 (42.3%)	9 (18.0%)	6 (8.1%)	45 (25.6%)
	Multiple (not stratified)	1 (1.3%)	1 (2.0%)	1 (1.4%)	2 (1.1%)
**Study midpoint year**[1,2]	<1990	8 (10.3%)	9 (18.0%)	18 (24.3%)	32 (18.2%)
	1990 – 1999	19 (24.4%)	16 (32.0%)	34 (45.9%)	60 (34.1%)
	2000 – 2009	27 (34.6%)	14 (28.0%)	17 (23.0%)	50 (28.4%)
	2010 – 2023	27 (34.6%)	12 (24.0%)	9 (12.2%)	40 (22.7%)
**Study design**	Cross sectional	67 (85.9%)	42 (84.0%)	58 (78.4%)	148 (84.1%)
	Cohort baseline	9 (11.5%)	7 (14.0%)	12 (16.2%)	22 (12.5%)
	RCT baseline	2 (2.6%)	1 (2.0%)	4 (5.4%)	6 (3.4%)
**Sampling method**	Non-probability based	67 (85.9%)	48 (96.0%)	70 (94.6%)	160 (90.9%)
	Probability based	5 (6.4%)	1 (2.0%)	3 (4.1%)	8 (4.5%)
	NR	6 (7.7%)	1 (2.0%)	1 (1.4%)	8 (4.5%)
**Sample size**[3]	< 100	22 (28.2%)	16 (32.0%)	43 (58.1%)	68 (38.6%)
	≥ 100	56 (71.8%)	34 (68.0%)	31 (41.9%)	108 (61.4%)
**Number pathogens extracted per study**	1	25 (32.1%)	18 (36.0%)	20 (27.0%)	52 (29.5%)
	2 - 3	29 (37.2%)	24 (48.0%)	37 (50.0%)	79 (44.9%)
	4 - 6	24 (30.8%)	8 (16.0%)	17 (23.0%)	45 (25.6%)

N = number of studies.

[1]The same study is included in more than one subcategory when it reports across different variable levels or multiple variable levels are relevant.

[2] Midpoint year between start and end dates of data collection. If dates were not reported, year of publication was used as a proxy.

[3] Sample size defined as the number of symptomatic individuals tested in the study. If a study used different samples across tests, the largest sample size is reported.

Table J: Summary of participant and study characteristics for studies included in the analysis to assess the effect of HIV status, among adults of known HIV status.

		Vaginal discharge (N=12)	Urethral discharge (N=6)	Genital ulcer (N=28)	All symptoms (N=42)
**Population characteristics**					
**Population group**[1]	Symptomatic clinic attendee	10 (83.3%)	6 (100.0%)	24 (85.7%)	36 (85.7%)
	General	1 (8.3%)	-	1 (3.6%)	2 (4.8%)
	Higher-risk general	-	-	2 (7.1%)	2 (4.8%)
	Key populations	1 (8.3%)	-	3 (10.7%)	4 (9.5%)
	Youth	-	-	-	-
**Sex**[1]	Male	-	6 (100.0%)	11 (39.3%)	18 (42.9%)
	Female	12 (100.0%)	-	9 (32.1%)	16 (38.1%)
	Mixed	-	-	12 (42.9%)	12 (28.6%)
**HIV status**[1]	Positive	8 (66.7%)	5 (83.3%)	27 (96.4%)	38 (90.5%)
	Negative	11 (91.7%)	5 (83.3%)	25 (89.3%)	36 (85.7%)
**Study characteristics**					
**Region**[1]	Central Africa	-	-	-	-
	Eastern Africa	5 (41.7%)	4 (66.7%)	16 (57.1%)	22 (52.4%)
	Southern Africa	4 (33.3%)	2 (33.3%)	11 (39.3%)	16 (38.1%)
	Western Africa	3 (25.0%)	-	2 (7.1%)	5 (11.9%)
**Study midpoint year**[1,2]	<1990	-	1 (16.7%)	2 (7.1%)	3 (7.1%)
	1990 – 1999	-	1 (16.7%)	13 (46.4%)	14 (33.3%)
	2000 – 2009	5 (41.7%)	2 (33.3%)	8 (28.6%)	13 (31%)
	2010 – 2023	8 (66.7%)	3 (50%)	7 (25%)	14 (33.3%)
**Study design**	Cross sectional	8 (66.7%)	4 (66.7%)	20 (71.4%)	28 (66.7%)
	Cohort baseline	2 (16.7%)	2 (33.3%)	5 (17.9%)	9 (21.4%)
	RCT baseline	2 (16.7%)	-	3 (10.7%)	5 (11.9%)
**Sampling method**	Non-probability based	9 (75.0%)	6 (100.0%)	25 (89.3%)	37 (88.1%)
	Probability based	3 (25.0%)	-	3 (10.7%)	5 (11.9%)
**Sample size**[3]	< 100	3 (25.0%)	2 (33.3%)	15 (53.6%)	19 (45.2%)
	≥ 100	9 (75.0%)	4 (66.7%)	13 (46.4%)	23 (54.8%)
**Number pathogens extracted per study**	1	2 (16.7%)	-	4 (14.3%)	6 (14.3%)
	2 - 3	3 (25.0%)	4 (66.7%)	16 (57.1%)	22 (52.4%)
	4 - 6	7 (58.3%)	2 (33.3%)	8 (28.6%)	14 (33.3%)

N = number of studies.

[1]The same study is included in more than one subcategory when it reports across different variable levels or multiple variable levels are relevant.

[2] Midpoint year between start and end dates of data collection. If dates were not reported, year of publication was used as a proxy.

[3] Sample size defined as the number of symptomatic individuals tested in the study. If a study used different samples across tests, the largest sample size is reported.

Table K: Summary of participant and study characteristics for studies included in the analysis to assess the effect of age, among youth < 25 years and adults ≥ 25 years.

		Vaginal discharge (N=17)	Urethral discharge (N=7)	Genital ulcer (N=7)	All symptoms (N=24)
**Population characteristics**					
**Population group**[1]	Symptomatic clinic attendee	14 (82.4%)	6 (85.7%)	6 (85.7%)	20 (83.3%)
	General	1 (5.9%)	-	1 (14.3%)	2 (8.3%)
	Higher-risk general	-	-	-	-
	Key populations	1 (5.9%)	-	-	1 (4.2%)
	Youth	15 (88.2%)	7 (100.0%)	6 (85.7%)	20 (83.3%)
**Sex**	Male	-	7 (100.0%)	1 (14.3%)	4 (16.7%)
	Female	17 (100.0%)	-	1 (14.3%)	14 (58.3%)
	Mixed	-	-	5 (71.4%)	6 (25%)
**Age group**[1]	< 25 years	15 (88.2%)	7 (100.0%)	6 (85.7%)	21 (87.5%)
	≥ 25 years	13 (76.5%)	3 (42.9%)	4 (57.1%)	18 (75%)
**Study characteristics**					
**Region**[1]	Central Africa	-	-	-	-
	Eastern Africa	6 (35.3%)	5 (71.4%)	5 (71.4%)	11 (45.8%)
	Southern Africa	1 (5.9%)	1 (14.3%)	1 (14.3%)	1 (4.2%)
	Western Africa	10 (58.8%)	1 (14.3%)	-	11 (45.8%)
	Multiple (not stratified)	-	-	1 (14.3%)	1 (4.2%)
**Study midpoint year**[1,2]	<1990	1 (5.9%)	1 (14.3%)	-	2 (8.3%)
	1990 – 1999	2 (11.8%)	3 (42.9%)	1 (14.3%)	3 (12.5%)
	2000 – 2009	10 (58.8%)	3 (42.9%)	5 (71.4%)	14 (58.3%)
	2010 – 2023	5 (29.4%)	1 (14.3%)	2 (28.6%)	6 (25%)
**Study design**	Cross sectional	14 (82.4%)	7 (100%)	4 (57.1%)	18 (75%)
	Cohort baseline	2 (11.8%)	-	1 (14.3%)	3 (12.5%)
	RCT baseline	1 (5.9%)	-	2 (28.6%)	3 (12.5%)
**Sampling method**	Non-probability based	13 (76.5%)	5 (71.4%)	6 (85.7%)	20 (83.3%)
	Probability based	2 (11.8%)	2 (28.6%)	1 (14.3%)	2 (8.3%)
	NR	2 (11.8%)	-	-	2 (8.3%)
**Sample size**[3]	< 100	4 (23.5%)	5 (71.4%)	3 (42.9%)	7 (29.2%)
	≥ 100	13 (76.5%)	2 (28.6%)	4 (57.1%)	17 (70.8%)
**Number pathogens extracted per study**	1	5 (29.4%)	3 (42.9%)	2 (28.6%)	6 (25%)
	2 - 3	5 (29.4%)	3 (42.9%)	1 (14.3%)	8 (33.3%)
	4 - 6	7 (41.2%)	1 (14.3%)	4 (57.1%)	10 (41.7%)

N = number of studies.

[1]The same study is included in more than one subcategory when it reports across different variable levels or multiple variable levels are relevant.

[2] Mid-point year between start and end dates of data collection. If dates were not reported, year of publication was used as a proxy.

[3] Sample size defined as the number of symptomatic individuals tested in the study. If a study used different samples across tests, the largest sample size is reported.

Table L: Generalized linear mixed-effects models on the odds of vaginal discharge, urethral discharge, and genital ulcer among adults of mixed or unmeasured HIV status in sub-Saharan Africa.

Vaginal discharge

Variable	aOR (95% CI)
BV	1.08 (0.51-2.29)
CA	0.45 (0.13-1.54)
CS	1.57 (0.66-3.74)
CT	0.20 (0.10-0.41)
MG	0.06 (0.02-0.15)
NG	0.09 (0.04-0.18)
TV	0.17 (0.08-0.32)
None	0.34 (0.14-0.81)
BV:year	1.01 (0.96-1.06)
CA:year	1.01 (0.95-1.06)
CS:year	1.10 (1.05-1.15)
CT:year	1.03 (0.99-1.08)
MG:year	0.99 (0.90-1.10)
NG:year	0.99 (0.96-1.03)
TV:year	0.94 (0.91-0.97)
None:year	1.00 (0.94-1.05)
**Random effects**	
Standard deviation and correlation	
σ_{00} region:rti	0.51
σ_{00} observation	0.96
σ_{11} region:rti.year	0.02
ρ_{01} region:rti	0.53
Variance	
τ^2 fixed & random	1.20 (56.38%)
τ^2 dispersion	0.91 (42.98%)
τ^2 distribution	0.01 (0.64%)
τ^2 total	2.12 (100.0%)
Number groups	
N region	3
N observation	339

Urethral discharge

Variable	aOR (95% CI)
CT	0.28 (0.19-0.42)
MG	0.06 (0.04-0.09)
NG	3.48 (2.46-4.93)
TV	0.03 (0.02-0.04)
None	0.11 (0.07-0.17)
CT:year	1.02 (0.99-1.06)
MG:year	0.95 (0.91-1.00)
NG:year	1.01 (0.99-1.03)
TV:year	0.93 (0.90-0.96)
None:year	0.95 (0.91-0.99)
**Random effects**	
Standard deviation and correlation	
σ_{00} region:rti	0.06
σ_{00} observation	0.88
σ_{11} region:rti.year	0.01
ρ_{01} region:rti	-0.94
Variance	
τ^2 fixed & random	2.70 (77.23%)
τ^2 dispersion	0.78 (22.25%)
τ^2 distribution	0.02 (0.52%)
τ^2 total	3.49 (100.0%)
Number groups	
N region	3
N observation	217

Genital ulcer

Variable	aOR (95% CI)
HD	0.01 (0.01-0.02)
HSV	1.73 (1.04-2.90)
HSV-1	0.01 (0.01-0.03)
HSV-2	1.26 (0.83-1.91)
LGV	0.02 (0.01-0.04)
TP	0.10 (0.07-0.14)
None	0.60 (0.42-0.85)
HD:year	0.85 (0.83-0.86)
HSV:year	1.08 (1.06-1.11)
HSV-1:year	1.00 (0.95-1.06)
HSV-2:year	1.01 (0.98-1.04)
LGV:year	0.93 (0.89-0.96)
TP:year	0.98 (0.96-0.99)
None:year	1.02 (1.00-1.04)
HD:male	1.98 (1.27-3.08)
HSV:male	0.68 (0.39-1.17)
HSV-1:male	1.37 (0.51-3.73)
HSV-2:male	0.65 (0.38-1.13)
LGV:male	0.92 (0.44-1.91)
TP:male	0.79 (0.51-1.24)
None:male	1.21 (0.82-1.79)
HD:male and female	2.49 (1.39-4.45)
HSV:male and female	0.72 (0.34-1.52)
HSV-1:male and female	1.92 (0.59-6.30)
HSV-2:male and female	0.68 (0.28-1.65)
LGV:male and female	1.45 (0.42-5.06)
TP:male and female	0.92 (0.56-1.53)
None:male and female	0.88 (0.50-1.53)
**Random effects**	
Standard deviation and correlation	
σ_{00} region:rti	<0.001
σ_{00} observation	0.86
σ_{11} region:rti.year	<0.001
ρ_{01} region:rti	0.98
Variance	
τ^2 fixed & random	2.17 (73.95%)
τ^2 dispersion	0.73 (25.03%)
τ^2 distribution	0.03 (1.02%)
τ^2 total	2.93 (100.0%)
Number groups	
N region	3
N observation	483

Midpoint year of study is centred at 2015.

Odds estimated using generalised linear mixed-effects model for each symptom with fixed effects for RTI and the interaction of RTI with year and sex (genital ulcer only), random intercepts and slopes per year for the interaction of RTI and region, and observation-level random intercepts.

aOR: adjusted odds ratio, 95% CI: 95% confidence interval, σ_{00}: random intercept standard deviation, σ_{11}: random slope standard deviation, ρ_{01}: correlation between intercept and slope, τ^2: variance attributed to fixed and random effects, dispersion (observation-level) effects, and the binomial distribution, N: number.

BV: Bacterial vaginosis, CA: *Candida albicans*, CS: *Candida* species (any), CT: *Chlamydia trachomatis*, HD: *Haemophilus ducreyi*, HSV: Herpes simplex virus (unspecified), HSV-1: Herpes simplex virus type 1, HSV-2: Herpes simplex virus type 2, LGV: lymphogranuloma venereum, MG: *Mycoplasma genitalium*, NG: *Neisseria gonorrhoeae*, TP: *Treponema pallidum*, TV: *Trichomonas vaginalis*, None: unknown aetiology.

Table M: Generalized linear mixed-effects models on the odds of vaginal discharge, urethral discharge, and genital ulcer among adults of known HIV status in sub-Saharan Africa.

Vaginal discharge

Variable	aOR (95% CI)
BV	0.55 (0.33-0.92)
CS	0.81 (0.46-1.42)
CT	0.14 (0.09-0.25)
MG	0.04 (0.02-0.07)
NG	0.11 (0.06-0.18)
TV	0.11 (0.07-0.18)
None	0.25 (0.14-0.45)
BV:year	1.01 (0.99-1.04)
CS:year	0.99 (0.95-1.03)
CT:year	1.04 (1.01-1.07)
MG:year	1.00 (0.96-1.04)
NG:year	1.02 (0.99-1.05)
TV:year	0.94 (0.92-0.97)
None:year	1.01 (0.97-1.04)
BV:HIV-positive	1.85 (1.38-2.46)
CS:HIV-positive	0.50 (0.34-0.73)
CT:HIV-positive	0.95 (0.70-1.29)
MG:HIV-positive	2.32 (1.59-3.39)
NG:HIV-positive	1.61 (1.18-2.18)
TV:HIV-positive	2.15 (1.61-2.87)
None:HIV-positive	0.72 (0.53-0.99)
**Random effects**	
Standard deviation	
σ_{00} region:rti	0.39
σ_{00} observation	0.42
Variance	
τ^2 fixed & random	0.90 (81.92%)
τ^2 dispersion	0.18 (16.02%)
τ^2 distribution	0.02 (2.06%)
τ^2 total	1.10 (100.0%)
Number groups	
N region	3
N observation	275

Urethral discharge

Variable	aOR (95% CI)
CT	0.40 (0.30-0.53)
MG	0.06 (0.05-0.08)
NG	4.85 (3.66-6.43)
TV	0.02 (0.01-0.03)
None	0.09 (0.07-0.12)
CT:year	1.03 (1.00-1.06)
MG:year	0.95 (0.91-1.00)
NG:year	1.05 (1.02-1.08)
TV:year	0.89 (0.86-0.93)
None:year	0.92 (0.88-0.96)
CT:HIV-positive	0.54 (0.37-0.81)
MG:HIV-positive	1.04 (0.66-1.64)
NG:HIV-positive	1.06 (0.71-1.56)
TV:HIV-positive	1.87 (1.07-3.25)
None:HIV-positive	1.40 (0.92-2.14)
**Random effects**	
Standard deviation	
σ_{00} region:rti	<0.001
σ_{00} observation	0.54
Variance	
τ^2 fixed & random	3.24 (91.10%)
τ^2 dispersion	0.29 (8.21%)
τ^2 distribution	0.02 (0.68%)
τ^2 total	3.56 (100.0%)
Number groups	
N region	3
N observation	177

Genital ulcer

Variable	aOR (95% CI)
HD	0.03 (0.02-0.05)
HSV	0.94 (0.57-1.56)
HSV-1	0.03 (0.01-0.06)
HSV-2	1.20 (0.79-1.83)
LGV	0.04 (0.02-0.07)
TP	0.12 (0.08-0.17)
None	0.72 (0.52-1.01)
HD:year	0.88 (0.86-0.89)
HSV:year	1.05 (1.03-1.07)
HSV-1:year	1.04 (0.96-1.13)
HSV-2:year	0.99 (0.96-1.02)
LGV:year	0.99 (0.94-1.05)
TP:year	1.00 (0.99-1.02)
None:year	1.01 (0.99-1.03)
HD:male	1.71 (1.11-2.65)
HSV:male	1.01 (0.62-1.63)
HSV-1:male	0.74 (0.28-1.96)
HSV-2:male	0.44 (0.27-0.73)
LGV:male	0.71 (0.37-1.34)
TP:male	1.42 (0.96-2.09)
None:male	0.86 (0.59-1.24)
HD:male and female	1.61 (0.97-2.68)
HSV:male and female	0.61 (0.31-1.18)
HSV-1:male and female	2.04 (0.63-6.63)
HSV-2:male and female	0.47 (0.26-0.86)
LGV:male and female	2.50 (0.95-6.59)
TP:male and female	1.55 (0.96-2.49)
None:male and female	0.79 (0.50-1.26)
HD:HIV-positive	0.83 (0.58-1.19)
HSV:HIV-positive	1.78 (1.15-2.75)
HSV-1:HIV-positive	1.17 (0.52-2.63)
HSV-2:HIV-positive	1.63 (1.07-2.46)
LGV:HIV-positive	0.95 (0.53-1.72)
TP:HIV-positive	0.89 (0.64-1.24)
None:HIV-positive	0.66 (0.48-0.92)
**Random effects**	
Standard deviation	
σ_{00} region:rti	<0.001
σ_{00} observation	0.72
Variance	
τ^2 fixed & random	1.78 (75.64%)
τ^2 dispersion	0.52 (22.13%)
τ^2 distribution	0.05 (2.23%)
τ^2 total	2.36 (100.0%)
Number groups	
N region	3
N observation	512

Midpoint year of study is centred at 2015.

Odds estimated using generalised linear mixed-effects model for each symptom with fixed effects for RTI and the interaction of RTI with year, HIV status, and sex (genital ulcer only), random intercepts for the interaction of RTI and region, and observation-level random intercepts.

aOR: adjusted odds ratio, 95% CI: 95% confidence interval, σ_{00}: random intercept standard deviation, τ^2: variance attributed to fixed and random effects, dispersion (observation-level) effects, and the binomial distribution, N: number.

BV: Bacterial vaginosis, CS: *Candida* species (any), CT: *Chlamydia trachomatis*, HD: *Haemophilus ducreyi*, HSV: Herpes simplex virus (unspecified), HSV-1: Herpes simplex virus type 1, HSV-2: Herpes simplex virus type 2, LGV: lymphogranuloma venereum, MG: *Mycoplasma genitalium*, NG: *Neisseria gonorrhoeae*, TP: *Treponema pallidum*, TV: *Trichomonas vaginalis*, None: unknown aetiology.

Table N: Generalized linear mixed-effects models on the odds of vaginal discharge, urethral discharge, and genital ulcer among youth under 25 years and adults 25 years and older in sub-Saharan Africa.

Vaginal discharge

Variable	aOR (95% CI)
BV	0.69 (0.29-1.60)
CA	0.49 (0.07-3.53)
CS	0.49 (0.19-1.25)
CT	0.08 (0.03-0.19)
MG	0.04 (0.01-0.13)
NG	0.05 (0.02-0.13)
TV	0.11 (0.04-0.25)
None	0.36 (0.13-0.98)
BV:year	1.05 (1.02-1.09)
CA:year	1.05 (0.96-1.14)
CS:year	1.01 (0.96-1.05)
CT:year	1.04 (1.01-1.08)
MG:year	0.99 (0.95-1.03)
NG:year	1.00 (0.96-1.03)
TV:year	0.92 (0.89-0.95)
None:year	0.98 (0.95-1.02)
BV:<25 years	1.12 (0.84-1.51)
CA:<25 years	2.15 (0.74-6.26)
CS:<25 years	1.82 (1.25-2.66)
CT:<25 years	2.39 (1.72-3.32)
MG:<25 years	1.99 (1.33-2.98)
NG:<25 years	1.65 (1.20-2.27)
TV:<25 years	0.85 (0.63-1.13)
None:<25 years	0.67 (0.49-0.92)
**Random effects**	
Standard deviation	
σ_{00} region:rti	0.68
σ_{00} observation	0.42
Variance	
τ^2 fixed & random	1.47 (87.89%)
τ^2 dispersion	0.18 (10.72%)
τ^2 distribution	0.02 (1.39%)
τ^2 total	1.67 (100.0%)
Number groups	
N region	3
N observation	281

Urethral discharge

Variable	aOR (95% CI)
CT	0.28 (0.22-0.37)
MG	0.06 (0.04-0.07)
NG	3.75 (2.92-4.82)
TV	0.03 (0.02-0.04)
None	0.10 (0.08-0.13)
CT:year	1.05 (1.01-1.08)
MG:year	0.92 (0.88-0.96)
NG:year	1.05 (1.03-1.07)
TV:year	0.94 (0.92-0.96)
None:year	0.90 (0.86-0.94)
CT:<25 years	1.59 (1.09-2.30)
MG:<25 years	1.01 (0.65-1.56)
NG:<25 years	1.39 (0.96-2.01)
TV:<25 years	1.35 (0.79-2.31)
None:<25 years	0.73 (0.48-1.11)
**Random effects**	
Standard deviation	
σ_{00} region:rti	<0.001
σ_{00} observation	0.47
Variance	
τ^2 fixed & random	3.08 (92.55%)
τ^2 dispersion	0.23 (6.78%)
τ^2 distribution	0.02 (0.67%)
τ^2 total	3.33 (100.0%)
Number groups	
N region	3
N observation	163

Genital ulcer

Variable	aOR (95% CI)
HD	0.02 (0.02-0.04)
HSV	1.25 (0.78-1.99)
HSV-1	0.01 (0.00-0.05)
HSV-2	1.50 (1.11-2.02)
LGV	0.02 (0.01-0.04)
TP	0.08 (0.06-0.11)
None	0.57 (0.46-0.71)
HD:year	1.01 (0.95-1.08)
HSV:year	0.99 (0.91-1.07)
HSV-1:year	1.09 (0.88-1.36)
HSV-2:year	0.97 (0.93-1.02)
LGV:year	1.02 (0.95-1.10)
TP:year	1.10 (1.07-1.14)
None:year	0.97 (0.95-1.00)
HD:Male	0.65 (0.36-1.16)
HSV:Male	0.89 (0.61-1.30)
HSV-1:Male	0.81 (0.30-2.18)
HSV-2:Male	0.64 (0.43-0.96)
LGV:Male	0.76 (0.41-1.41)
TP:Male	2.01 (1.37-2.94)
None:Male	0.93 (0.71-1.22)
HD:Male and female	0.40 (0.11-1.48)
HSV-1:Male and female	13.76 (0.35-540)
HSV-2:Male and female	0.31 (0.16-0.59)
TP:Male and female	2.30 (1.12-4.72)
None:Male and female	1.46 (0.84-2.53)
HD:<25 years	2.30 (1.29-4.07)
HSV:<25 years	1.01 (0.68-1.51)
HSV-1:<25 years	2.65 (0.92-7.63)
HSV-2:<25 years	1.02 (0.69-1.51)
LGV:<25 years	2.40 (1.27-4.54)
TP:<25 years	1.39 (0.96-2.04)
None:<25 years	1.12 (0.85-1.48)
**Random effects**	
Standard deviation	
σ_{00} region:rti	<0.001
σ_{00} observation	0.36
Variance	
τ^2 fixed & random	2.58 (92.78%)
τ^2 dispersion	0.13 (4.59%)
τ^2 distribution	0.07 (2.63%)
τ^2 total	2.78 (100.0%)
Number groups	
N region	3
N observation	287

Midpoint year of study is centred at 2015.

Odds estimated using generalised linear mixed-effects model for each symptom with fixed effects for RTI and the interaction of RTI with year, age group, and sex (genital ulcer only), random intercepts for the interaction of RTI and region, and observation-level random intercepts.

aOR: adjusted odds ratio, 95% CI: 95% confidence interval, σ_{00}: random intercept standard deviation, τ^2: variance attributed to fixed and random effects, dispersion (observation-level) effects, and the binomial distribution, N: number.

BV: Bacterial vaginosis, CA: *Candida albicans*, CS: *Candida* species (any), CT: *Chlamydia trachomatis*, HD: *Haemophilus ducreyi*, HSV: Herpes simplex virus (unspecified), HSV-1: Herpes simplex virus type 1, HSV-2: Herpes simplex virus type 2, LGV: lymphogranuloma venereum, MG: *Mycoplasma genitalium*, NG: *Neisseria gonorrhoeae*, TP: *Treponema pallidum*, TV: *Trichomonas vaginalis*, None: unknown aetiology.

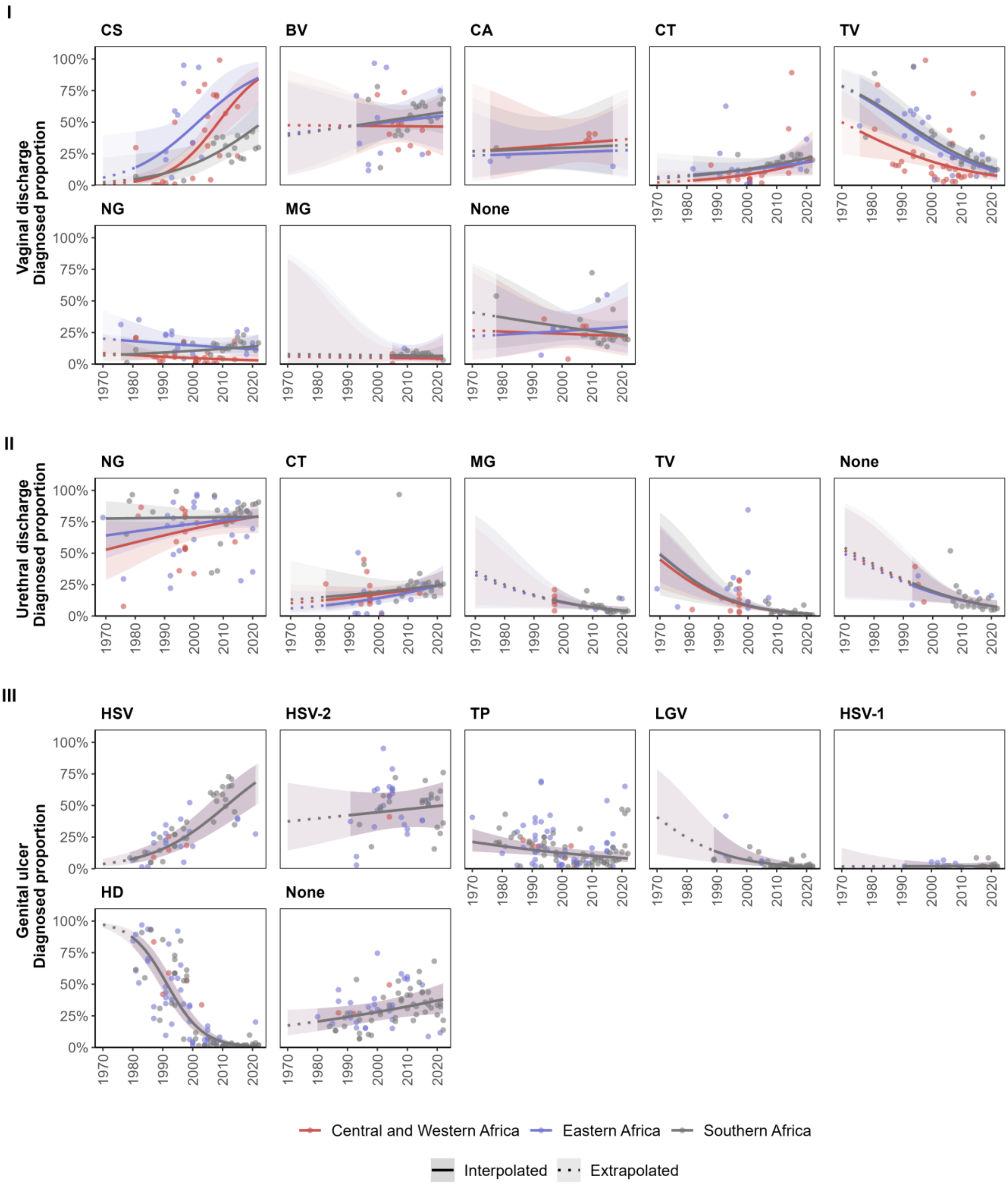

Fig B: Estimated diagnosed proportion per pathogen among symptomatic adults of mixed HIV status in sub-Saharan Africa, by region over time.

Diagnosed proportion per pathogen among individuals symptomatic with (I) vaginal discharge, (II) urethral discharge, and (III) genital ulcer. Proportions estimated using generalised linear mixed-effects model for each symptom. Lines and shaded areas represent sex-matched population-weighted mean proportions and 95% confidence intervals Solid lines and darker shading denote estimates and confidence intervals within the observed data range (interpolated), while dotted lines and lighter shading indicate estimates and confidence intervals beyond that time frame (extrapolated). Points represent study observations adjusted for diagnostic test performance.

BV: Bacterial vaginosis, CA: *Candida albicans*, CS: *Candida* species (any), CT: *Chlamydia trachomatis*, HD: *Haemophilus ducreyi*, HSV: Herpes simplex virus (unspecified), HSV-1: Herpes simplex virus type 1, HSV-2: Herpes simplex virus type 2, LGV: lymphogranuloma venereum, MG: *Mycoplasma genitalium*, NG: *Neisseria gonorrhoeae*, TP: *Treponema pallidum*, TV: *Trichomonas vaginalis*, None: unknown aetiology.

Table O: Critical appraisal of reports included in the review.

Assessment: ● Yes ● Unclear ● No

Reference	Year[1]	Country	Q1	Q2	Q3	Q4	Q5	Q6	Q7	Q8	Q9	Q10
Arya 1972[49]	1969	Uganda	Yes	Yes	No	Yes	Yes	Yes	Yes	No	Yes	Yes
Ongom 1971[50]	1970	Uganda	No	No	No	Yes	Unclear	No	Yes	No	No	No
Van Binsbergen 1980[51]	1976	Kenya	Yes	Yes	No	Yes	Unclear	No	Yes	Yes	Yes	Yes
Fakunle 1976[52]	1976*	Nigeria	No	Yes	No	Unclear	Unclear	Yes	Yes	No	Yes	Yes
Ballard 1977[53]	1977*	South Africa	Yes	Yes	No	Unclear	Unclear	Unclear	Yes	No	Yes	No
Rubin 1980[54]	1978	South Africa	No	No	No	Unclear	Unclear	Yes	Yes	Yes	Yes	No
Meheus 1982[55]	1978	Eswatini	Yes	Yes	No	Yes	Unclear	Yes	Yes	No	No	Yes
Meheus 1983[56]	1979	Eswatini	Yes	Yes	No	Yes	Yes	Yes	Yes	No	Yes	Yes
Ursi 1981[57]	1979	Eswatini	No	Yes	No	Yes	Unclear	Yes	Yes	No	Yes	Yes
Meheus 1980[58]	1979	Eswatini	Yes	Yes	No	Yes	Yes	Yes	Yes	No	No	Yes
Nsanze 1981[59]	1980	Kenya	Yes	Yes	No	No	Yes	Yes	Yes	Yes	No	Yes
Fast 1983[60]	1981	Kenya	No	No	No	Unclear	Unclear	Yes	No	No	Yes	No
Yvert 1984[61]	1981	Gabon	Yes	Yes	No	Unclear	Unclear	No	Yes	Yes	Yes	Yes
Hoosen 1981[62]	1981*	South Africa	No	Yes	No	No	Yes	No	Yes	Yes	Yes	No
Crewe-Brown 1982[63]	1981	South Africa	Yes	Yes	No	Yes	Yes	Yes	No	Yes	Yes	No
D'Costa 1985[64]	1982	Kenya	No	Yes	No	Unclear	Unclear	Yes	Yes	No	No	No
Mirza 1983[65]	1982	Kenya	Yes	Yes	No	Yes	Yes	No	Yes	Yes	Yes	Yes
Mabey 1982[66]	1982*	The Gambia	No	Yes	No	Yes	Yes	Yes	Yes	No	No	Yes
Mabey 1984[67]	1982	The Gambia	No	Yes	No	Yes	Yes	Yes	Yes	No	No	No
Plummer 1983[68]	1983*	Kenya	No	Yes	No	Unclear	Yes	Yes	Yes	No	No	No
Mauff 1983[69]	1983*	South Africa	Yes	Yes	No	No	Yes	Yes	Yes	No	No	Yes
Coovadia 1985[70]	1984	South Africa	Yes	Yes	No	Unclear	Yes	Yes	Yes	Yes	Yes	No
Greenblatt 1988[71]	1985	Kenya	No	Yes	Yes	Yes	Yes	Yes	Yes	Yes	Yes	Yes
Plummer 1985[72]	1985*	Kenya	Yes	Yes	No	No	Yes	Yes	Yes	Yes	No	No
Kreiss 1986[73]	1985	Kenya	No	Yes	Yes	Unclear	Unclear	Yes	Yes	No	No	Yes
Dangor 1989[74]	1986	South Africa	Yes	Yes	No	Yes	Unclear	Yes	No	Yes	Yes	Yes
Ismail 1990[75]	1986	Somalia	Yes	Yes	Yes	Yes	No	No	Yes	No	No	No
Meiring 1989[76]	1986	South Africa	Yes	No	No	Unclear	Unclear	No	Yes	No	Yes	No
Mabey 1987[77]	1987*	The Gambia	Yes	Yes	No	Unclear	Yes	Yes	Yes	Yes	Yes	Yes
Leclerc 1988[78]	1988*	Gabon	Yes	Yes	No	Unclear	Yes	No	Yes	No	Yes	Yes
Bogaerts 1998[79]	1989	Rwanda	Yes	Yes	Yes	Yes	Yes	Yes	Yes	Yes	Yes	No
Faye-Kette 1993[80]	1989	Côte d'Ivoire	Yes	Yes	No	Unclear	Unclear	No	Yes	Yes	Yes	No
O'Farrell 1994[81]	1989	South Africa	No	Yes	No	Unclear	Unclear	Yes	Yes	Yes	Yes	No
Daly 1994[82]	1990	Kenya	No	Yes	Yes	Yes	Yes	Yes	Yes	No	No	No
Thior 1997[83]	1990	Senegal	No	Yes	Yes	Yes	Unclear	Unclear	Yes	No	Yes	Yes

Reference	Year[1]	Country	Q1	Q2	Q3	Q4	Q5	Q6	Q7	Q8	Q9	Q10
Ndinya-Achola 1996[84]	1991	Kenya	🔴	🟢	🔴	🟡	🟡	🟢	🟢	🔴	🟢	🔴
Kamya 1995[85]	1991	Uganda	🟢	🟢	🟢	🟢	🟢	🟢	🟢	🟢	🟢	🔴
Hanson 1996[86]	1991	Zambia	🔴	🟢	🟢	🟢	🟡	🟢	🟢	🟢	🟢	🟢
LeBacq 1993[87]	1991	Zimbabwe	🟢	🟢	🟢	🟢	🔴	🟢	🟢	🟢	🔴	🟢
Kimani 1995[88]	1992	Kenya	🔴	🟢	🟢	🟢	🟢	🟢	🟢	🔴	🟢	🔴
Oni 1997[89]	1992	Nigeria	🟢	🟢	🔴	🟢	🟡	🟢	🟢	🔴	🔴	🟢
Totten 2000[90]	1992	Senegal	🟢	🔴	🔴	🟢	🟡	🟢	🟢	🟢	🔴	🟢
Htun 2007[91]	1992	South Africa	🟢	🟢	🟢	🟢	🟢	🟢	🟢	🟢	🟢	🟢
Grosskurth 1996[92]	1992	Tanzania	🟢	🟢	🔴	🟢	🟢	🟢	🟢	🔴	🟢	🟢
Tswana 1995[93]	1992	Zimbabwe	🟢	🟢	🟢	🟢	🟢	🟢	🟢	🔴	🟢	🔴
Ghys 1995[94]	1993	Côte d'Ivoire	🔴	🟢	🟢	🔴	🟡	🟢	🟢	🔴	🟢	🔴
Meless 1997[95]	1993	Ethiopia	🟢	🟢	🔴	🟡	🟡	🟡	🟢	🔴	🟢	🟢
Harms 1994[96]	1993	Madagascar	🟢	🟢	🟢	🔴	🟢	🟢	🔴	🟢	🟢	🟢
Dallabetta 1998[97]	1993	Malawi	🟢	🟢	🔴	🟢	🔴	🟢	🟢	🔴	🟢	🔴
Behets 1995[98]	1993	Malawi	🟢	🟢	🟢	🟢	🟢	🟢	🟢	🔴	🟢	🟢
Cossa 1994[99]	1993	Mozambique	🔴	🟢	🟢	🟢	🟡	🟢	🟢	🔴	🔴	🔴
Alary 1998[100]	1994	Benin	🔴	🟢	🔴	🟢	🟢	🔴	🟢	🟢	🟢	🔴
Thomas 1996[101]	1994	Kenya	🔴	🟢	🟢	🟢	🟢	🔴	🟢	🟢	🔴	🔴
King 1998[102]	1994	Kenya	🔴	🟢	🟢	🟢	🟢	🟢	🟢	🔴	🟢	🟢
Htun 1998[103]	1994	Lesotho	🔴	🟢	🔴	🟢	🟢	🟢	🟢	🟢	🟢	🟢
Morse 1997[104]	1994	Lesotho	🔴	🟢	🟢	🟢	🟢	🟢	🟢	🟢	🟢	🟢
Lo 1997[105]	1994	Mauritania	🟢	🔴	🔴	🟡	🟡	🔴	🟢	🟢	🟢	🔴
Chen 2000[106]	1994	South Africa	🟢	🟢	🟢	🟢	🟢	🟢	🟢	🟢	🟢	🟢
Schneider 1998[107]	1994	South Africa	🔴	🟢	🔴	🟢	🟡	🟢	🟢	🔴	🟢	🔴
Pillay 1994[108]	1994*	South Africa	🟢	🟢	🔴	🟢	🟡	🟡	🔴	🔴	🟢	🟢
Mayaud 1997[109]	1994	Tanzania	🔴	🟢	🔴	🟢	🟡	🟢	🟢	🔴	🔴	🔴
Aseffa 1998[110]	1995	Ethiopia	🔴	🟢	🟢	🟢	🟡	🟢	🟢	🔴	🔴	🟢
MacDonald 2001[111]	1995	Kenya	🔴	🟢	🟢	🟡	🟡	🟢	🟢	🔴	🟢	🟢
Jackson 1997[112]	1995	Kenya	🔴	🟢	🟢	🟢	🟡	🟢	🟢	🟢	🔴	🟢
Gateau 1996[113]	1995	Madagascar	🔴	🔴	🔴	🟢	🟢	🟢	🟢	🔴	🔴	🔴
Bakare 2002[114]	1995	Nigeria	🟢	🟢	🔴	🟢	🟡	🟢	🟢	🟢	🟢	🔴
Ramuthaga 1995[115]	1995*	South Africa	🔴	🔴	🔴	🟡	🟡	🟢	🔴	🔴	🟢	🟢
Ahmed 1995[116]	1995*	Zambia	🔴	🔴	🔴	🟡	🟡	🟢	🟢	🔴	🔴	🟢
Latif 1999[117]	1995	Zimbabwe	🔴	🟢	🟢	🟢	🟢	🟢	🟢	🟢	🟢	🟢
Farina 2000[118]	1996	Comoros Islands	🔴	🟢	🔴	🟡	🟢	🟢	🟢	🔴	🔴	🟢
Malonza 1999[119]	1996	Kenya	🔴	🟢	🟢	🟡	🟢	🟢	🔴	🟢	🟢	🟢
Cohen 1997[120]	1996	Malawi	🔴	🟢	🟢	🟡	🟡	🟢	🟢	🟢	🟢	🟢
Dieye 2003[121]	1996	Senegal	🔴	🟢	🔴	🟡	🔴	🔴	🟢	🔴	🔴	🔴

Reference	Year[1]	Country	Q1	Q2	Q3	Q4	Q5	Q6	Q7	Q8	Q9	Q10
Lai 2003[122]	1996	South Africa	🟢	🟢	🟢	🟢	🟢	🟢	🟢	🟢	🟢	🟢
Ballard 1996[123]	1996*	South Africa	🔴	🔴	🟢	🟡	🟡	🟢	🟢	🔴	🔴	🔴
Pepin 2001[124]	1997	Benin, Burkina Faso, Côte d'Ivoire, Ghana, Guinea, Mali, Senegal	🟢	🟢	🔴	🟢	🟡	🟢	🟢	🟢	🟢	🟢
Morency 2001[125]	1997	Central African Republic	🟢	🟢	🔴	🟢	🟢	🟢	🔴	🟢	🟢	🟢
Gomes 2001[126]	1997	Guinea-Bissau	🟢	🟢	🔴	🟡	🟢	🟢	🟢	🔴	🟢	🟢
Fonck 2000[127]	1997	Kenya	🔴	🟢	🟢	🟢	🟡	🟢	🟢	🟢	🟢	🟢
Ballard 2007[128]	1997	Lesotho, South Africa	🟢	🟢	🟢	🟡	🟡	🟢	🟢	🟢	🟢	🟢
Behets 1999[129]	1997	Madagascar	🟢	🟢	🔴	🟢	🟡	🟢	🟢	🟢	🟢	🟢
Rajagopal 1999[130]	1997	South Africa	🔴	🟢	🟢	🟡	🟡	🟢	🟢	🔴	🔴	🟢
Hoosen 1997[131]	1997*	South Africa	🟢	🟢	🟢	🟡	🟡	🔴	🟢	🟢	🔴	🔴
Ramjee 1998[132]	1997	South Africa	🔴	🟢	🟢	🔴	🟢	🟢	🟢	🔴	🔴	🔴
Paz-Bailey 2005[35]	1998	Botswana	🟢	🟢	🟢	🟢	🟡	🟢	🟢	🟢	🟢	🟢
Nzila 1991[133]	1998	Democratic Republic of Congo	🔴	🟢	🟢	🔴	🟡	🟢	🟢	🔴	🔴	🔴
Anorlu 2001[134]	1998	Nigeria	🟢	🟢	🔴	🟡	🟢	🟢	🟢	🔴	🟢	🟢
Ballard 2002[135]	1998	South Africa	🟢	🟢	🟢	🟢	🟢	🟡	🟢	🟢	🟢	🟢
Obasi 2001[136]	1998	Tanzania	🔴	🟢	🟢	🔴	🟢	🔴	🟢	🔴	🟢	🔴
Msuya 2006[137]	1999	Tanzania	🔴	🟢	🔴	🟡	🟢	🟢	🟢	🔴	🟢	🔴
Msuya 2002[138]	1999	Tanzania	🔴	🟢	🔴	🟡	🟢	🟢	🟢	🔴	🟢	🔴
Hoyo 2005[139]	1999	Malawi	🟢	🟢	🔴	🟢	🟢	🟢	🟢	🟢	🟢	🟢
Chalamilla 2006[140]	1999	Tanzania	🟢	🟢	🟢	🟢	🟡	🟢	🟢	🟢	🔴	🔴
Ahmed 2003[141]	1999	Tanzania	🟢	🟢	🟢	🟢	🟢	🟢	🟢	🟢	🟢	🟢
Mbizvo 2004[142]	1999	Zimbabwe	🔴	🟢	🟢	🟢	🟢	🟢	🟢	🔴	🔴	🔴
Vuylsteke 2003[143]	2000	Côte d'Ivoire	🔴	🟢	🔴	🟡	🟡	🔴	🟢	🟢	🔴	🔴
Price 2004[144]	2000	Malawi	🟢	🟢	🟢	🟢	🟢	🟢	🔴	🔴	🟢	🔴
Anorlu 2004[145]	2000	Nigeria	🔴	🟢	🟢	🟢	🔴	🟢	🟢	🟢	🟢	🟢
Bakare 2002[146]	2000	Nigeria	🟢	🟢	🔴	🟢	🟡	🟢	🟢	🔴	🟢	🟢
Moodley 2002[147]	2000	South Africa	🟢	🟢	🟢	🔴	🟡	🟡	🟢	🟢	🟢	🟢
Nilsen 2007[148]	2000	Tanzania	🟢	🟢	🟢	🟢	🟢	🟢	🟢	🟢	🟢	🟢
Riedner 2007[149]	2000	Tanzania	🔴	🟢	🟢	🔴	🟡	🟢	🟢	🟢	🔴	🟢
Watson-Jones 2000[150]	2000*	Tanzania	🔴	🟢	🔴	🔴	🟡	🟢	🔴	🔴	🔴	🟢
Pickering 2005[151]	2000	Uganda	🟢	🟢	🟢	🟡	🟢	🟢	🟢	🟢	🟢	🔴
Mason 2000[152]	2000*	Zimbabwe	🔴	🟢	🔴	🟡	🟡	🟢	🟢	🟢	🟢	🟢
Wolday 2004[153]	2001	Ethiopia	🔴	🟢	🟢	🟢	🟢	🟢	🟢	🟢	🔴	🟢
Pepin 2004[154]	2001	Benin, Burkina Faso, Ghana, Guinea, Mali	🟢	🟢	🔴	🟡	🟢	🟢	🟢	🟢	🟢	🟢
Romoren 2007[155]	2001	Botswana	🔴	🟢	🔴	🔴	🟡	🟢	🟢	🔴	🟢	🔴
Bankole 2001[156]	2001*	Côte d'Ivoire	🟢	🟢	🔴	🟡	🔴	🔴	🟢	🔴	🟢	🔴

Reference	Year[1]	Country	Q1	Q2	Q3	Q4	Q5	Q6	Q7	Q8	Q9	Q10
Tadesse 2001[157]	2001*	Ethiopia	🔴	🟢	🔴	🔴	🟢	🟢	🟢	🔴	🟢	🟢
Zachariah 2002[158]	2001	Malawi	🟢	🟢	🔴	🟢	🟢	🔴	🟢	🔴	🟢	🟢
Ly 2006[159]	2001	Senegal	🔴	🟢	🟢	🟡	🟡	🔴	🟢	🔴	🔴	🔴
Moodley 2003[160]	2001	South Africa	🟢	🟢	🟢	🔴	🟡	🟢	🟢	🟢	🟢	🟢
Otuonye 2004[161]	2002	Nigeria	🟢	🟢	🟢	🔴	🟡	🔴	🟢	🟢	🟢	🔴
Namkinga 2005[162]	2002	Tanzania	🟢	🟢	🟢	🟡	🟢	🟢	🟢	🔴	🟢	🟢
Mwansasu 2002[163]	2002*	Tanzania	🟢	🟢	🟢	🟢	🟢	🟢	🟢	🔴	🔴	🟢
Rassjo 2006[164]	2002	Uganda	🔴	🟢	🔴	🟢	🟡	🔴	🟢	🔴	🟢	🔴
Fayemiwo 2011[165]	2003	Nigeria	🔴	🟢	🟢	🔴	🟡	🟢	🟢	🔴	🔴	🔴
Msuya 2009[166]	2003	Tanzania	🔴	🟢	🔴	🟡	🟡	🟢	🟢	🟢	🟢	🔴
O'Farrell 2008[167]	2004	South Africa	🟢	🟢	🟢	🟢	🟢	🟢	🟢	🟢	🟢	🟢
Weiss 2011[168]	2004	Central African Republic	🔴	🟢	🟢	🟢	🟢	🟢	🟢	🟢	🟢	🟢
Legoff 2007[169]	2004	Central African Republic, Ghana	🔴	🟢	🟢	🟢	🟢	🟢	🟢	🟢	🟢	🟢
Abauleth 2006[170]	2004	Côte d'Ivoire	🟢	🟢	🔴	🟡	🟡	🔴	🟢	🟢	🟢	🟢
Mehta 2012[171]	2004	Kenya	🔴	🟢	🟢	🔴	🟢	🟢	🟢	🔴	🟢	🟢
Onyekonwu 2011[172]	2004	Nigeria	🟢	🟢	🔴	🟢	🟢	🟢	🟢	🟢	🟢	🟢
Suntoke 2009[173]	2004	Uganda	🔴	🟢	🟢	🟢	🟢	🟢	🟢	🟢	🟢	🟢
Pepin 2006[174]	2005	Ghana, Guinea, Mali, Togo	🔴	🟢	🟢	🟡	🟢	🟢	🟢	🟢	🟢	🟢
Pepin 2011[175]	2005	Ghana, Guinea, Mali, Togo	🔴	🟢	🟢	🟡	🟢	🟢	🟢	🟢	🟢	🟢
Phiri 2013[176]	2005	Malawi	🟢	🟢	🟢	🟢	🟢	🟢	🟢	🟢	🟢	🟢
Brankin 2009[177]	2005	Uganda	🔴	🟢	🔴	🔴	🟡	🟢	🟢	🟢	🔴	🟢
Zimba 2011[178]	2005	Mozambique	🟢	🟢	🟢	🟢	🟢	🟢	🟢	🟢	🔴	🟢
Paz-Bailey 2010[179]	2006	South Africa	🔴	🟢	🟢	🟡	🟢	🟢	🟢	🟢	🟢	🔴
Paz-Bailey 2009[180]	2006	South Africa	🔴	🟢	🟢	🟡	🟢	🟢	🟢	🟢	🟢	🔴
Ojuromi 2007[181]	2006	Nigeria	🟢	🟢	🔴	🟡	🟡	🔴	🟢	🟢	🟢	🟢
Nwadioha 2012[182]	2007	Nigeria	🟢	🟢	🟢	🟡	🟡	🟡	🟢	🔴	🟢	🟢
Nwadioha 2011[183]	2007	Nigeria	🟢	🟢	🟢	🟡	🟡	🟢	🟢	🔴	🟢	🟢
Bochner 2017[184]	2007	Botswana, Kenya, Rwanda, South Africa, Tanzania, Uganda	🔴	🟢	🟢	🔴	🟢	🟢	🔴	🔴	🟢	🟢
Brown 2010[185]	2007	Malawi	🔴	🟢	🔴	🟢	🟡	🟢	🟢	🔴	🟢	🟢
Nwadioha 2011[186]	2007	Nigeria	🟢	🔴	🔴	🔴	🟡	🟡	🟢	🟢	🟢	🟢
Muvunyi 2009[187]	2007	Rwanda	🟢	🟢	🔴	🟢	🔴	🔴	🟢	🔴	🔴	🔴
Moodley 2013[188]	2007	South Africa	🟢	🟢	🟢	🟡	🟢	🔴	🟢	🔴	🟢	🟢
LeRoux 2010[189]	2008	South Africa	🟢	🟢	🔴	🟢	🟢	🟢	🟢	🟢	🔴	🟢
Nwadioha 2010[190]	2008	Nigeria	🟢	🟢	🟢	🟡	🟡	🟡	🟢	🟢	🟢	🔴
Nwadioha 2010[191]	2008	Nigeria	🟢	🟢	🔴	🟡	🟡	🟡	🟢	🟢	🟢	🔴
Djohan 2012[192]	2008	Côte d'Ivoire	🔴	🟢	🔴	🟢	🟡	🔴	🟢	🔴	🟢	🟢

Reference	Year[1]	Country	Q1	Q2	Q3	Q4	Q5	Q6	Q7	Q8	Q9	Q10
Vandenhoudt 2013[193]	2008	Kenya	🔴	🟢	🟢	🔴	🟡	🟢	🟢	🟢	🔴	🟢
Vandepitte 2012[194]	2009	Uganda	🔴	🟢	🟢	🟢	🟢	🟢	🟢	🔴	🟢	🔴
Vandepitte 2011[195]	2009	Uganda	🔴	🟢	🟢	🟢	🟡	🟢	🟢	🟢	🔴	🔴
Karou 2012[196]	2009	Burkina Faso	🟢	🟢	🔴	🟡	🟡	🔴	🟢	🟢	🟢	🔴
Tine 2019[197]	2009	Senegal	🟢	🟢	🔴	🟡	🟡	🟢	🔴	🔴	🟢	🟢
Nwadioha 2013[198]	2009	Nigeria	🔴	🟢	🔴	🟡	🟡	🔴	🟢	🔴	🟢	🟢
Okunlola 2009[199]	2009	Nigeria	🔴	🟢	🔴	🟡	🟢	🟢	🟢	🟢	🔴	🟢
De Jongh 2009[200]	2009*	South Africa	🟢	🟢	🔴	🟢	🟢	🟡	🟢	🟢	🔴	🟢
Djomand 2016[201]	2010	Kenya	🟢	🟢	🟢	🟢	🟡	🟢	🟢	🟢	🟢	🟢
Singa 2013[202]	2010	Kenya	🔴	🟢	🟢	🔴	🟢	🟢	🟢	🟢	🔴	🔴
De Jongh 2010[203]	2010*	South Africa	🔴	🟢	🔴	🟡	🟢	🟢	🟢	🟢	🟢	🟢
Makasa 2012[204]	2010	Zambia	🟢	🟢	🟢	🟢	🟢	🟢	🟢	🟢	🟢	🟢
Ibrahim 2014[205]	2011	Nigeria	🟢	🟢	🔴	🟢	🟢	🔴	🟢	🔴	🟢	🔴
Ibrahim 2013[206]	2011	Nigeria	🟢	🟢	🔴	🟢	🟢	🔴	🟢	🔴	🟢	🔴
Konate 2014[207]	2011	Côte d'Ivoire	🟢	🟢	🔴	🟡	🟡	🔴	🟢	🔴	🟢	🔴
Takuva 2014[208]	2011	Zimbabwe	🟢	🟢	🔴	🟡	🟢	🟢	🟢	🟢	🟢	🔴
Van der Eem 2016[209]	2012	South Africa	🟢	🟢	🟢	🟢	🟡	🟢	🟢	🟢	🔴	🟢
Mulu 2017[210]	2013	Ethiopia	🔴	🔴	🔴	🟢	🟡	🔴	🔴	🔴	🔴	🔴
Kerubo 2016[211]	2013	Kenya	🔴	🟢	🔴	🟢	🟡	🔴	🟢	🔴	🔴	🟢
Tine 2019[212]	2013	Senegal	🟢	🟢	🔴	🟡	🟡	🟢	🔴	🔴	🟢	🟢
NICD 2023[213]	2014	South Africa	🟢	🟢	🟢	🟢	🟢	🟢	🟢	🟢	🟢	🟢
Nkwabong 2015[214]	2014	Cameroon	🔴	🟢	🔴	🟡	🟡	🟢	🟢	🟢	🔴	🟢
Fentaw 2020[215]	2014	Ethiopia	🔴	🟢	🔴	🟡	🟡	🟢	🟢	🔴	🟢	🟢
Dela 2019[216]	2014	Ghana	🔴	🟢	🔴	🟡	🟡	🔴	🟢	🔴	🟢	🟢
Asmah 2018[217]	2014	Ghana	🔴	🟢	🔴	🔴	🟡	🔴	🔴	🔴	🔴	🟢
Rietmeijer 2019[218]	2015	Zimbabwe	🟢	🟢	🟢	🟢	🟢	🟢	🟢	🟢	🟢	🟢
Chirenje 2018[219]	2015	Zimbabwe	🟢	🟢	🟢	🟢	🟢	🟢	🟢	🟢	🟢	🟢
Kilmarx 2018[220]	2015	Zimbabwe	🟢	🟢	🟢	🟢	🟢	🟢	🟢	🟢	🟢	🔴
Mungati 2018[221]	2015	Zimbabwe	🟢	🟢	🟢	🟢	🟢	🟢	🟢	🟢	🟢	🟢
Rietmeijer 2018[222]	2015	Zimbabwe	🟢	🟢	🟢	🟢	🟢	🟢	🟢	🟢	🟢	🟢
Konadu 2019[223]	2015	Ghana	🔴	🟢	🔴	🔴	🟢	🟢	🟢	🔴	🟢	🔴
Odunvbun 2018[224]	2015	Nigeria	🔴	🟢	🔴	🟡	🟡	🔴	🟢	🔴	🔴	🔴
Oyeyipo 2015[225]	2015	Nigeria	🔴	🟢	🔴	🟡	🟢	🔴	🟢	🔴	🟢	🟢
Marcotte 2019[226]	2015	South Africa	🔴	🟢	🟢	🟡	🟡	🔴	🟢	🔴	🔴	🔴
Nabweyambo 2017[227]	2015	Uganda	🔴	🟢	🔴	🟢	🟡	🔴	🟢	🔴	🟢	🟢
Sakala 2016[228]	2015	Zambia	🔴	🔴	🟢	🟢	🟡	🟢	🟢	🔴	🔴	🔴
Van Liere 2019[229]	2016	South Africa	🔴	🟢	🟢	🔴	🟡	🔴	🟢	🟢	🔴	🟢
Latif 2018[230]	2016	Zimbabwe	🔴	🟢	🔴	🟡	🟢	🟢	🟢	🔴	🟢	🟢

Reference	Year[1]	Country	Q1	Q2	Q3	Q4	Q5	Q6	Q7	Q8	Q9	Q10
Mehta 2017[231]	2017*	Kenya	Green	Green	Green	Green	Yellow	Green	Green	Green	Red	Red
Gadoth 2020[232]	2017	Democratic Republic of Congo	Red	Green	Green	Red	Yellow	Red	Green	Red	Red	Red
Chen 2020[233]	2017	Malawi	Red	Green	Green	Green	Yellow	Green	Green	Red	Red	Green
Aduloju 2019[234]	2017	Nigeria	Red	Green	Red	Green	Yellow	Green	Red	Red	Green	Green
Kashibu 2018[235]	2017	Nigeria	Red	Green	Red	Green	Yellow	Green	Green	Red	Red	Green
Verwijs 2019[236]	2017	Rwanda	Red	Green	Green	Red	Yellow	Green	Green	Green	Green	Green
Chabata 2023[237]	2017	Zimbabwe	Red	Green	Green	Red	Green	Green	Green	Green	Red	Green
Nacht 2020[238]	2018	Kenya	Red	Green	Red	Red	Red	Green	Green	Red	Red	Red
Matoga 2023[239]	2018	Malawi	Red	Green	Green	Yellow	Yellow	Green	Green	Green	Red	Green
Wall 2021[240]	2018	Rwanda	Green	Green	Green	Red	Yellow	Green	Green	Red	Green	Green
Happel 2020[241]	2018	South Africa	Red	Green	Green	Green	Green	Red	Green	Green	Red	Green
Connolly 2020[242]	2018	Zambia	Red	Green	Green	Red	Yellow	Green	Green	Red	Green	Red
Pulei 2023[243]	2019	Kenya	Red	Green	Green	Yellow	Green	Green	Green	Red	Green	Green
Omosa-Manyonyi 2023[244]	2019	Kenya	Green	Green	Green	Yellow	Green	Green	Green	Green	Green	Green
Mabaso 2020[245]	2019	South Africa	Red	Green	Red	Yellow	Yellow	Red	Green	Red	Red	Green
Mabaso 2022[246]	2019	South Africa	Red	Green	Green	Yellow	Yellow	Red	Green	Red	Green	Red
Tshaka 2022[247]	2019	South Africa	Green	Red	Green	Yellow	Yellow	Green	Green	Green	Green	Green
Melendez 2022[248]	2020	Uganda	Green	Green	Green	Yellow	Green	Red	Green	Green	Green	Red
Beck 2023[249]	2020	Uganda	Red	Green	Green	Yellow	Yellow	Red	Green	Green	Red	Green
Zenebe 2021[250]	2020	Ethiopia	Red	Green	Red	Green	Yellow	Red	Green	Green	Red	Green
Ahmed 2022[251]	2021	Ethiopia	Red	Green	Red	Yellow	Yellow	Green	Green	Red	Red	Red
Chen 2023[252]	2021	Malawi	Green	Green	Green	Yellow	Yellow	Green	Green	Green	Red	Green
Sarenje 2022[253]	2021	Zambia	Red	Red	Green	Yellow	Yellow	Green	Green	Red	Green	Red

Q1: Was the study specifically designed to estimate the outcome/s of interest [*study design*]? **Q2:** Were criteria for inclusion in the sample clearly defined [*selection bias*]? **Q3:** Were study participants and their setting described in sufficient detail to determine representativeness of the target population [*selection bias*]? **Q4:** Were participants recruited in an appropriate way [*selection bias*]? **Q5:** Was the participation rate adequate [*selection bias*]? **Q6:** Were objective, standard criteria used to define participant symptoms [*measurement bias*]? **Q7**: Were the outcomes measured in a reliable and consistent way [*measurement bias*]? **Q8:** Were participants tested for multiple pathogens to prevent misclassification of infection outcomes [*measurement bias*]? **Q9:** Was the sample size sufficient to measure the outcome [*study precision*]? **Q10:** Was the reporting of the outcome(s) unambiguous [*reporting quality*]?

[1]Midpoint year of data collection. *Publication year used as a proxy for studies that did not report dates or year(s) of data collection.

Table P: Summary of critical appraisal criteria for included reports.

Category	Criteria	Vaginal discharge $N_{VD} = 87$		Urethral discharge $N_{UD} = 55$		Genital ulcer $N_{GU} = 80$	
		Yes	No or unclear	Yes	No or unclear	Yes	No or unclear
**Study design**	Q1	37 (42.5%)	50 (57.5%)	32 (58.2%)	23 (41.8%)	40 (50.0%)	40 (50.0%)
**Selection bias**	Q2	82 (94.3%)	5 (5.7%)	50 (90.9%)	5 (9.1%)	73 (91.2%)	7 (8.8%)
	Q3	36 (41.4%)	51 (58.6%)	21 (38.2%)	34 (61.8%)	56 (70.0%)	24 (30.0%)
	Q4	32 (36.8%)	55 (63.2%)	27 (49.1%)	28 (50.9%)	46 (57.5%)	34 (42.5%)
	Q5	34 (39.1%)	53 (60.9%)	21 (38.2%)	34 (61.8%)	40 (50.0%)	40 (50.0%)
**Measurement bias**	Q6	48 (55.2%)	39 (44.8%)	36 (65.5%)	19 (34.5%)	75 (93.8%)	5 (6.2%)
	Q7	81 (93.1%)	6 (6.9%)	47 (85.5%)	8 (14.5%)	75 (93.8%)	5 (6.2%)
	Q8	43 (49.4%)	44 (50.6%)	22 (40.0%)	33 (60.0%)	48 (60.0%)	32 (40.0%)
**Precision**	Q9	61 (70.1%)	26 (29.9%)	39 (70.9%)	16 (29.1%)	46 (57.5%)	34 (42.5%)
**Reporting quality**	Q10	51 (58.6%)	36 (41.4%)	37 (67.3%)	18 (32.7%)	52 (65.0%)	28 (35.0%)

N = number of studies.

Q1: Was the study specifically designed to estimate the outcome/s of interest? **Q2:** Were criteria for inclusion in the sample clearly defined? **Q3:** Were study participants and their setting described in sufficient detail to determine representativeness of the target population? **Q4:** Were participants recruited in an appropriate way? **Q5:** Was the participation rate adequate? **Q6:** Were objective, standard criteria used to define participant symptoms? **Q7**: Were the outcomes measured in a reliable and consistent way? **Q8:** Were participants tested for multiple pathogens to prevent misclassification of infection outcomes? **Q9:** Was the sample size sufficient to measure the outcome? **Q10:** Was the reporting of the outcome(s) unambiguous?

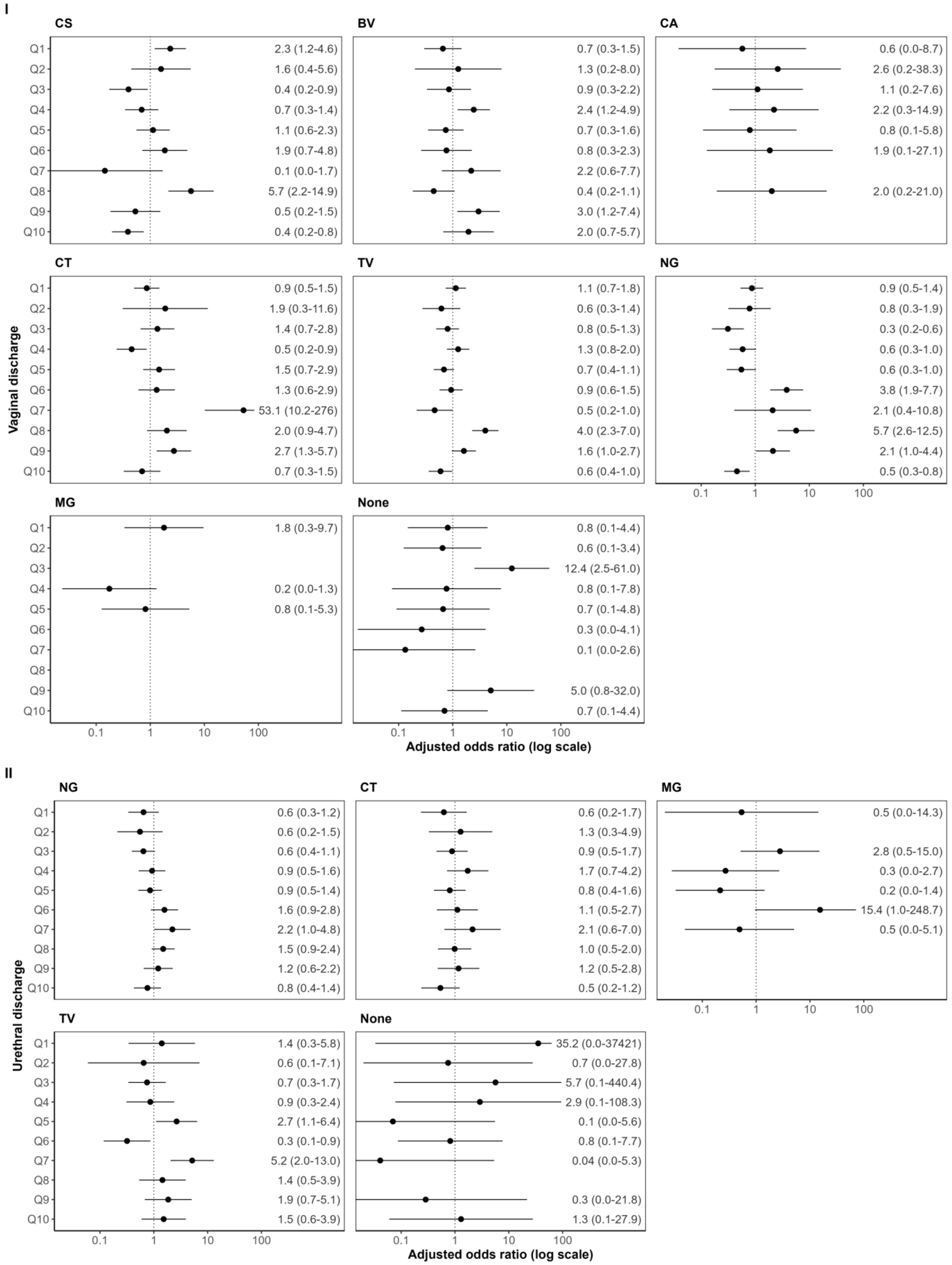

I
Vaginal discharge
CS
2.3 (1.2-4.6)
1.6 (0.4-5.6)
0.4 (0.2-0.9)
0.7 (0.3-1.4)
1.1 (0.6-2.3)
1.9 (0.7-4.8)
0.1 (0.0-1.7)
5.7 (2.2-14.9)
0.5 (0.2-1.5)
0.4 (0.2-0.8)
BV
0.7 (0.3-1.5)
1.3 (0.2-8.0)
0.9 (0.3-2.2)
2.4 (1.2-4.9)
0.7 (0.3-1.6)
0.8 (0.3-2.3)
2.2 (0.6-7.7)
0.4 (0.2-1.1)
3.0 (1.2-7.4)
2.0 (0.7-5.7)
CA
0.6 (0.0-8.7)
2.6 (0.2-38.3)
1.1 (0.2-7.6)
2.2 (0.3-14.9)
0.8 (0.1-5.8)
1.9 (0.1-27.1)
2.0 (0.2-21.0)
CT
0.9 (0.5-1.5)
1.9 (0.3-11.6)
1.4 (0.7-2.8)
0.5 (0.2-0.9)
1.5 (0.7-2.9)
1.3 (0.6-2.9)
53.1 (10.2-276)
2.0 (0.9-4.7)
2.7 (1.3-5.7)
0.7 (0.3-1.5)
TV
1.1 (0.7-1.8)
0.6 (0.3-1.4)
0.8 (0.5-1.3)
1.3 (0.8-2.0)
0.7 (0.4-1.1)
0.9 (0.6-1.5)
0.5 (0.2-1.0)
4.0 (2.3-7.0)
1.6 (1.0-2.7)
0.6 (0.4-1.0)
NG
0.9 (0.5-1.4)
0.8 (0.3-1.9)
0.3 (0.2-0.6)
0.6 (0.3-1.0)
0.6 (0.3-1.0)
3.8 (1.9-7.7)
2.1 (0.4-10.8)
5.7 (2.6-12.5)
2.1 (1.0-4.4)
0.5 (0.3-0.8)
MG
1.8 (0.3-9.7)
0.2 (0.0-1.3)
0.8 (0.1-5.3)
None
0.8 (0.1-4.4)
0.6 (0.1-3.4)
12.4 (2.5-61.0)
0.8 (0.1-7.8)
0.7 (0.1-4.8)
0.3 (0.0-4.1)
0.1 (0.0-2.6)
5.0 (0.8-32.0)
0.7 (0.1-4.4)
Q1 Q2 Q3 Q4 Q5 Q6 Q7 Q8 Q9 Q10
0.1 1 10 100
Adjusted odds ratio (log scale)
II
Urethral discharge
NG
0.6 (0.3-1.2)
0.6 (0.2-1.5)
0.6 (0.4-1.1)
0.9 (0.5-1.6)
0.9 (0.5-1.4)
1.6 (0.9-2.8)
2.2 (1.0-4.8)
1.5 (0.9-2.4)
1.2 (0.6-2.2)
0.8 (0.4-1.4)
CT
0.6 (0.2-1.7)
1.3 (0.3-4.9)
0.9 (0.5-1.7)
1.7 (0.7-4.2)
0.8 (0.4-1.6)
1.1 (0.5-2.7)
2.1 (0.6-7.0)
1.0 (0.5-2.0)
1.2 (0.5-2.8)
0.5 (0.2-1.2)
MG
0.5 (0.0-14.3)
2.8 (0.5-15.0)
0.3 (0.0-2.7)
0.2 (0.0-1.4)
15.4 (1.0-248.7)
0.5 (0.0-5.1)
TV
1.4 (0.3-5.8)
0.6 (0.1-7.1)
0.7 (0.3-1.7)
0.9 (0.3-2.4)
2.7 (1.1-6.4)
0.3 (0.1-0.9)
5.2 (2.0-13.0)
1.4 (0.5-3.9)
1.9 (0.7-5.1)
1.5 (0.6-3.9)
None
35.2 (0.0-37421)
0.7 (0.0-27.8)
5.7 (0.1-440.4)
2.9 (0.1-108.3)
0.1 (0.0-5.6)
0.8 (0.1-7.7)
0.04 (0.0-5.3)
0.3 (0.0-21.8)
1.3 (0.1-27.9)
Q1 Q2 Q3 Q4 Q5 Q6 Q7 Q8 Q9 Q10
0.1 1 10 100
Adjusted odds ratio (log scale)

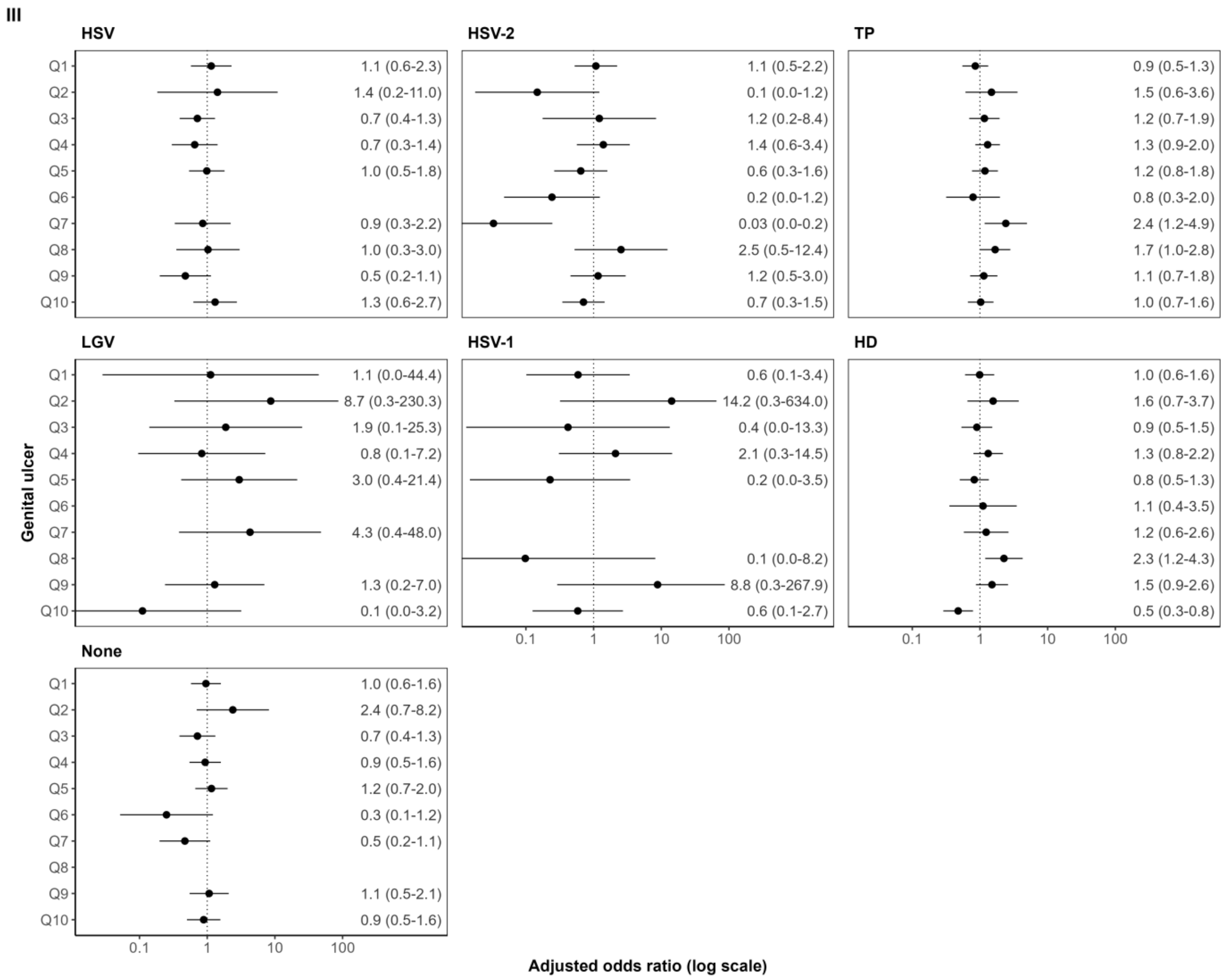

Fig C: Effect of critical appraisal criteria on the odds of diagnosis per pathogen, among symptomatic adults of mixed or unmeasured HIV status in sub-Saharan Africa.

Adjusted odds of diagnosis per pathogen among studies rated “no or unclear” compared to “yes” for each criterion in the critical appraisal tool, among adults symptomatic with (I) vaginal discharge, (II) urethral discharge, and (III) genital ulcer. Odds estimated using generalised linear mixed-effects model for each symptom. Points represent the adjusted odds of diagnosis among studies rated “no or unclear” compared to studies rated “yes”. Solid lines represent 95% confidence intervals. Red indicates statistical significance, whereas black indicates no statistical significance. Blank criteria signify that all studies were rated “yes”. Confidence intervals truncated by x-axis.

Q1: Was the study specifically designed to estimate the outcome/s of interest [*study design*]? **Q2:** Were the criteria for inclusion in the sample clearly defined [*selection bias*]? **Q3:** Were study participants and their setting described in sufficient detail to determine representativeness of the target population [*selection bias*]? **Q4:** Were study participants recruited in an appropriate way [*selection bias*]? **Q5:** Was the participation rate adequate [*selection bias*]? **Q6:** Were objective, standard criteria used to define participant symptoms [*measurement bias*]? **Q7**: Were the outcomes measured in a reliable and consistent way [*measurement bias*]? **Q8:** Were participants tested for multiple pathogens to prevent misclassification of infection outcomes [*measurement bias*]? **Q9:** Was the sample size sufficient to measure the outcome [*study precision*]? **Q10:** Was the reporting of the outcome(s) unambiguous [*reporting quality*]?

BV: Bacterial vaginosis, CA: *Candida albicans*, CS: *Candida* species (any), CT: *Chlamydia trachomatis*, HD: *Haemophilus ducreyi*, HSV: Herpes simplex virus (unspecified), HSV-1: Herpes simplex virus type 1, HSV-2: Herpes simplex virus type 2, LGV: lymphogranuloma venereum, MG: *Mycoplasma genitalium*, NG: *Neisseria gonorrhoeae*, TP: *Treponema pallidum*, TV: *Trichomonas vaginalis*, None: unknown aetiology.

Table Q: Estimated diagnosed proportion per pathogen in sub-Saharan Africa, with and without diagnostic test performance adjustment.

RTI	Adjusted proportion % (95% CI)		Unadjusted proportion % (95% CI)	
	1980	2015	1980	2015
**Vaginal discharge**				
CS	5.6 (1.5-18.2)	69.4 (44.3-86.6)	24.7 (14.5-38.9)	36.2 (23.9-50.6)
BV	45.5 (14.7-80.1)	50.0 (32.3-67.8)	24.7 (10.4-48.1)	37.6 (25.6-51.3)
CA	26.8 (7.6-62.1)	31.5 (12.4-59.9)	27.1 (12.8-48.6)	33.0 (16.4-55.3)
CT	5.3 (1.7-15.0)	16.2 (8.6-28.5)	4.0 (1.9-8.6)	13.1 (8.0-20.7)
TV	51.6 (32.3-70.5)	12.9 (7.8-20.7)	24.1 (15.2-36.1)	9.7 (6.5-14.2)
NG	10.6 (4.9-21.7)	6.6 (3.3-12.7)	7.0 (3.6-13.2)	5.9 (3.3-10.3)
MG	6.4 (0.2-65.2)	5.4 (1.8-14.8)	9.8 (1.0-53.4)	7.0 (3.1-15.4)
None	25.6 (6.5-63.1)	25.1 (10.7-48.4)	29.8 (12.9-54.8)	22.9 (11.9-39.5)
**Urethral discharge**				
NG	63.9 (47.1-77.8)	77.1 (68.1-84.1)	54.5 (40.9-67.5)	70.3 (61.6-77.7)
CT	10.6 (4.4-23.2)	21.9 (15.4-30.3)	8.3 (4.0-16.3)	19.1 (13.9-25.7)
MG	23.5 (7.0-55.4)	5.5 (3.7-8.3)	22.7 (8.3-49.1)	5.0 (3.4-7.4)
TV	29.3 (15.6-48.0)	2.8 (1.8-4.3)	12.5 (6.9-21.5)	3.9 (2.6-5.6)
None	38.9 (13.6-72.0)	10.0 (6.8-14.5)	39 (16.3-67.7)	9.5 (6.6-13.5)
**Genital ulcer**				
HSV	7.8 (4.3-13.7)	57.1 (40.9-71.9)	5.0 (2.6-9.5)	58.7 (41.1-74.4)
HSV-2	39.8 (19.4-64.4)	48.3 (32.9-64.1)	36.6 (16.1-63.4)	49.5 (32.6-66.5)
TP	17.9 (12.4-25.1)	9.3 (6.4-13.4)	16.2 (10.8-23.6)	7.8 (5.1-11.7)
LGV	24.6 (7.7-55.9)	2.3 (1.0-5.3)	47.4 (16.2-80.7)	1.2 (0.5-3.2)
HSV-1	1.8 (0.3-10.8)	2.0 (0.8-4.7)	2.6 (0.3-21.5)	0.6 (0.2-2.2)
HD	86.9 (79.7-91.8)	2.0 (1.2-3.3)	81.8 (71.8-88.9)	0.9 (0.5-1.7)
None	20.5 (12.9-30.9)	34.7 (25.5-45.1)	19.9 (12.0-31.2)	33.9 (24.2-45.3)

Estimated proportions are with or without diagnostic test performance adjustment and presented as percentages with 95% confidence intervals.

BV: Bacterial vaginosis, CA: *Candida albicans*, CS: *Candida* species (any), CT: *Chlamydia trachomatis*, HD: *Haemophilus ducreyi*, HSV: Herpes simplex virus (unspecified), HSV-1: Herpes simplex virus type 1, HSV-2: Herpes simplex virus type 2, LGV: lymphogranuloma venereum, MG: *Mycoplasma genitalium*, NG: *Neisseria gonorrhoeae*, TP: *Treponema pallidum*, TV: *Trichomonas vaginalis*. None: unknown aetiology.

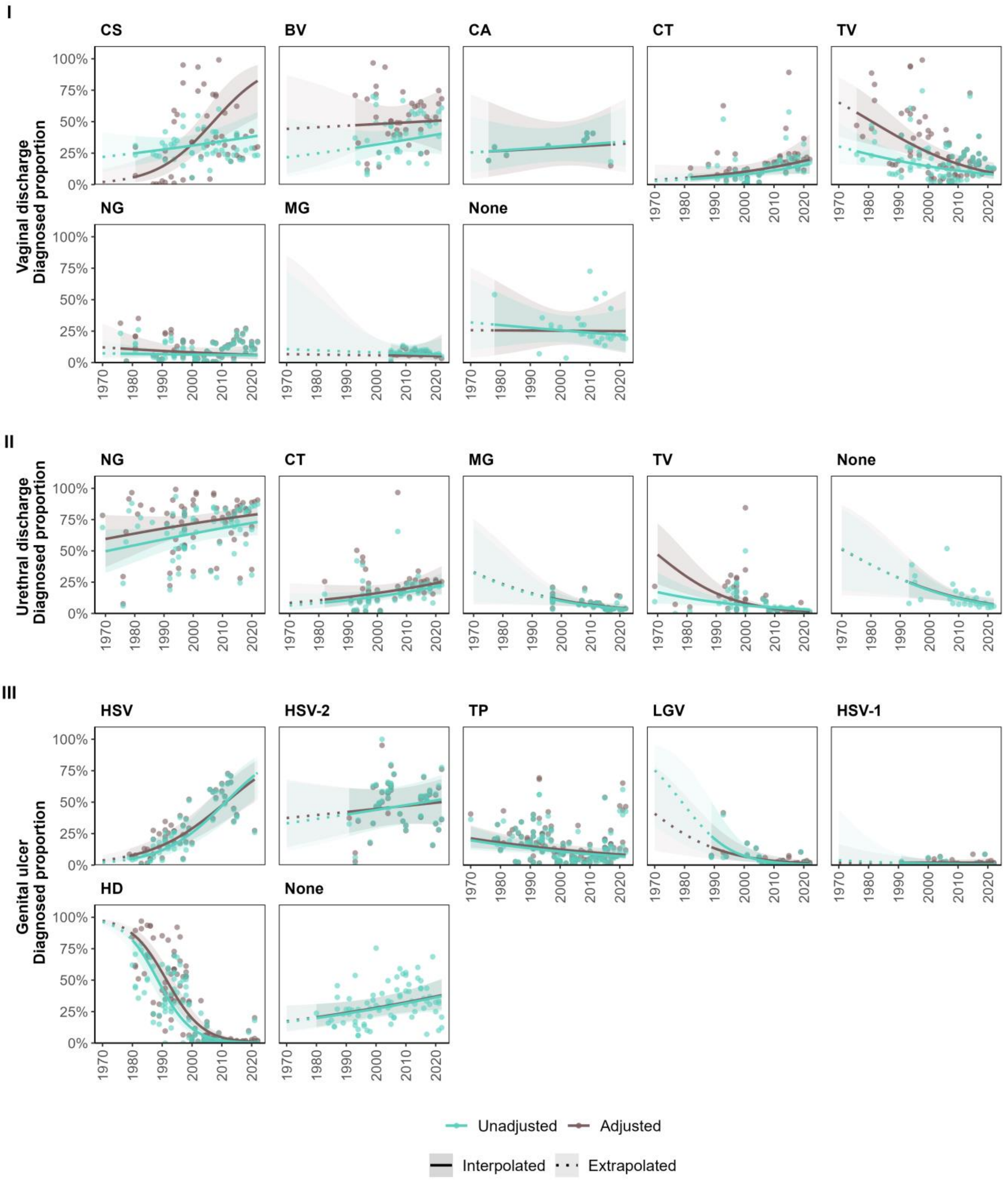

Fig D: Estimated diagnosed proportion per pathogen among symptomatic adults of mixed or unmeasured HIV status in sub-Saharan Africa over time, with and without diagnostic test performance adjustment.

Diagnosed proportion per pathogen among individuals symptomatic with (I) vaginal discharge, (II) urethral discharge, and (III) genital ulcer. Proportions estimated using generalised linear mixed-effects model for each symptom, using either observations as reported or adjusted for diagnostic test performance. Lines and shaded areas represent sex-matched population-weighted mean proportions and 95% confidence intervals. Solid lines and darker shading denote estimates and confidence intervals within the observed data range (interpolated), while dotted lines and lighter shading indicate estimates and confidence intervals beyond that time frame (extrapolated). Points represent study observations that are unadjusted or adjusted for test performance.

BV: Bacterial vaginosis, CA: *Candida albicans*, CS: *Candida* species (any), CT: *Chlamydia trachomatis*, HD: *Haemophilus ducreyi*, HSV: Herpes simplex virus (unspecified), HSV-1: Herpes simplex virus type 1, HSV-2: Herpes simplex virus type 2, LGV: lymphogranuloma venereum, MG: *Mycoplasma genitalium*, NG: *Neisseria gonorrhoeae*, TP: *Treponema pallidum*, TV: *Trichomonas vaginalis*, None: unknown aetiology.

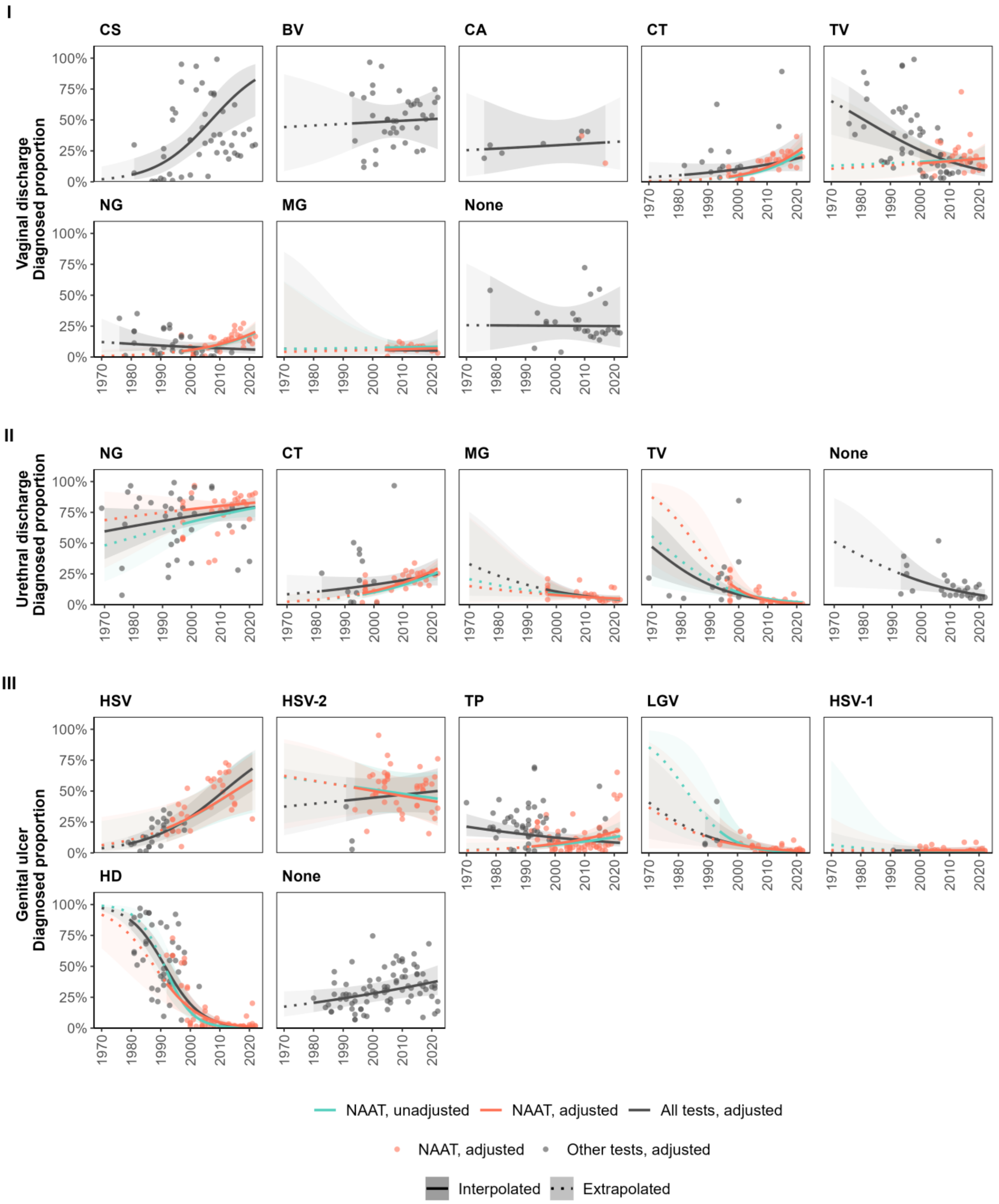

Fig E: Estimated diagnosed proportion per pathogen among symptomatic adults of mixed or unmeasured HIV status in sub-Saharan Africa over time, using NAAT with and without diagnostic test performance adjustment.

Diagnosed proportion per pathogen among individuals symptomatic with (I) vaginal discharge, (II) urethral discharge, and (III) genital ulcer. Proportions estimated using generalised linear mixed-effects model for each symptom, using observations attained using nucleic acid amplification testing (NAAT) as reported or adjusted for diagnostic test performance, compared to observations from all diagnostics adjusted for diagnostic test performance. Lines and shaded areas represent sex-matched population-weighted mean proportions and 95% confidence intervals. Solid lines and darker shading denote estimates and confidence intervals within the observed data range (interpolated), while dotted lines and lighter shading indicate estimates and confidence intervals beyond that time frame (extrapolated). Points represent study observations adjusted for diagnostic test performance and colour coded according to diagnostic test.

References

1 UN Statistics Division. Standard country or area codes for statistical use (M49). https://unstats.un.org/unsd/methodology/m49/ (accessed June 14, 2023).

2 Kufa T, Gumede L, Maseko D V, Radebe F, Kularatne R. The demographic and clinical profiles of women presenting with vaginal discharge syndrome at primary care facilities in South Africa: Associations with age and implications for management. *S Afr Med J* 2018; **108**: 876–80.

3 Kufa T, Radebe F, Cutler E, *et al.* Recency of HIV infection, antiretroviral therapy use and viral loads among symptomatic sexually transmitted infection service attendees in South Africa. *South African Med J* 2022; **112**: 96–101.

4 Lewis DA, Scott L, Slabbert M, *et al.* Escalation in the relative prevalence of ciprofloxacin-resistant gonorrhoea among men with urethral discharge in two South African cities: association with HIV seropositivity. *Sex Transm Infect* 2008; **84**: 352–5.

5 Müller EE, Gumede LYE, Maseko D V., *et al.* Emergence of high-level azithromycin-resistant Neisseria gonorrhoeae causing male urethritis in Johannesburg, South Africa, 2021. *Sex Health* 2023; **21**. DOI:10.1071/SH23143.

6 Mabugana MC, Dias BDC, Muller EE, *et al.* The evaluation of the Allplex™ BV molecular assay for the diagnosis of bacterial vaginosis in symptomatic South African females. *Diagn Microbiol Infect Dis* 2023; **106**: 115924.

7 Kularatne RS, Muller EE, Maseko D V, Kufa-Chakezha T, Lewis DA. Trends in the relative prevalence of genital ulcer disease pathogens and association with HIV infection in Johannesburg, South Africa, 2007-2015. *PLoS One* 2018; **13**: e0194125.

8 Kularatne R, Maseko V, Mahlangu P, Muller E, Kufa T. Etiological surveillance of male urethritis syndrome in South Africa: 2019 to 2020. *Sex Transm Dis* 2022; **49**: 560–4.

9 Kularatne R, Muller E, Maseko V, Dias BDC, Kufa T. Etiological surveillance of vaginal discharge syndrome in South Africa: 2019 to 2020. *Sex Transm Dis* 2022; **49**: 565–70.

10 Kularatne R, Venter JME, Maseko V, Muller E, Kufa T. Etiological surveillance of genital ulcer syndrome in South Africa: 2019 to 2020. *Sex Transm Dis* 2022; **49**: 571–5.

11 Mhlongo S, Magooa P, Müller EE, *et al.* Etiology and STI/HIV coinfections among patients with urethral and vaginal discharge syndromes in South Africa. *Sex Transm Dis* 2010; **37**: 566–70.

12 Mathebula RC, Kuonza LR, Musekiwa A, Kularatne R, Maseko V, Kufa T. Factors associated with repeat genital symptoms among sexually transmitted infection service attendees in South Africa, 2015 - 2016. *S Afr Med J* 2020; **110**: 661–6.

13 Lewis DA, Muller E, Steele L, *et al.* Prevalence and associations of genital ulcer and urethral pathogens in men presenting with genital ulcer syndrome to primary health care clinics in South Africa. *Sex Transm Dis* 2012; **39**: 880–5.

14 Lewis DA, Marsh K, Radebe F, Maseko V, Hughes G. Trends and associations of Trichomonas vaginalis infection in men and women with genital discharge syndromes in Johannesburg, South Africa. *Sex Transm Infect* 2013; **89**: 523–7.

15 Fearon E, Chabata ST, Magutshwa S, *et al.* Estimating the population size of female sex workers in Zimbabwe: Comparison of estimates obtained using different methods in twenty sites and development of a national-level estimate. 2020.

16 Stannah J, Soni N, Keng J, *et al.* Trends in HIV testing, the treatment cascade, and HIV incidence among men who have sex with men in Africa: a systematic review and meta-analysis. *Lancet HIV* 2023; **10**: E528–42.

17 World Health Organization. Laboratory tests for the detection of reproductive tract infections. Geneva, 1999.

18 World Health Organization. Prevalence and incidence of selected sexually transmitted infections: Methods and results used by WHO to generate 2005 estimates. Geneva, 2011.

19 Kaydos-Daniels SC, Miller WC, Hoffman I, *et al.* The use of specimens from various genitourinary sites in men to detect Trichomonas vaginalis infection. *J Infect Dis* 2004; **189**: 1926–31.

20 Hobbs MM, Lapple DM, Lawing LF, *et al.* Methods for detection of Trichomonas vaginalis in the male partners of infected women: implications for control of trichomoniasis. *J Clin Microbiol* 2006; **44**: 3994–9.

21 World Health Organization. Laboratory and point-of-care diagnostic testing for sexually transmitted infections, including HIV. Geneva, 2023.

22 Rogan WJ, Gladen B. Estimating prevalence from the results of a screening test. *Am J Epidemiol* 1978; **107**: 71–6.

23 Flor M, Weiß M, Selhorst T, Müller-Graf C, Greiner M. Comparison of Bayesian and frequentist methods for prevalence estimation under misclassification. *BMC Public Health* 2020; **20**: 1135.

24 Speybroeck N, Devleesschauwer B, Joseph L, Berkvens D. Misclassification errors in prevalence estimation: Bayesian handling with care. *Int J Public Health* 2013; **58**: 791–5.

25 Dadhwal V, Hariprasad R, Mittal S, Kapil A. Prevalence of bacterial vaginosis in pregnant women and predictive value of clinical diagnosis. *Arch Gynecol Obstet* 2010; **281**: 101–4.

26 Thomason JL, Anderson RJ, Gelbart SM, *et al.* Simplified Gram stain interpretive method for diagnosis of bacterial vaginosis. *Am J Obstet Gynecol* 1992; **167**: 16–9.

27 Schwebke JR, Hillier SL, Sobel JD, McGregor JA, Sweet RL. Validity of the vaginal gram stain for the diagnosis of bacterial vaginosis. *Obstet Gynecol* 1996; **88**: 573–6.

28 Hoppe JE, Frey P. Evaluation of six commercial tests and the germ-tube test for presumptive identification of Candida albicans. *Eur J Clin Microbiol Infect Dis* 1999; **18**: 188–91.

29 Gaydos CA, Beqaj S, Schwebke JR, *et al.* Clinical validation of a test for the diagnosis of vaginitis. *Obstet Gynecol* 2017; **130**: 181–9.

30 Chatwani AJ, Mehta R, Hassan S, Rahimi S, Jeronis S, Dandolu V. Rapid testing for vaginal yeast detection: a prospective study. *Obs Gynecol* 2007; **196**: 309–10.

31 Schwebke JR, Gaydos CA, Nyirjesy P, Paradis S, Kodsi S, Cooper CK. Diagnostic performance of a molecular test versus clinician assessment of vaginitis. *J Clin Microbiol* 2018; **56**: e00252-18.

32 Grillo-Ardila CF, Torres M, Gaitán HG. Rapid point of care test for detecting urogenital Chlamydia trachomatis infection in nonpregnant women and men at reproductive age. *Cochrane database Syst Rev* 2020; **1**.

33 Gaydos CA, Manhart LE, Taylor SN, *et al.* Molecular testing for mycoplasma genitalium in the United States: Results from the AMES prospective multicenter clinical study. *J Clin Microbiol* 2019; **57**.

34 Gaydos CA, Klausner JD, Pai NP, Kelly H, Coltart C, Peeling RW. Rapid and point-of-care tests for the diagnosis of Trichomonas vaginalis in women and men. *Sex Transm Infect* 2017; **93**: S31–5.

35 Paz-Bailey G, Rahman M, Chen C, *et al.* Changes in the etiology of sexually transmitted diseases in Botswana between 1993 and 2002: implications for the clinical management of genital ulcer disease. *Clin Infect Dis* 2005; **41**: 1304–12.

36 Legoff J, Péré H, Bélec L. Diagnosis of genital herpes simplex virus infection in the clinical laboratory. *Virol J* 2014; **11**.

37 Arshad Z, Alturkistani A, Brindley D, Lam C, Foley K, Meinert E. Tools for the diagnosis of herpes simplex virus 1/2: Systematic review of studies published between 2012 and 2018. *JMIR Public Heal Surveill* 2019; **5**.

38 Diagnostic Hybrids. D3 DFA Chlamydiae Culture Confirmation Kit. REF: 01-040000. Athens, Ohio, 2008.

39 Touati A, Laurier-Nadalié C, Bébéar C, Peuchant O, de Barbeyrac B. Evaluation of four commercial real-time PCR assays for the detection of lymphogranuloma venereum in Chlamydia trachomatis-positive anorectal samples. *Clin Microbiol Infect* 2021; **27**: 909.e1-909.e5.

40 Peeling RW, Ye H. Diagnostic tools for preventing and managing maternal and congenital syphilis: An overview. *Bull World Health Organ* 2004; **82**: 439–46.

41 Hambie EA, Larsen SA, Perryman MW, Pettit DE, Mullally RL, Whittington W. Comparison of a new rapid plasma reagin card test with the standard rapid plasma reagin 18-mm circle card test and the venereal disease research laboratory slide test for serodiagnosis of syphilis. *J Clin Microbiol* 1983; **17**: 249–54.

42 Perryman MW, Larsen SA, Hambie EA, Pettit DE, Mullally RL, Whittington W. Evaluation of a new rapid plasma reagin card test as a screening test for syphilis. *J Clin Microbiol* 1982; **16**: 286–90.

43 Parham CE, Pettit DE, Larsen SA, Hambie EA, Perryman MW, McGrew BE. Interlaboratory comparison of the toluidine red unheated serum test antigen preparation. *J Clin Microbiol* 1984; **20**: 434–7.

44 Cantor AG, Pappas M, Daeges M, Nelson HD. Screening for Syphilis: Updated Evidence Report and Systematic Review for the US Preventive Services Task Force. *JAMA* 2016; **315**: 2328–37.

45 Sena AC, White BL, Sparling PF. Novel Treponema pallidum serologic tests: A paradigm shift in syphilis screening for the 21st century. *Clin Infect Dis* 2010; **51**: 700–8.

46 Park IU, Fakile YF, Chow JM, *et al.* Performance of treponemal tests for the diagnosis of syphilis. *Clin Infect Dis* 2019; **68**: 913–8.

47 Munn Z, Moola S, Lisy K, Riitano D, Tufanaru C. Chapter 5: Systematic reviews of prevalence and incidence. In: Aromataris E, Munn Z, eds. JBI Manual for Evidence Synthesis. JBI, 2020.

48 Natural Earth - Free vector and raster map data. https://www.naturalearthdata.com/ (accessed April 2, 2024).

49 Arya OP. Some highlights on the aetiology of the non gonococcal urethral discharge in males in Kampala, Uganda. *East Afr Med J* 1972; **49**: 817–24.

50 Ongom VL, Lwanga VN, Mugisha JK, Mafigiri JT. Social background to venereal disease at Kasangati. *East Afr Med J* 1971; **48**: 367–71.

51 Van Binsbergen JJ, Schulpen TWJ, Patel SC, Van Binsbergen-Ingelse A. Urethral or vaginal discharge at a rural hospital in Kenya. *East Afr Med J* 1980; **57**: 540–4.

52 Fakunle YM, Watkins B. Influence of self-medication on prevalence and antibiotic sensitivity of N. gonorrhoeae in Zaria (Nigeria). *East Afr Med J* 1976; **53**: 693–6.

53 Ballard RC, Schoub BD, Schneider J, Robins-Browne RM, Koornhof HJ. Urethritis in white men - a microbiological appraisal. *South African Med J* 1977; **51**: 702–6.

54 Rubin A, Russell JM, Mauff A. Efficacy of econazole in the treatment of candidiasis and other vaginal discharges. *South African Med J* 1980; **57**: 407–8.

55 Meheus A, Van Dyck E, Friedman F. Genital infections in Swaziland. *Ann Soc Belg Med Trop (1920)* 1982; **62**: 361–7.

56 Meheus A, Van Dyck E, Ursi JP, Ballard RC, Piot P. Etiology of genital ulcerations in Swaziland. *Sex Transm Dis* 1983; **10**: 33–5.

57 Ursi JP, van Dyck E, van Houtte C, *et al.* Syphilis in Swaziland: a serological survey. *Br J Vener Dis* 1981; **57**: 95–9.

58 Meheus A, Ballard R, Dlamini M, Ursi JP, Van Dyck E, Piot P. Epidemiology and aetiology of urethritis in Swaziland. *Int J Epidemiol* 1980; **9**: 239–45.

59 Nsanze H, Fast M V, D'Costa LJ, Tukei P, Curran J, Ronald A. Genital ulcers in Kenya. Clinical and laboratory study. *Br J Vener Dis* 1981; **57**: 378–81.

60 Fast M V, Nsanze H, Plummer FA, D'Costa LJ, MacLean IW, Ronald AR. Treatment of chancroid. A comparison of sulphamethoxazole and trimethoprim-sulphamethoxazole. *Br J Vener Dis* 1983; **59**: 320–4.

61 Yvert F, Riou JY, Frost E, Ivanoff B, Ossari S, Bouatsia P. [Gonococcal infections in Gabon (Haut-Ogooue)]. *Pathol Biol (Paris)* 1984; **32**: 80–4.

62 Hoosen AA, Ross SM, Mulla MJ, Patel M. The incidence of selected vaginal infections among pregnant urban Blacks. *South African Med J* 1981; **59**: 827–9.

63 Crewe-Brown HH. Genital ulceration in males at Ga-Rankuwa Hospital, Pretoria. *South African Med J* 1982; **62**.

64 D'Costa LJ, Plummer FA, Bowmer I, *et al.* Prostitutes are a major reservoir of sexually transmitted diseases in Nairobi, Kenya. *Sex Transm Dis* 1985; **12**: 64–7.

65 Mirza NB, Nsanze H, D'Costa LJ, Piot P. Microbiology of vaginal discharge in Nairobi, Kenya. *Br J Vener Dis* 1983; **59**: 186–8.

66 Mabey DC, Whittle HC. Genital and neonatal chlamydial infection in a trachoma endemic area. *Lancet* 1982; **2**: 300–1.

67 Mabey DC, Lloyd-Evans NE, Conteh S, Forsey T. Sexually transmitted diseases among randomly selected attenders at an antenatal clinic in The Gambia. *Br J Vener Dis* 1984; **60**: 331–6.

68 Plummer FA, Nsanze H, D'Costa LJ, *et al.* Single-dose therapy of chancroid with trimethoprim-sulfametrole. *N Engl J Med* 1983; **309**: 67–71.

69 Mauff AC, Ballard RC, Bilgeri YR, Koornhof HJ. Isolation of Haemophilus ducreyi from genital ulcerations in white men in Johannesburg. *South African Med J* 1983; **63**: 236–7.

70 Coovadia YM, Kharsany A, Hoosen A. The microbial aetiology of genital ulcers in black men in Durban, South Africa. *Genitourin Med* 1985; **61**: 266–9.

71 Greenblatt RM, Lukehart SA, Plummer FA, *et al.* Genital ulceration as a risk factor for human immunodeficiency virus infection. *AIDS* 1988; **2**: 47–50.

72 Plummer FA, D'Costa LJ, Nsanze H, *et al.* Clinical and microbiologic studies of genital ulcers in Kenyan women. *Sex Transm Dis* 1985; **12**: 193–7.

73 Kreiss JK, Koech D, Plummer FA, *et al.* AIDS virus infection in Nairobi prostitutes: spread of the epidemic to East Africa. *N Engl J Med* 1986; **314**: 414–8.

74 Dangor Y, Fehler G, Exposto FD, Koornhof HJ. Causes and treatment of sexually acquired genital ulceration in southern Africa. *South African Med J* 1989; **76**: 339–41.

75 Ismail SO, Ahmed HJ, Grillner L, Hederstedt B, Issa A, Bygdeman SM. Sexually transmitted diseases in men in Mogadishu, Somalia. *Int J STD AIDS* 1990; **1**: 102–6.

76 Meiring JA, Kemp E, Jennings DL, Mdhlovu M, Koornhof HJ. High-level penicillin-resistant gonococcal infections in Port Elizabeth. *South African Med J* 1989; **75**: 118–9.

77 Mabey DCW, Wall RA, Bello CSS. Aetiology of genital ulceration in the Gambia. *Genitourin Med* 1987; **63**: 312–5.

78 Leclerc A, Frost E, Collet M, Goeman J, Bedjabaga L. Urogenital Chlamydia trachomatis in Gabon: an

unrecognised epidemic. *Genitourin Med* 1988; **64**: 308–11.

79 Bogaerts J, Kestens L, van Dyck E, Tello WM, Akingeneye J, Mukantabana V. Genital ulcers in a primary health clinic in Rwanda: impact of HIV infection on diagnosis and ulcer healing (1986-1992). *Int J STD AIDS* 1998; **9**: 706–10.

80 Faye-Kette YH, Kouassi AA, Sylla-Koko DF, *et al.* [Prevalence of 4 agents of sexually transmitted diseases in leukorrhea in Abidjan (Ivory Coast)]. *Bull la Société Pathol Exot* 1993; **86**: 245–7.

81 O'Farrell N, Hoosen AA, Coetzee KD, van den Ende J. Genital ulcer disease: accuracy of clinical diagnosis and strategies to improve control in Durban, South Africa. *Genitourin Med* 1994; **70**: 7–11.

82 Daly CC, Maggwa N, Mati JK, *et al.* Risk factors for gonorrhoea, syphilis, and trichomonas infections among women attending family planning clinics in Nairobi, Kenya. *Genitourin Med* 1994; **70**: 155–61.

83 Thior I, Diouf G, Diaw IK, *et al.* Sexually transmitted diseases and risk of HIV infection in men attending a sexually transmitted diseases clinic in Dakar, Senegal. *Afr J Reprod Health* 1997; **1**: 26–35.

84 Ndinya-Achola JO, Kihara AN, Fisher LD, *et al.* Presumptive specific clinical diagnosis of genital ulcer disease (GUD) in a primary health care setting in Nairobi. *Int J STD AIDS* 1996; **7**: 201–5.

85 Kamya MR, Nsubuga P, Grant RM, Hellman N. The high prevalence of genital herpes among patients with genital ulcer disease in Uganda. *Sex Transm Dis* 1995; **22**: 351–4.

86 Hanson S, Sunkutu RM, Kamanga J, Hojer B, Sandstrom E. STD care in Zambia: an evaluation of the guidelines for case management through a syndromic approach. *Int J STD AIDS* 1996; **7**: 324–32.

87 Le Bacq F, Mason PR, Gwanzura L, Robertson VJ, Latif AS. HIV and other sexually transmitted diseases at a rural hospital in Zimbabwe. *Genitourin Med* 1993; **69**: 352–6.

88 Kimani J, Bwayo JJ, Anzala AO, *et al.* Low dose erythromycin regimen for the treatment of chancroid. *East Afr Med J* 1995; **72**: 645–8.

89 Oni AA, Adu FD, Ekweozor CC, Bakare RA. Herpetic urethritis in male patients in Ibadan. *West Afr J Med* 1997; **16**: 27–9.

90 Totten PA, Kuypers JM, Chen CY, *et al.* Etiology of genital ulcer disease in Dakar, Senegal, and comparison of PCR and serologic assays for detection of Haemophilus ducreyi. *J Clin Microbiol* 2000; **38**: 268–73.

91 Htun Y, Radebe F, Fehler HG, Ballard RC. Changes in the patterns of sexually transmitted infection among South African mineworkers, associated with the emergence of the HIV/AIDS epidemic. *South African Med J* 2007; **97**: 1155–60.

92 Grosskurth H, Mayaud P, Mosha F, *et al.* Asymptomatic gonorrhoea and chlamydial infection in rural Tanzanian men. *Br Med J* 1996; **312**: 277–80.

93 Tswana SA, Nystrom L, Moyo SR, *et al.* Hospital-based study of sexually transmitted diseases at Murewa Rural district hospital, Zimbabwe 1991-1992. *Sex Transm Dis* 1995; **22**: 1–6.

94 Ghys PD, Diallo MO, Ettiegne-Traore V, *et al.* Genital ulcers associated with human immunodeficiency virus-related immunosuppression in female sex workers in Abidjan, Ivory Coast. *J Infect Dis* 1995; **172**: 1371–4.

95 Meless H, Abegaze B. Drug susceptibility of Neisseria isolates from patients attending clinics for sexually transmitted diseases in Addis Ababa. *East Afr Med J* 1995; **74**: 447–9.

96 Harms G, Matull R, Randrianasolo D, *et al.* Pattern of sexually transmitted diseases in a Malagasy population. *Sex Transm Dis* 1994; **21**: 315–20.

97 Dallabetta G, Behets F, Lule G, *et al.* Specificity of dysuria and discharge complaints and presence of urethritis in male patients attending an STD clinic in Malawi. *Sex Transm Infect* 1998; **74**: S34-7.

98 Behets FM, Liomba G, Lule G, *et al.* Sexually transmitted diseases and human immunodeficiency virus control in Malawi: a field study of genital ulcer disease. *J Infect Dis* 1995; **171**: 451–5.

99 Cossa HA, Gloyd S, Vaz RG, *et al.* Syphilis and HIV infection among displaced pregnant women in rural Mozambique. *Int J STD AIDS* 1994; **5**: 117–23.

100 Alary M, Baganizi E, Guedeme A, *et al.* Evaluation of clinical algorithms for the diagnosis of gonococcal and chlamydial infections among men with urethral discharge or dysuria and women with vaginal discharge in Benin. *Sex Transm Infect* 1998; **74**: S44–9.

101 Thomas T, Choudhri S, Kariuki C, Moses S. Identifying cervical infection among pregnant women in Nairobi, Kenya: limitations of risk assessment and symptom-based approaches. *Genitourin Med* 1996; **72**: 334–8.

102 King R, Choudhri SH, Nasio J, *et al.* Clinical and in situ cellular responses to Haemophilus ducreyi in the presence or absence of HIV infection. *Int J STD AIDS* 1998; **9**: 531–6.

103 Htun Y, Morse SA, Dangor Y, *et al.* Comparison of clinically directed, disease specific, and syndromic protocols for the management of genital ulcer disease in Lesotho. *Sex Transm Infect* 1998; **74**: S23-8.

104 Morse SA, Trees DL, Htun Y, *et al.* Comparison of clinical diagnosis and standard laboratory and molecular

methods for the diagnosis of genital ulcer disease in Lesotho: association with human immunodeficiency virus infection. *J Infect Dis* 1997; **175**: 583–9.

105 Lo BB, Philippon M, Cunin P, Meynard D, TandiaDiagana M. Sexually transmitted diseases agents of genital discharges in Nouakchott, Mauritania. *Bull la Société Pathol Exot* 1997; **90**: 81–2.

106 Chen CY, Ballard RC, Beck-Sague CM, *et al.* Human immunodeficiency virus infection and genital ulcer disease in South Africa: the herpetic connection. *Sex Transm Dis* 2000; **27**: 21–9.

107 Schneider H, Coetzee DJ, Fehler HG, *et al.* Screening for sexually transmitted diseases in rural South African women. *Sex Transm Infect* 1998; **74**: S147-52.

108 Pillay DG, Hoosen AA, Vezi B, Moodley C. Diagnosis of Trichomonas vaginalis in male urethritis. *Trop Geogr Med* 1994; **46**: 44–5.

109 Mayaud P, Msuya W, Todd J, *et al.* STD rapid assessment in Rwandan refugee camps in Tanzania. *Genitourin Med* 1997; **73**: 33–8.

110 Aseffa A, Ishak A, Stevens R, *et al.* Prevalence of HIV, syphilis and genital chlamydial infection among women in north-west Ethiopia. *Epidemiol Infect* 1998; **120**: 171–7.

111 MacDonald KS, Malonza I, Chen DK, *et al.* Vitamin A and risk of HIV-1 seroconversion among Kenyan men with genital ulcers. *AIDS* 2001; **15**: 635–9.

112 Jackson DJ, Rakwar JP, Chohan B, *et al.* Urethral infection in a workplace population of East African men: evaluation of strategies for screening and management. *J Infect Dis* 1997; **175**: 833–8.

113 Gateau T, Zeller HG. Epidemiological approach for sexually transmitted diseases in Antsiranana (north Madagascar). Between prevention and treatment, the choice of a strategy against sexually transmitted diseases. *Arch Inst Pasteur Madagascar* 1996; **63**: 8–11.

114 Bakare RA, Oni AA, Umar US, *et al.* Non-gonococcal urethritis due to Chlamydia trachomatis: the Ibadan experience. *Afr J Med Med Sci* 2002; **31**: 17–20.

115 Ramuthaga TN, Mahomed FM, Greeff AS, Crewe-Brown HH, Vermeulen R. Comparison of urine with urethral swabs for the detection of Chlamydia trachomatis in men attending an STD clinic. *South African Med J* 1995; **85**: 1287–9.

116 Ahmed HJ, Borrelli S, Jonasson J, *et al.* Monoclonal antibodies against Haemophilus ducreyi lipooligosaccharide and their diagnostic usefulness. *Eur J Clin Microbiol Infect Dis* 1995; **14**: 892–8.

117 Latif AS, Mason PR, Marowa E, Gwanzura L, Chingono A, Mbengeranwa OL. Risk factors for gonococcal and chlamydial cervical infection in pregnant and non-pregnant women in Zimbabwe. *Cent Afr J Med* 1999; **45**: 252–8.

118 Farina C, Malighetti V, Lombart JP, *et al.* Yeasts from vaginal exudates in the Comoros islands. *J Mycol Med* 2000; **10**: 91–3.

119 Malonza IM, Tyndall MW, Ndinya-Achola JO, *et al.* A randomized, double-blind, placebo-controlled trial of single-dose ciprofloxacin versus erythromycin for the treatment of chancroid in Nairobi, Kenya. *J Infect Dis* 1999; **180**: 1886–93.

120 Cohen MS, Hoffman IF, Royce RA, *et al.* Reduction of concentration of HIV-1 in semen after treatment of urethritis: implications for prevention of sexual transmission of HIV-1. AIDSCAP Malawi Research Group. *Lancet* 1997; **349**: 1868–73.

121 Dieye AM, Samb NGD, Ba A, *et al.* Effectiveness of syndromic approach for management of uretral discharge in Senegal. *Med Trop* 2003; **63**: 45–8.

122 Lai W, Chen CY, Morse SA, *et al.* Increasing relative prevalence of HSV-2 infection among men with genital ulcers from a mining community in South Africa. *Sex Transm Infect* 2003; **79**: 202–7.

123 Ballard RC, Ye H, Matta A, Dangor Y, Radebe F. Treatment of chancroid with azithromycin. *Int J STD AIDS* 1996; **7**: S9-12.

124 Pepin J, Sobela F, Deslandes S, *et al.* Etiology of urethral discharge in West Africa: the role of Mycoplasma genitalium and Trichomonas vaginalis. *Bull World Health Organ* 2001; **79**: 118–26.

125 Morency P, Dubois MJ, Gresenguet G, *et al.* Aetiology of urethral discharge in Bangui, Central African Republic. *Sex Transm Infect* 2001; **77**: 125–9.

126 Gomes JP, Tavira L, Exposto F, Prieto E, Catry MA. Neisseria gonorrhoeae and Chlamydia trachomatis infections in patients attending STD and family planning clinics in Bissau, Guinea-Bissau. *Acta Trop* 2001; **80**: 261–4.

127 Fonck K, Mwai C, Rakwar J, Kirui P, Ndinya-Achola JO, Temmerman M. Healthcare-seeking behavior and sexual behavior of patients with sexually transmitted diseases in Nairobi, Kenya. *Sex Transm Dis* 2001; **28**: 367–71.

128 Ballard RC, Koornhof HJ, Chen CY, Radebe F, Fehler HG, Htun Y. The influence of concomitant HIV infection

on the serological diagnosis of primary syphilis in southern Africa. *South African Med J* 2007; **97**: 1151–4.

129 Behets FM, Andriamiadana J, Randrianasolo D, *et al.* Chancroid, primary syphilis, genital herpes, and lymphogranuloma venereum in Antananarivo, Madagascar. *J Infect Dis* 1999; **180**: 1382–5.

130 Rajagopal M, Hoosen AA, Moodley J, Kharsany ABM, Moodley P, Sturm AW. Analysis of genital tract infections at a dedicated sexually transmitted diseases clinic. *South African J Epidemiol Infect* 1999; **14**: 77–82.

131 Hoosen AA, Moodley J, Maitin P, Sturm AW. Bacterial vaginosis in symptomatic women attending a gynaecology outpatient clinic. *South African J Epidemiol Infect* 1997; **12**: 119–21.

132 Ramjee G, Karim SS, Sturm AW. Sexually transmitted infections among sex workers in KwaZulu-Natal, South Africa. *Sex Transm Dis* 1998; **25**: 346–9.

133 Nzila N, Laga M, Thiam MA, *et al.* HIV and other sexually transmitted diseases among female prostitutes in Kinshasa. *AIDS* 1991; **5**: 715–21.

134 Anorlu RI, Fagbenro Beyioku AF, Fagorala T, Abudu OO, Galadanci HS. Prevalence of trichomonas vaginalis in patients with vaginal discharge in Lagos, Nigeria. *Niger Postgrad Median J* 2001; **8**: 183–6.

135 Ballard RC, Fehler HG, Htun Y, Radebe F, Jensen JS, Taylor-Robinson D. Coexistence of urethritis with genital ulcer disease in South Africa: influence on provision of syndromic management. *Sex Transm Infect* 2002; **78**: 274–7.

136 Obasi AI, Balira R, Todd J, *et al.* Prevalence of HIV and Chlamydia trachomatis infection in 15--19-year olds in rural Tanzania. *Trop Med Int Heal* 2001; **6**: 517–25.

137 Msuya SE, Mbizvo EM, Stray-Pedersen B, Sundby J, Sam NE, Hussain A. Risk assessment at the primary health care level in Moshi, Tanzania: limits in predicting sexually transmitted infections among women. *Cent Afr J Med* 2006; **52**: 97–104.

138 Msuya SE, Mbizvo E, Stray-Pedersen B, Sundby J, Sam NE, Hussain A. Reproductive tract infections among women attending primary health care facilities in Moshi, Tanzania. *East Afr Med J* 2002; **79**: 16–21.

139 Hoyo C, Hoffman I, Moser BK, *et al.* Improving the accuracy of syndromic diagnosis of genital ulcer disease in Malawi. *Sex Transm Dis* 2005; **32**: 231–7.

140 Chalamilla G, Mbwana J, Mhalu F, *et al.* Patterns of sexually transmitted infections in adolescents and youth in Dar es Salaam, Tanzania. *BMC Infect Dis* 2006; **6**: 22.

141 Ahmed HJ, Mbwana J, Gunnarsson E, *et al.* Etiology of genital ulcer disease and association with human immunodeficiency virus infection in two tanzanian cities. *Sex Transm Dis* 2003; **30**: 114–9.

142 Mbizvo ME, Musya SE, Stray-Pedersen B, Chirenje Z, Hussain A. Bacterial vaginosis and intravaginal practices: association with HIV. *Cent African Med J* 2004; **50**: 41–6.

143 Vuylsteke BL, Ettiegne-Traore V, Anoma CK, *et al.* Assessment of the validity of and adherence to sexually transmitted infection algorithms at a female sex worker clinic in Abidjan, Cote d'Ivoire. *Sex Transm Dis* 2003; **30**: 284–91.

144 Price MA, Miller WC, Kaydos-Daniels SC, *et al.* Trichomoniasis in men and HIV infection: data from 2 outpatient clinics at Lilongwe Central Hospital, Malawi. *J Infect Dis* 2004; **190**: 1448–55.

145 Anorlu R, Imosemi D, Odunukwe N, Abudu O, Otuonye M. Prevalence of HIV among Women with Vaginal Discharge in a Gynecological Clinic. *J Natl Med Assoc* 2004; **96**: 367–71.

146 Bakare RA, Oni AA, Umar US, Kehinde AO, Fayemiwo SA, Fasina NA. Ureaplasma urealyticum as a cause of non-gonococcal urethritis: the Ibadan experience. *Niger Postgrad Med J* 2002; **9**: 140–5.

147 Moodley P, Wilkinson D, Connolly C, Moodley J, Sturm AW. Trichomonas vaginalis is associated with pelvic inflammatory disease in women infected with human immunodeficiency virus. *Clin Infect Dis* 2002; **34**: 519–22.

148 Nilsen A, Kasubi MJ, Mohn SC, Mwakagile D, Langeland N, Haarr L. Herpes simplex virus infection and genital ulcer disease among patients with sexually transmitted infections in Dar es Salaam, Tanzania. *Acta Derm Venereol* 2007; **87**: 355–9.

149 Riedner G, Todd J, Rusizoka M, *et al.* Possible reasons for an increase in the proportion of genital ulcers due to herpes simplex virus from a cohort of female bar workers in Tanzania. *Sex Transm Infect* 2007; **83**: 91–6.

150 Watson-Jones D, Mugeye K, Mayaud P, *et al.* High prevalence of trichomoniasis in rural men in Mwanza, Tanzania: results from a population based study. *Sex Transm Infect* 2000; **76**: 355–62.

151 Pickering JM, Whitworth JA, Hughes P, *et al.* Aetiology of sexually transmitted infections and response to syndromic treatment in southwest Uganda. *Sex Transm Infect* 2005; **81**: 488–93.

152 Mason PR, Gwanzura L, Gregson S, Katzenstein DA. Chlamydia trachomatis in symptomatic and asymptomatic men: detection in urine by enzyme immunoassay. *Cent Afr J Med* 2000; **46**: 62–5.

153 Wolday D, Z GM, Mohammed Z, *et al.* Risk factors associated with failure of syndromic treatment of sexually transmitted diseases among women seeking primary care in Addis Ababa. *Sex Transm Infect* 2004; **80**: 392–4.

154 Pepin J, Deslandes S, Khonde N, *et al.* Low prevalence of cervical infections in women with vaginal discharge in west Africa: implications for syndromic management. *Sex Transm Infect* 2004; **80**: 230–5.

155 Romoren M, Velauthapillai M, Rahman M, Sundby J, Klouman E, Hjortdahl P. Trichomoniasis and bacterial vaginosis in pregnancy: inadequately managed with the syndromic approach. *Bull World Heal Organ* 2007; **85**: 297–304.

156 Bankole H, Faye-Kette H, Laruche G, Dabis F, Welffens-Ekra C, Dosso M. [Cell culture for active Chlamydia trachomatis infection in a population of symptomatic women in Abidjan]. *Bull la Société Pathol Exot* 2001; **94**: 235–8.

157 Tadesse A, Mekonnen A, Kassu A, Asmelash T. Antimicrobial sensitivity of Neisseria gonorrhoeae in Gondar, Ethiopia. *East Afr Med J* 2001; **78**: 259–61.

158 Zachariah R, Harries AD, Nkhoma W, *et al.* Behavioural characteristics, prevalence of Chlamydia trachomatis and antibiotic susceptibility of Neisseria gonorrhoeae in men with urethral discharge in Thyolo, Malawi. *Trans R Soc Trop Med Hyg* 2002; **96**: 232–5.

159 Ly F, Gueye N, Samb ND, Sow PS, Ndiaye B, Mahe A. [Prospective study of sexually transmitted infections in Dakar, Senegal]. *Médecine Trop* 2006; **66**: 64–8.

160 Moodley P, Sturm PDJ, Vanmali T, Wilkinson D, Connolly C, Sturm AW. Association between HIV-1 infection, the etiology of genital ulcer disease, and response to syndromic management. *Sex Transm Dis* 2003; **30**: 241–5.

161 Otuonye NM, Odunukwe NN, Idigbe EO, *et al.* Aetiological agents of vaginitis in Nigerian women. *Br J Biomed Sci* 2004; **61**: 175–8.

162 Namkinga LA, Matee MI, Kivaisi AK, Moshiro C. Prevalence and risk factors for vaginal candidiasis among women seeking primary care for genital infections in Dar es Salaam, Tanzania. *East Afr Med J* 2005; **82**: 138–43.

163 Mwansasu A, Mwakagile D, Haarr L, Langeland N. Detection of HSV-2 in genital ulcers from STD patients in Dar es Salaam, Tanzania. *J Clin Virol* 2002; **24**: 183–92.

164 Rassjo EB, Kambugu F, Tumwesigye MN, Tenywa T, Darj E. Prevalence of sexually transmitted infections among adolescents in Kampala, Uganda, and theoretical models for improving syndromic management. *J Adolesc Heal* 2006; **38**: 213–21.

165 Fayemiwo SA, Odaibo GN, Oni AA, Ajayi AA, Bakare RA, Olaleye DO. Genital ulcer diseases among HIV-infected female commercial sex workers in Ibadan, Nigeria. *Afr J Med Med Sci* 2011; **40**: 39–46.

166 Msuya SE, Uriyo J, Stray-Pedersen B, Sam NE, Mbizvo EM. The effectiveness of a syndromic approach in managing vaginal infections among pregnant women in northern Tanzania. *East Afr J Public Health* 2009; **6**: 263–7.

167 O'Farrell N, Morison L, Moodley P, *et al.* Genital ulcers and concomitant complaints in men attending a sexually transmitted infections clinic: implications for sexually transmitted infections management. *Sex Transm Dis* 2008; **35**: 545–9.

168 Weiss HA, Paz Bailey G, Phiri S, *et al.* Episodic therapy for genital herpes in sub-saharan Africa: a pooled analysis from three randomized controlled trials. *PLoS One* 2011; **6**: e22601.

169 LeGoff J, Mayaud P, Gresenguet G, *et al.* Performance of HerpeSelect and Kalon assays in detection of antibodies to herpes simplex virus type 2. *J Clin Microbiol* 2008; **46**: 1914–8.

170 Abauleth R, Boni S, Kouassi-Mbengue A, Konan J, Deza S. [Causation and treatment of infectious leucorrhoea at the Cocody University Hospital (Abidjan, Cote d'Ivoire)]. *Sante* 2006; **16**: 191–5.

171 Mehta SD, Moses S, Parker CB, Agot K, Maclean I, Bailey RC. Circumcision status and incident herpes simplex virus type 2 infection, genital ulcer disease, and HIV infection. *AIDS* 2012; **26**: 1141–9.

172 Onyekonwu CL, Olumide YM, Oresanya FA, Onyekonwu GC. Vaginal discharge: aetiological agents and evaluation of syndromic management in Lagos. *Niger J Med* 2011; **20**: 155–62.

173 Suntoke TR, Hardick A, Tobian AA, *et al.* Evaluation of multiplex real-time PCR for detection of Haemophilus ducreyi, Treponema pallidum, herpes simplex virus type 1 and 2 in the diagnosis of genital ulcer disease in the Rakai District, Uganda. *Sex Transm Infect* 2009; **85**: 97–101.

174 Pepin J, Sobela F, Khonde N, *et al.* The syndromic management of vaginal discharge using single-dose treatments: a randomized controlled trial in West Africa. *Bull World Heal Organ* 2006; **84**: 729–38.

175 Pepin J, Deslandes S, Giroux G, *et al.* The complex vaginal flora of West African women with bacterial vaginosis. *PLoS One* 2011; **6**: e25082.

176 Phiri S, Zadrozny S, Weiss HA, *et al.* Etiology of genital ulcer disease and association with HIV infection in Malawi. *Sex Transm Dis* 2013; **40**: 923–8.

177 Brankin AE, Tobian AA, Laeyendecker O, *et al.* Aetiology of genital ulcer disease in female partners of male

participants in a circumcision trial in Uganda. *Int J STD AIDS* 2009; **20**: 650–1.

178 Zimba TF, Apalata T, Sturm WA, Moodley P. Aetiology of sexually transmitted infections in Maputo, Mozambique. *J Infect Dev Ctries* 2011; **5**: 41–7.

179 Paz-Bailey G, Sternberg M, Puren AJ, Steele L, Lewis DA. Determinants of HIV type 1 shedding from genital ulcers among men in South Africa. *Clin Infect Dis* 2010; **50**: 1060–7.

180 Paz-Bailey G, Sternberg M, Puren AJ, *et al.* Improvement in healing and reduction in HIV shedding with episodic acyclovir therapy as part of syndromic management among men: a randomized, controlled trial. *J Infect Dis* 2009; **200**: 1039–49.

181 Ojuromi OT, Oyibo WA, Tayo AO, *et al.* Reliance on microscopy in Trichomonas vaginalis diagnosis and its prevalence in females presenting with vaginal discharge in Lagos, Nigeria. *J Infect Dev Ctries* 2007; **1**: 210–3.

182 Nwadioha SI, Bako IA, Onwuezobe I, Egah DZ. Vaginal trichomoniasis among HIV patients attending primary health care centers of Jos, Nigeria. *Asian Pacific J Trop Dis* 2012; **2**: 337–41.

183 Nwadioha S, Egah D, Banwat E, Egesie J, Onwuezobe I. Prevalence of bacterial vaginosis and its risk factors in HIV/AIDS patients with abnormal vaginal discharge. *Asian Pacific J Trop Dis* 2011; **4**: 156–8.

184 Bochner AF, Baeten JM, Rustagi AS, *et al.* A cross-sectional analysis of Trichomonas vaginalis infection among heterosexual HIV-1 serodiscordant African couples. *Sex Transm Infect* 2017; **93**: 520–9.

185 Brown LB, Krysiak R, Kamanga G, *et al.* Neisseria gonorrhoeae antimicrobial susceptibility in Lilongwe, Malawi, 2007. *Sex Transm Dis* 2010; **37**: 169–72.

186 Nwadioha S, Egah D, Nwokedi E, Onwuezobe I. A study of female genital swabs in primary health care centres in Jos, Nigeria. *Asian Pacific J Trop Dis* 2011; **1**: 52–4.

187 Muvunyi CM, Hernandez TC. Prevalence of bacterial vaginosis in women with vaginal symptoms in South Province, Rwanda. *African J Clin Exp Microbiol* 2009; **10**.

188 Moodley P, Wilkinson D, Connolly C, Sturm AW. Influence of HIV-1 coinfection on effective management of abnormal vaginal discharge. *Sex Transm Dis* 2003; **30**: 1–5.

189 Le Roux MC, Ramoncha MR, Adam A, Hoosen AA. Aetiological agents of urethritis in symptomatic South African men attending a family practice. *Int J STD AIDS* 2010; **21**: 477–81.

190 Nwadioha S, Egesie JO, Emejuo H, Iheanacho E. A study of female genital swabs in a Nigerian Tertiary Hospital. *Asian Pac J Trop Med* 2010; **3**: 577–9.

191 Nwadioha S, Egesie JO, Emejuo H, Iheanacho E. Prevalence of pathogens of abnormal vaginal discharges in a Nigerian tertiary hospital. *Asian Pac J Trop Med* 2010; **3**: 483–5.

192 Djohan V, Angora KE, Vanga-Bosson AH, *et al.* [In vitro susceptibility of vaginal Candida albicans to antifungal drugs in Abidjan (Ivory Coast)]. *J Mycol Med* 2012; **22**: 129–33.

193 Vandenhoudt HM, Langat L, Menten J, *et al.* Prevalence of HIV and other sexually transmitted infections among female sex workers in Kisumu, Western Kenya, 1997 and 2008. *PLoS One* 2013; **8**: e54953.

194 Vandepitte J, Bukenya J, Hughes P, *et al.* Clinical characteristics associated with Mycoplasma genitalium infection among women at high risk of HIV and other STI in Uganda. *Sex Transm Dis* 2012; **39**: 487–91.

195 Vandepitte J, Bukenya J, Weiss HA, *et al.* HIV and other sexually transmitted infections in a cohort of women involved in high-risk sexual behavior in Kampala, Uganda. *Sex Transm Dis* 2011; **38**: 316–23.

196 Karou SD, Djigma F, Sagna T, *et al.* Antimicrobial resistance of abnormal vaginal discharges microorganisms in Ouagadougou, Burkina Faso. *Asian Pac J Trop Biomed* 2012; **2**: 294–7.

197 Tine RC, Sylla K, Ka R, *et al.* A Study of Trichomonas vaginalis infection and correlates in women with vaginal discharge referred at fann teaching hospital in Senegal. *J Parasitol Res* 2019; : 1–8.

198 Nwadioha SI, Nwokedi EO, Egesie J, Enejuo H. Vaginal candidiasis and its risk factors among women attending a Nigerian teaching hospital. *Niger Postgrad Med J* 2013; **20**: 20–3.

199 Okunlola MA, Stella-Maris ON, Tokzaka AA, Ojengbede Oladosu A. Study on Vaginitis Among Intrauterine Contraceptive Device Users in Ibadan, South-western Nigeria. *J Reprod Contracept* 2009; **20**: 247–55.

200 De Jongh M, Le Roux M, Adam A, Caliendo AM, Hoosen AA. Co-Infection with Neisseria gonorrhoeae, Chlamydia trachomatis and Trichomonas vaginalis in Symptomatic South African Men with Urethritis: Implications for Syndromic Management. *Open Trop Med J* 2009; **2**: 13–6.

201 Djomand G, Gao H, Singa B, *et al.* Genital infections and syndromic diagnosis among HIV-infected women in HIV care programmes in Kenya. *Int J STD AIDS* 2016; **27**: 19–24.

202 Singa B, Glick SN, Bock N, *et al.* Sexually transmitted infections among HIV-infected adults in HIV care programs in Kenya: a national sample of HIV clinics. *Sex Transm Dis* 2013; **40**: 148–53.

203 De Jongh M, Lekalakala MR, Le Roux M, Hoosen AA. Risk of having a sexually transmitted infection in women presenting at a termination of pregnancy clinic in Pretoria, South Africa. *J Obstet Gynaecol (Lahore)* 2010; **30**:

480–3.

204 Makasa M, Buve A, Sandøy IF. Etiologic pattern of genital ulcers in Lusaka, Zambia: Has chancroid been eliminated? *Sex Transm Dis* 2012; **39**: 787–91.

205 Ibrahim SM, Bukar M, Galadima GB, Audu BM, Ibrahim HA. Prevalence of bacterial vaginosis in pregnant women in Maiduguri, North-Eastern Nigeria. *Niger J Clin Pract* 2014; **17**: 154–8.

206 Ibrahim SM, Mohammed B, Yahaya M, Audu BM, Ibrahim HA. Prevalence of vaginal candidiasis among pregnant women with abnormal vaginal discharge in Maiduguri. *Niger J Med* 2013; **22**: 138–42.

207 Konate A, Yavo W, Kassi FK, *et al.* Aetiologies and contributing factors of vulvovaginal candidiasis in Abidjan (Cote d'Ivoire). *J Mycol Med* 2014; **24**: 93–9.

208 Takuva S, Mugurungi O, Mutsvangwa J, *et al.* Etiology and antimicrobial susceptibility of pathogens responsible for urethral discharge among men in Harare, Zimbabwe. *Sex Transm Dis* 2014; **41**: 713–7.

209 van der Eem L, Dubbink JH, Struthers HE, *et al.* Evaluation of syndromic management guidelines for treatment of sexually transmitted infections in South African women. *Trop Med Int Heal* 2016; **21**: 1138–46.

210 Mulu W, Abera B, Yimer M, Hailu T, Ayele H, Abate D. Bacterial agents and antibiotic resistance profiles of infections from different sites that occurred among patients at Debre Markos Referral Hospital, Ethiopia: a cross-sectional study. *BMC Res Notes* 2017; **10**: 254.

211 Kerubo E, Laserson KF, Otecko N, *et al.* Prevalence of reproductive tract infections and the predictive value of girls' symptom-based reporting: findings from a cross-sectional survey in rural western Kenya. *Sex Transm Infect* 2016; **92**: 251–6.

212 Tine RC, Dia L, Sylla K, Sow D, Lelo S, Ndour CT. Trichomonas vaginalis and Mycoplasma infections among women with vaginal discharge at Fann teaching hospital in Senegal. *Trop Parasitol* 2019; **9**: 45–53.

213 National Institute for Communicable Diseases. Database of aetiological surveillance of vaginal discharge, urethral discharge and genital ulcer syndromes in South Africa. 2023.

214 Nkwabong E, Dingom MAN. Acute pelvic inflammatory disease in Cameroon: a cross sectional descriptive study. *Afr J Reprod Health* 2015; **19**: 87–91.

215 Fentaw S, Abubeker R, Asamene N, Assefa M, Bekele Y, Tigabu E. Antimicrobial susceptibility profile of Gonococcal isolates obtained from men presenting with urethral discharge in Addis Ababa, Ethiopia: Implications for national syndromic treatment guideline. *PLoS One* 2020; **15**: e0233753.

216 Dela H, Attram N, Behene E, *et al.* Risk factors associated with gonorrhea and chlamydia transmission in selected health facilities in Ghana. *BMC Infect Dis* 2019; **19**: 425.

217 Asmah RH, Agyeman RO, Obeng-Nkrumah N, *et al.* Trichomonas vaginalis infection and the diagnostic significance of detection tests among Ghanaian outpatients. *BMC Womens Heal* 2018; **18**: 206.

218 Rietmeijer CA, Mungati M, Kilmarx PH, *et al.* Serological markers for syphilis among persons presenting with syndromes associated with sexually transmitted infections: Results From the Zimbabwe STI etiology study. *Sex Transm Dis* 2019; **46**: 579–83.

219 Chirenje ZM, Dhibi N, Handsfield HH, *et al.* The etiology of vaginal discharge syndrome in Zimbabwe: Results from the Zimbabwe STI etiology study. *Sex Transm Dis* 2018; **45**: 422–8.

220 Kilmarx PH, Gonese E, Lewis DA, *et al.* HIV infection in patients with sexually transmitted infections in Zimbabwe: Results from the Zimbabwe STI etiology study. *PLoS One* 2018; **13**: e0198683.

221 Mungati M, Machiha A, Mugurungi O, *et al.* The etiology of genital ulcer disease and coinfections with Chlamydia trachomatis and Neisseria gonorrhoeae in Zimbabwe: Results from the Zimbabwe STI etiology study. *Sex Transm Dis* 2018; **45**: 61–8.

222 Rietmeijer CA, Mungati M, MacHiha A, *et al.* The etiology of male urethral discharge in Zimbabwe: Results from the Zimbabwe STI Etiology Study. *Sex Transm Dis* 2018; **45**: 56–60.

223 Konadu DG, Owusu-Ofori A, Yidana Z, *et al.* Prevalence of vulvovaginal candidiasis, bacterial vaginosis and trichomoniasis in pregnant women attending antenatal clinic in the middle belt of Ghana. *BMC Pregnancy Childbirth* 2019; **19**: 341.

224 Odunvbun WO. Prevalence of chlamydia infection using chlamydia antigen detection test and it's predictive value in tubal disease among infertile women attending the gynaecological clinic of the Eku Baptist Hospital, Eku, Delta State, South-South Nigeria. *Ann Biomed Sci* 2018; **17**.

225 Oyeyipo OO, Onasoga MF. Incidence and speciation of Candida species among non-gravid young females in Ilorin, North Central, Nigeria. *J Appl Sci Environ Manag* 2015; **19**: 680–5.

226 Marcotte H, Larsson PG, Andersen KK, *et al.* An exploratory pilot study evaluating the supplementation of standard antibiotic therapy with probiotic lactobacilli in south African women with bacterial vaginosis. *BMC Infect Dis* 2019; **19**: 824.

227 Nabweyambo S, Kakaire O, Sowinski S, *et al.* Very low sensitivity of wet mount microscopy compared to PCR

against culture in the diagnosis of vaginal trichomoniasis in Uganda: a cross sectional study. *BMC Res Notes* 2017; **10**: 259.

228 Sakala J, Chizuni N, Nzala S. A study on usefulness of a set of known risk factors in predicting maternal syphilis infections in three districts of Western Province, Zambia. *Pan Afr Med J* 2016; **24**: 75.

229 van Liere G, Kock MM, Radebe O, *et al.* High rate of repeat sexually transmitted diseases among men who have sex with men in South Africa: a prospective cohort study. *Sex Transm Dis* 2019; **46**: e105–7.

230 Latif AS, Gwanzura L, Machiha A, *et al.* Antimicrobial susceptibility in Neisseria gonorrhoeae isolates from five sentinel surveillance sites in Zimbabwe, 2015-2016. *Sex Transm Infect* 2018; **94**: 62–6.

231 Mehta SD, Pradhan AK, Green SJ, *et al.* Microbial Diversity of Genital Ulcers of HSV-2 Seropositive Women. *Sci Rep* 2017; **7**: 15475.

232 Gadoth A, Shannon CL, Hoff NA, *et al.* Prenatal chlamydial, gonococcal, and trichomonal screening in the Democratic Republic of Congo for case detection and management. *Int J STD AIDS* 2020; **31**: 221–9.

233 Chen JS, Matoga M, Khan S, *et al.* Estimating syphilis seroprevalence among patients in a sexually transmitted infections clinic in Lilongwe, Malawi. *Int J STD AIDS* 2020; **31**: 359–63.

234 Aduloju OP, Akintayo AA, Aduloju T. Prevalence of bacterial vaginosis in pregnancy in a tertiary health institution, south western Nigeria. *Pan Afr Med J* 2019; **33**: 9.

235 Kashibu E, Victor O, Ojumah I, Akafa R, Rikwentishe E. *Trichomonas vaginalis* infection: Prevalence and risk factors among ante-natal attendees in a tertiary facility in Taraba State, north-east Nigeria. *Niger J Parasitol* 2018; **39**: 199–203.

236 Verwijs MC, Agaba SK, Sumanyi JC, *et al.* Targeted point-of-care testing compared with syndromic management of urogenital infections in women (WISH): a cross-sectional screening and diagnostic accuracy study. *Lancet Infect Dis* 2019; **19**: 658–69.

237 Chabata S, Fearon E, Musemburi S, *et al.* Sexually transmitted infections among female sex workers in Zimbabwe - high and rising prevalenec blocks progress towards global elimination targets. *Unpublished* 2023.

238 Nacht C, Agingu W, Otieno F, Odhiambo F, Mehta SD. Antimicrobial resistance patterns in Neisseria gonorrhoeae among male clients of a sexually transmitted infections clinic in Kisumu, Kenya. *Int J STD AIDS* 2020; **31**: 46–52.

239 Matoga M, Chen JS, Massa C, *et al.* HIV and urethritis: time required for antiretroviral therapy to suppress HIV in semen. *AIDS* 2023; **37**: 2233–8.

240 Wall KM, Nyombayire J, Parker R, *et al.* Etiologies of genital inflammation and ulceration in symptomatic Rwandan men and women responding to radio promotions of free screening and treatment services. *PLoS One* 2021; **16**: e0250044.

241 Happel AU, Singh R, Mitchev N, *et al.* Testing the regulatory framework in South Africa - a single-blind randomized pilot trial of commercial probiotic supplementation to standard therapy in women with bacterial vaginosis. *BMC Infect Dis* 2020; **20**: 491.

242 Connolly S, Wall KM, Parker R, *et al.* Sociodemographic factors and STIs associated with Chlamydia trachomatis and Neisseria gonorrhoeae infections in Zambian female sex workers and single mothers. *Int J STD AIDS* 2020; **31**: 364–74.

243 Pulei AN, Lokken EM, Kinuthia J, *et al.* Derivation and internal validation of a risk score for predicting chlamydia trachomatis infection in Kenyan women planning conception. *Sex Transm Dis* 2023; **50**: 625–33.

244 Omosa-Manyonyi GS, de Kam M, Tostmann A, *et al.* Evaluation and optimization of the syndromic management of female genital tract infections in Nairobi, Kenya. *BMC Infect Dis* 2023; **23**: 1–14.

245 Mabaso N, Naicker C, Nyirenda M, Abbai N. Prevalence and risk factors for Trichomonas vaginalis infection in pregnant women in South Africa. *Int J STD AIDS* 2020; **31**: 351–8.

246 Mabaso N, Ngobese B, Tinarwo P, Abbai N. Prevalence of Chlamydia trachomatis infection in pregnant women from Durban, South Africa. *Int J STD AIDS* 2022; **33**: 920–927.

247 Tshaka TR, Singh R, Apalata TR, Mbulawa ZZA. Aetiology of genital ulcer disease and associated factors among Mthatha public clinic attendees. *South African J Infect Dis* 2022; **37**: 1–9.

248 Melendez JH, Hardick J, Onzia A, *et al.* Retrospective analysis of Ugandan men with urethritis reveals Mycoplasma genitalium and associated macrolide resistance. *Microbiol Spectr* 2022; **10**: e0230421.

249 Beck EJ, Laeyendecker O, Saraf S, *et al.* High level of HIV viral suppression in a cross-sectional study of Ugandan men with urethritis and bacterial STI. *Int J STD AIDS* 2023; **34**: 998–1003.

250 Zenebe MH, Mekonnen Z, Loha E, Padalko E. Prevalence, risk factors and association with delivery outcome of curable sexually transmitted infections among pregnant women in Southern Ethiopia. *PLoS One* 2021; **16**: e0248958.

251 Ahmed M, Admassu Ayana D, Abate D. Bacterial Vaginosis and associated factors among pregnant women

attending antenatal care in Harar City, eastern Ethiopia. *Infect Drug Resist* 2022; **15**: 3077–86.

252 Chen JS, Matoga MM, Gaither CF, *et al.* Dramatic shift in the etiology of genital ulcer disease among patients visiting a sexually transmitted infections clinic in Lilongwe, Malawi. *Sex Transm Dis* 2023; **50**: 753–9.

253 Sarenje KL, Ngalamika O, Maimbolwa MC, Siame A, Munsaka SM, Kwenda G. Antimicrobial resistance of Neisseria gonorrhoeae isolated from patients attending sexually transmitted infection clinics in Urban Hospitals, Lusaka, Zambia. *BMC Infect Dis* 2022; **22**: 1–7.
