# Supplementary material for: Aetiology of vaginal discharge, urethral discharge, and genital ulcer in sub-Saharan Africa: A systematic review and meta-regression
Source: PLoS Med. 2024 May 20;21(5):e1004385. doi: 10.1371/journal.pmed.1004385 (PMC11104670; doi:10.1371/journal.pmed.1004385)
Supplement: Attachment — Submitted filename: JMichalow_Response to reviewers.pdf [file pmed.1004385.s004.pdf]

Response to reviewers

Requests from the editors:

1. Per PLOS Medicine policy, we ask that you update your literature to within 6 months of the present time (minimally 6 months before submission);

Response: The literature search has been updated up to 20 December 2023. We identified 8 additional peer-reviewed publications, which were then included in the analysis. The manuscript has therefore been updated throughout to reflect the additional data (abstract, results, discussion, figures, tables, and supplementary material). The findings of our study, and consequent conclusions, are largely unchanged.

2. At this stage, we ask that you include a short, non-technical Author Summary of your research to make findings accessible to a wide audience that includes both scientists and non-scientists. The Author Summary should immediately follow the Abstract in your revised manuscript. This text is subject to editorial change and should be distinct from the scientific abstract. Ideally each sub-heading should contain 2-3 single sentence, concise bullet points containing the most salient points from your study. In the final bullet point of 'What Do These Findings Mean?' Please include the main limitations of the study in non-technical language. Please see our author guidelines for more information: https://journals.plos.org/plosmedicine/s/revising-your-manuscript#loc-author-summary.

Response: The Author Summary has been added (page 3):

Why was this study done?

- *Syndromic case management is a common approach for treating sexually transmitted infections in sub-Saharan Africa.*
- *Characterising the infectious aetiologies (causes) of each syndrome is crucial to ensure adequate choice of treatment.*
- *There is a lack of recent comprehensive assessments on the aetiologies for vaginal discharge, urethral discharge, and genital ulcer in sub-Saharan Africa.*

What did the researchers do and find?

- *We conducted a systematic review that included 190 studies in 32 sub-Saharan African countries spanning 1969 and 2022.*
- *We accounted for the sensitivity and specificity of different diagnostic tests used across studies and used meta-regression models to estimate the distribution of infections causing each symptom.*
- *We determined that the main aetiologies for vaginal discharge were candidiasis (69% of cases in 2015), bacterial vaginosis (50%), chlamydia (16%), and trichomoniasis (13%); for urethral discharge were gonorrhoea (77%) and chlamydia (22%); and for genital ulcer were HSV-2 (48%) and syphilis (9%).*
- *Distributions of infectious aetiologies were similar across regions and population sub-groups but changed over time.*

What do these findings mean?

- *The findings support the applicability and continued use of WHO guidelines for syndromic management across sub-Saharan African settings.*
- *National STI programmes should re-assess syndrome aetiologies regularly due to changes over time.*

3. When you submit your revised paper, please upload any figures associated with the paper as individual TIF or EPS files with 300dpi resolution at resubmission; please read our figure guidelines for more information on our requirements: http://journals.plos.org/plosmedicine/s/figures. While revising your submission, please also upload your figure files to the PACE digital diagnostic tool, https://pacev2.apexcovantage.com/. PACE helps ensure that figures meet PLOS requirements. To use PACE, you must first register as a user. Then, login and navigate to the UPLOAD tab, where you will find detailed instructions on how to use the tool. If you encounter any issues or have any questions when using PACE, please email us at PLOSMedicine@plos.org.

Response: All figures have been updated according to requirements and checked using the PACE tool.

4. When you submit your revision, please re-upload the PRISMA checklist using line numbers (rather than section alone) to indicate the relevant position of information in the paper;

Response: The PRISMA checklist with line numbers has been uploaded. We have retained a version in the supplementary material that includes section headings alone.

5. We ask every co-author listed on the manuscript to fill in a contributing author statement, making sure to declare all competing interests. If any of the co-authors have not filled in the statement, we will remind them to do so when the paper is revised. If all statements are not completed in a timely fashion this could hold up the re-review process. If new competing interests are declared later in the revision process, this may also hold up the submission. Should there be a problem getting one of your co-authors to fill in a statement we will be in contact. Please ensure that you do not add or remove authors without first discussing this and gaining agreement from the handling editor. You can see our competing interests policy here: http://journals.plos.org/plosmedicine/s/competing-interests.

Response: We have requested that all co-authors complete the statement.

6. Minor: Please place references inside punctuation (Vancouver style)

Response: All references have been updated to Vancouver style.

Academic Editor comments:

1. There is a lack of attention to heterogeneity between studies: the very informative Figure 1 indicates that there is substantial heterogeneity, but this needs to be quantified. What was the between-study variance (tau squared)? How much of the variance was explained by the variables included in the meta-regression? Which variable explained the most?

Response: Thank you for your comment. Consistent with advice, we have revised Tables S13 to report the following measures for the meta-regression models informing Figure 2: variance attributed to fixed and random effects, variance attributed to observation-level effects, variance attributed to the binomial distribution, and total variance. We have also included these measures in Tables S14 and S15.

In the method section of the main manuscript, we have added (line 216-218): "*Study observation heterogeneity is assessed per model as the percentage of total variance attributed to observation-level random effects (22).*"

In the results section, we have added (line 292-295): "*Study observations were heterogeneous for all three symptoms, especially vaginal discharge. The percentage of total variance attributed to observation-level random effects was 43.0% for VD, 22.3% for UD, and 25.0% for GU (Table S13).*"

2. The assessment of risk of bias is not appropriate. The JBI instrument does not assess the risk of bias; rather, it is a more general measure that mixes risk of bias criteria with reporting and general study quality. Several criteria used have nothing to do with the risk of bias (for example, 1, 7, 10).

Further, summary scores should be avoided: they involve inappropriate equal weighting of the different criteria. See also Dekkers et al. COSMOS-E statement. PLoS Med 2019. My advice would be to analyse the criteria related to bias (3, 4, 5, 8) separately in the meta-regression. It would also be interesting to compare the convenience sampling studies with those using consecutive or random sampling.

Response: We thank the editor for the suggestions on the risk of bias assessment. Consistent with the advice, we have revised the assessment to (i) indicate the types of measures assessed by the modified JBI appraisal tool, (ii) removed the summary score, and (iii) analyse each criterion separately in meta-regressions for each symptom.

The methods section has been updated (line 194-203): "*We adapted the Joanna Briggs Institute critical appraisal tool for prevalence studies to assess study design (objective of the study), selection bias (clarity of inclusion criteria, appropriateness of recruitment method, adequacy of participation, and detail of participant characterisation), measurement bias (objectivity in symptom definition, consistency of diagnostic methodology, and avoidance of misclassified results), precision (sufficiency of sample size), and reporting quality (ambiguity of results) (Table S8) (21). Each of the 10 criteria were independently double assessed for each report, with discrepancies resolved through consensus or by a third reviewer. We assessed the association of these criteria with the RTI proportions by extending the meta-regressions to include fixed effects for each criterion.*"

The results section has been updated (line 323-337): "*Fewer than half of studies aimed to assess the aetiology of genital symptoms (N_{VD}=37/87, N_{UD}=32/55, N_{GU}=40/80, Table S18). Most studies were at risk of selection bias; participant inclusion criteria were often clearly defined (N_{VD}=82/87, N_{UD}=50/55, N_{GU}=73/80), but few studies recruited participants using consecutive or random sampling methods (N_{VD}=32/87, N_{UD}=27/55, N_{GU}=46/80), had adequate participation rates (N_{VD}=34/87, N_{UD}=21/55, N_{GU}=40/80), or provided sufficient detail on participant characteristics to determine their representativeness (N_{VD}=36/87, N_{UD}=21/55, N_{GU}=56/80). Most studies were not susceptible to measurement bias; the majority defined participant symptoms objectively (N_{VD}=48/87, N_{UD}=36/55, N_{GU}=75/80), employed consistent diagnostic methodologies (N_{VD}=81/87, N_{UD}=47/55, N_{GU}=75/80), and avoided misclassifying infections by testing for multiple pathogens (N_{VD}=43/87, N_{UD}=22/55, N_{GU}=48/80). Studies generally had sample sizes of at least 100 participants (N_{VD}=61/87, N_{UD}=39/55, N_{GU}=46/80) and reported results unambiguously (N_{VD}=51/87, N_{UD}=37/55, N_{GU}=52/80). Several of these appraisal criteria were associated with the odds of RTI diagnosis, particularly for vaginal discharge, but the hierarchy of aetiologies per symptom remained the same (Figure S3).*"

In the supplementary material, Table S8 now classifies criteria in the appraisal tool as relating to study design, selection bias, measurement bias, precision, and reporting quality. Table S17 now includes a footnote classifying each criterion according to the above categories. Table S18 has been added to summarise the criteria across studies included for each symptoms. Figure S3 has been replaced to now shows the association of each criterion with the RTI proportions for each symptom.

3. I felt the authors tend to overinterpret their findings. For example, the data do not support the recommendation that a survey should only be done every 5 years. Rather, the authors should stress that studies are urgently needed in many countries without data. Also, the limitations of the syndromic approach should be discussed. An interesting question in this context relates to the proportion of patients who will not receive adequate treatment based on their results.

Response: We appreciate this editorial comment. We have revised the discussion accordingly, particularly highlighting the risk of inadequate treatment under the syndromic management approach, lack of data in one-third of countries, and insufficient frequency of assessments in most others (line 358-367 and line 376-388):

“We also identified a notable proportion of vaginal discharge cases attributed to chlamydia, emphasising recommendations for speculum examination to detect cervicitis in the absence of aetiologic testing (3). Furthermore, approximately 5% of vaginal discharge and urethral discharge cases were attributed to M. genitalium in 2015. Current WHO guidelines recommend assessing for M. genitalium only in instances of recurrent or persistent discharge (3), potentially leaving this infection untreated. Additional attention to MG is therefore warranted, particularly amid ongoing debates on aetiologic testing in higher-income settings (38). Moreover, over a quarter of vaginal discharge and genital ulcer cases lacked an identified cause, presenting a persistent treatment challenge, even if aetiologic testing becomes more widely available.”

“Symptom aetiologies have changed over time, particularly for genital ulcer. The leading cause of genital ulcer transitioned from chancroid to HSV-2 during 1990 and 2010… Temporal changes underscore the need for regular aetiologic assessment of syndromes. However, among the 32 with data in our review, the publication rate approximated one study every ten years (median of 5 studies per country during 1969 and 2022) and 16 countries had no data all, falling short of the WHO’s recommended assessment frequency of 2 years (6).”

Reviewer 1:

Thank you for the opportunity to review this manuscript. This is a valuable and timely study. It is delightful to see this study conducted, and so rigorously. The study is thorough, well-conducted, and conforms to the best standards in conducting systematic reviews. The article is well-written, and the presentation is lucid. The results are of global interest and inform the current intense discussion about the relevance of the syndromic approach for the management of STIs versus the etiological approach. The results directly inform regional (as well as global) guidelines.

In the spirit of enhancing the impact and value of this study, I suggested few minor revisions.

Response: We thank the reviewer for their positive feedback. We have addressed each comment separately below.

1. The definition of the proportion of cases that did not have an identified etiology was unclear to me. I assume the proportion depends on what is being tested in each study, but what is being tested can differ between studies. This makes this proportion not well-defined. Can you please clarify and discuss this point?

Response: The definition above is correct and has been modified in the methods for clarity (line 169-170): *“The proportion with “unknown aetiology” was only extracted from studies with observations for three or more RTI pathogens, regardless of the specific pathogens tested.”*

The definition has been discussed as a limitation (line 437-442): *“Finally, due to different numbers of pathogens examined across studies, we were required to subjectively define an unknown aetiology among study participants tested for three or more RTIs, irrespective of the pathogens examined or the number of potential pathogens present.”*

2. There is a challenge in applying adjustments for the diagnostic test performance. The reason is that the existing reported adjustments tend to be not representative, introducing errors (sometimes even non-real negative values) when applied. It is great that the authors presented results with and without these adjustments, and both overall agreed. I think it might still be useful to discuss limitations of these adjustments in the limitations section.

Response: We used a Bayesian approach for diagnostic test performance adjustment, in order to avoid the introduction of negative prevalence values. This has been described in the Supplementary file, Text S2. Limitations of the performance adjustments have been discussed (line 432-434): *“Other RTI proportions may also have been over- or under-estimated due to*

assigned diagnostic test sensitivity and specificity values, despite our efforts to ensure their accuracy."

3. The results for HSV-1 are interesting on their own but are not sufficiently discussed. The results also align with another relatively recent study (Harfouche M, Chemaitelly H, Abu-Raddad LJ. Herpes simplex virus type 1 epidemiology in Africa: Systematic review, meta-analyses, and meta-regressions. J Infect 2019; 79(4): 289-99.). I suggest some discussion here given the increasing relevance of this infection as an STI (though not strictly in Africa).

Response: We thank the reviewer for highlighting the study by Harfouche and colleagues. The revised discussion highlights the consistency of our findings with those from Harfouche *et al.* (line 356-358): *"Our estimates attributed 2% and 48% of genital ulcer cases to HSV-1 and HSV-2 in 2015, respectively, consistent with other systematic review findings of 1.2% and 51% in SSA during 1990 and 2015 (36,37)."*

4. Although such studies are rare, why did you include studies with participants as young as 10 years? It seems to me that a more appropriate age threshold is 15 years. You may want to justify your choice.

Response: Our primary interest was STI aetiologies among the sexually active adult population. We used a minimum age of 10 years to avoid excluding studies that included eligible participants. The methods have been updated (line 133-136): *"While our primary interest was studies among sexually active adult populations, we used a minimum age of 10 years to avoid excluding studies which enrolled both eligible sexually active adolescent and adult participants; in such studies (N=18), the majority of participants were adults."*

5. You may want to indicate in the limitations that the search for grey literature was not strictly systematic.

Response: We have added to the discussion (line 403-406): *"We extensively searched grey literature to identify all available data, particularly surveillance reports that might not appear in peer-reviewed academic literature. The grey literature search used similar criteria to our database search but was not strictly systematic."*

Reviewer 2:

This article represents a truly massive effort to use the existing literature to explore the cause of vaginal and penile discharge and genital ulcers in SSA from 1969 to 2022. The authors recognize a variety of limitations in this effort. The most important problem with the article lies in failure to discuss the reasons for some of the striking changes offered in the figures: huge increase in NGC, virtual disappearance of HD, reduction in TV and many, many more observations. For example, did untreated HIV set the stage for spread of HD, that has abated with ART? The purpose of the Discussion is to INTERPRET results, not reiterate them.

Response: We thank the reviewer for their helpful feedback. We have addressed each comment separately below.

1. The authors state: "STI surveillance using syndrome-based assessments is noncomprehensive and requires studies among symptomatic and asymptomatic populations." Did they not find articles that offered a view of unrecognized, untreated asymptomatic infection? For example guidelines call for routine screening of people living with HIV for STIs?

Response: Our review focused on assessing symptomatic infection and did not extract data on aetiologies among asymptomatic populations. We are therefore unable to comment on surveillance among asymptomatic populations and have accordingly removed this statement from the manuscript.

2. In the Methods the authors say "we searched from inception to 25 July 2022. I think by inception they mean 1969?

Response: Each of the four databases has a different date of inception. Our search included literature from the beginning of each database until the specified end date. We have revised the methods to clarify that this refers to the date of database inception (line 102-104): *"Embase (Ovid), MEDLINE (Ovid), Global Health (Ovid) and Web of Science were searched from database inception to 20 December 2023."*

3. The authors discuss bias " Most studies (NVD=47, NUD=30, NGU=33) had moderate risk of bias (Table 1, Table S17). Studies with higher risk of bias (NVD=19, NUD=11, NGU=12) were predominantly those with alternate study objectives, insufficient description of study participants and/or settings, only one pathogen assessed, and ambiguous reporting of outcomes. Estimates for the proportion diagnosed per pathogen over time were generally consistent when alternatively including studies of any risk level, or only studies with lower and/or moderate risk of bias (Figure S3)." . I am not sure they are describing bias (in the epidemiology sense) as much as limitations of the articles available and the veracity of sampling and tests employed? The idea of what they are trying to do could be stated more clearly.

Response: As discussed in response to the Academic Editors, we have revised the assessment to (i) indicate the types of measures assessed by the modified JBI appraisal tool, (ii) removed the summary score, and (iii) analyse each criterion separately in meta-regressions for each symptom.

The methods section has been updated (line 194-203): "*We adapted the Joanna Briggs Institute critical appraisal tool for prevalence studies to assess study design (objective of the study), selection bias (clarity of inclusion criteria, appropriateness of recruitment method, adequacy of participation, and detail of participant characterisation), measurement bias (objectivity in symptom definition, consistency of diagnostic methodology, and avoidance of misclassified results), precision (sufficiency of sample size), and reporting quality (ambiguity of results) (Table S8) (21). Each of the 10 criteria were independently double assessed for each report, with discrepancies resolved through consensus or by a third reviewer. We assessed the association of these criteria with the RTI proportions by extending the meta-regressions to include fixed effects for each criterion.*"

The results section has been updated (line 323-337): "*Fewer than half of studies aimed to assess the aetiology of genital symptoms (N_{VD}=37/87, N_{UD}=32/55, N_{GU}=40/80, Table S18). Most studies were at risk of selection bias; participant inclusion criteria were often clearly defined (N_{VD}=82/87, N_{UD}=50/55, N_{GU}=73/80), but few studies recruited participants using consecutive or random sampling methods (N_{VD}=32/87, N_{UD}=27/55, N_{GU}=46/80), had adequate participation rates (N_{VD}=34/87, N_{UD}=21/55, N_{GU}=40/80), or provided sufficient detail on participant characteristics to determine their representativeness (N_{VD}=36/87, N_{UD}=21/55, N_{GU}=56/80). Most studies were not susceptible to measurement bias; the majority defined participant symptoms objectively (N_{VD}=48/87, N_{UD}=36/55, N_{GU}=75/80), employed consistent diagnostic methodologies (N_{VD}=81/87, N_{UD}=47/55, N_{GU}=75/80), and avoided misclassifying infections by testing for multiple pathogens (N_{VD}=43/87, N_{UD}=22/55, N_{GU}=48/80). Studies generally had sample sizes of at least 100 participants (N_{VD}=61/87, N_{UD}=39/55, N_{GU}=46/80) and reported results unambiguously (N_{VD}=51/87, N_{UD}=37/55, N_{GU}=52/80). Several of these appraisal criteria were associated with the odds of RTI diagnosis, particularly for vaginal discharge, but the hierarchy of aetiologies per symptom remained the same (Figure S3).*"

In the supplementary material, Table S8 now classifies criteria in the appraisal tool as relating to study design, selection bias, measurement bias, precision, and reporting quality. Table S17 now includes a footnote classifying each criterion according to the above categories. Table S18 has been added to summarise the criteria across studies included for each symptoms. Figure S3 has

been replaced to now shows the association of each criterion with the RTI proportions for each symptom.

4. The conclusion of the article does not fit: The authors state "STI surveillance requires prevalence studies among both symptomatic and asymptomatic populations, particularly due to high rates of asymptomatic infection". But this article is NOT about asymptomatic infections. That is an entirely different topic. The authors need to think of what the reader might take from this work? They implore more frequent surveillance but I am not sure the data support this idea without further explanation of the changes observed and more consideration of the frequency if this effort?

Response: We appreciate the reviewer's comment and have accordingly removed this statement from the conclusion. The conclusion has been updated (line 443-449): *"In conclusion, the aetiology of three common STI-related symptoms were remarkably similar across regions in sub-Saharan Africa but have evolved over time, underscoring a changing STI transmission landscape and the need for regular re-assessment to inform syndromic management protocols. The observed aetiologic distributions in SSA were largely consistent with WHO recommended syndromic management algorithms without strong evidence of variation by country, context, or population strata, strengthening the generalisability of our findings to settings lacking data in SSA."*

5. Most important, the authors do not offer a clear opinion of syndromic management compared to diagnostic results. This descriptive effort surely is designed to better direct syndromic management which remains the mainstay of STD care. Is that the intention of the authors? On the other hand (for example) focus on treatment of GC, found so commonly in discharge, would leave the far less common MG untreated. It seems unwise to put forth this effort without an opinion about this dilemma, and the potential contribution of this report?

Response: Consistent with the reviewer's advice, the discuss has been revised to highlight the risk of inadequate treatment under the syndromic management approach and lack of attention to MG in WHO guidelines (line 358-367):

"We also identified a notable proportion of vaginal discharge cases attributed to chlamydia, emphasising recommendations for speculum examination to detect cervicitis in the absence of aetiologic testing (3). Furthermore, approximately 5% of vaginal discharge and urethral discharge cases were attributed to M. genitalium in 2015. Current WHO guidelines recommend assessing for M. genitalium only in instances of recurrent or persistent discharge (3), potentially leaving this infection untreated. Additional attention to MG is therefore warranted, particularly amid ongoing debates on aetiologic testing in higher-income settings (38). Moreover, over a quarter of vaginal discharge and genital ulcer cases lacked an identified cause, presenting a persistent treatment challenge, even if aetiologic testing becomes more widely available."

Reviewer 3 (statistics):

Firstly, I would like to commend the authors on the substantive amount of work undertaking this systematic review. There is a large number of included studies, and I can appreciate the workload behind such a task. Just to be clear, I will only be considering the methods and statistical analyses undertaken. Overall, I believe there is good methodological and scientific rigour utilised throughout the review. The analysis is, for the most part, well explained and is conducted well. I have some points below for the authors consideration.

Response: We thank the reviewer for their helpful feedback. We have addressed each comment separately below.

1. The authors have presented a lot of details regarding the meta-regression modelling. However, there appears to be a lack of information regarding the initial pairwise meta-analysis which led to this. While there are very helpful and detailed figures (e.g. Figure 1, although I think this is misnamed as the PRISMA flow diagram is also named Figure 1) show the aOR by vaginal discharge, urethral

discharge, and genital ulcer they do show that, in some cases, there may be high heterogeneity. For example, figure 1, vaginal discharge shows heterogeneity between the groups. There appears to be a lack of quantification of this heterogeneity (i.e. tau: the standard deviation between the studies). Please could the authors report this information. Additionally, it would be beneficial to observe how meta-regression addressed such heterogeneity and which factors were significantly associated with the observed heterogeneity.

Response: We thank the reviewer for their comment. As discussed in response to the Academic Editors, we have revised Tables S13 to report the following measures for the meta-regression models informing Figure 2: variance attributed to fixed and random effects, variance attributed to observation-level effects, variance attributed to the binomial distribution, and total variance. We have also included this in Tables S14 and S15.

In the method section of the main manuscript, we have added (line 216-218): "*Study observation heterogeneity is assessed per model as the percentage of total variance attributed to observation-level random effects (22).*"

In the results, we have added (line 292-295): *Study observations were heterogeneous for all three symptoms, especially vaginal discharge. The percentage of total variance attributed to observation-level random effects was 43.0% for VD, 22.3% for UD, and 25.0% for GU (Table S13).*

2. The risk of bias assessment includes an overall assessment, which is not recommended. The authors should consider removing this information. Currently, the risk of bias assessment gives equal weighting to each item and some of the items are to do with the study quality of reporting and not risk of bias per se. Therefore, the inclusion of the overall risk of bias score in an analysis would be misleading, and it would be more pertinent to group studies based on certain risk of bias questions as categorical outcome (yes, no, unclear). For example, questions 3, 4, 5, and 8. Additionally, the sampling utilised in the studies should also be considered (i.e. compare convenience sampling studies with those using consecutive or random sampling methods).

These re-analyses may change the interpretation of the results and this should be considered carefully. Currently, the results are interpreted positively and maybe too positively.

Response: We appreciate the reviewer's comment. As discussed in response to the Academic Editors, we have revised the assessment to (i) indicate the types of measures assessed by the modified JBI appraisal tool, (ii) removed the summary score, and (iii) analyse each criterion separately in meta-regressions for each symptom.

The methods section has been updated (line 194-203): "*We adapted the Joanna Briggs Institute critical appraisal tool for prevalence studies to assess study design (objective of the study), selection bias (clarity of inclusion criteria, appropriateness of recruitment method, adequacy of participation, and detail of participant characterisation), measurement bias (objectivity in symptom definition, consistency of diagnostic methodology, and avoidance of misclassified results), precision (sufficiency of sample size), and reporting quality (ambiguity of results) (Table S8) (21). Each of the 10 criteria were independently double assessed for each report, with discrepancies resolved through consensus or by a third reviewer. We assessed the association of these criteria with the RTI proportions by extending the meta-regressions to include fixed effects for each criterion.*"

The results section has been updated (line 323-337): "*Fewer than half of studies aimed to assess the aetiology of genital symptoms (N_{VD}=37/87, N_{UD}=32/55, N_{GU}=40/80, Table S18). Most studies were at risk of selection bias; participant inclusion criteria were often clearly defined (N_{VD}=82/87, N_{UD}=50/55, N_{GU}=73/80), but few studies recruited participants using consecutive or random sampling methods (N_{VD}=32/87, N_{UD}=27/55, N_{GU}=46/80), had adequate participation rates (N_{VD}=34/87, N_{UD}=21/55, N_{GU}=40/80), or provided sufficient detail on participant characteristics to determine their representativeness (N_{VD}=36/87, N_{UD}=21/55, N_{GU}=56/80). Most studies were not*

susceptible to measurement bias; the majority defined participant symptoms objectively (N_{VD}=48/87, N_{UD}=36/55, N_{GU}=75/80), employed consistent diagnostic methodologies (N_{VD}=81/87, N_{UD}=47/55, N_{GU}=75/80), and avoided misclassifying infections by testing for multiple pathogens (N_{VD}=43/87, N_{UD}=22/55, N_{GU}=48/80). Studies generally had sample sizes of at least 100 participants (N_{VD}=61/87, N_{UD}=39/55, N_{GU}=46/80) and reported results unambiguously (N_{VD}=51/87, N_{UD}=37/55, N_{GU}=52/80). Several of these appraisal criteria were associated with the odds of RTI diagnosis, particularly for vaginal discharge, but the hierarchy of aetiologies per symptom remained the same (Figure S3)."

In the supplementary material, Table S8 now classifies criteria in the appraisal tool as relating to study design, selection bias, measurement bias, precision, and reporting quality. Table S17 now includes a footnote classifying each criterion according to the above categories. Table S18 has been added to summarise the criteria across studies included for each symptoms. Figure S3 has been replaced to now shows the association of each criterion with the RTI proportions for each symptom.

3. PRISMA: Number of reports included was 198 but when adding the subgroups there are 227, please check and make clear.

Response: We have revised the manuscript to indicate more clearly that several studies assess the aetiology for multiple symptoms.

The breakdown by study is outlined in Table 1 and the results section (line 237-241): "*Overall, 206 reports were included from 190 independent studies (number of studies per symptom (N): N_{VD}=87, N_{UD}=55, N_{GU}=80) spanning 1969 to 2022 (Table 1). Of these, 166 studies focused on a single symptom, 16 studies on two symptoms, and 8 studies on all three symptoms."* The PRISMA diagram (Figure 1) has now been updated to provide a similar breakdown by report.

4. Text S2: In the supplementary text the authors state that in the case of multiple sources being available with wide variation, a mean sensitivity and specificity value was calculated. I believe taking the mean would be inadequate, as the variation most likely occurs due to sample size differences and other confounding factors (e.g. high vs low risk population). It would therefore be beneficial for these values to be created using a weighted mean based on the sample size.

Response: Consistent with this advice, we have revised the sensitivity and specificity values in Table S7. Where multiple sources were used to determine the sensitivity and specificity of treponemal and non-treponemal tests, the weighted mean has now been used. Supplementary Text S2 has been revised to clarify this (page 7): *"In cases where multiple sources were available with wide variation, we calculated a weighted mean for the sensitivity and specificity."*

5. The tables need to make clear that they are referring the number of studies and not number of participants.

Response: A footnote *"N = number of studies"* has been added to Tables 1, S10, S11, S12 and S18.

6. There is a lack of information regarding how the adjusted odds ratio were calculated and what factors were used. Please elaborate. A similar supplementary text as to the other analyses would be beneficial.

Response: The following explanation has been added to the manuscript (line 215-216): *"Model coefficients are presented as adjusted odds ratios (aORs), with confidence intervals calculated on the log odds scale before exponentiation."*

The methods section describes the variables included in the model (line 181-186): *"We estimated time trends in the diagnosed proportion by region via generalised linear mixed-effects meta-regressions for each symptom (19). Models were specified a priori to include fixed effects for RTI,*

the interaction of RTI and year (midpoint date of data collection measured as continuous calendar year), and the interaction of RTI and sex (genital ulcer only), random intercepts and slopes per year for the interaction of RTI and region (central and western, eastern, or southern Africa), and observation-level random intercepts to account for between-study heterogeneity."

Reviewer 4:

This manuscript by Michalow et al. describes a systematic review and meta-regression to characterise aetiologies for vaginal discharge, urethral discharge, and genital ulcer in sub-Saharan Africa. International and national guidelines for STIs across Africa are often informed by sporadic studies and surveillance activities, making amalgamation of this information very helpful. The manuscript itself is clear and comprehensive, appears methodologically sound, and is overall a very impressive piece of work providing very important data, with clear policy implications. I have a few minor comments and suggestions, largely to improve clarity.

Response: We thank the reviewer for their positive feedback. We have addressed each comment separately below.

1. Abstract: It is noted in the methods that results are reported as predictions for the year 2015 as this represents "the most recent quinquennium within the timespan of substantial available data". This makes sense - however, reading the abstract without this explanation is a bit confusing, particularly the sentence "In 2015, primary aetiologies for vaginal discharge were…" I wonder if possible to make this more clear, such that readers won't think that the results are from the year 2015? E.g. "In 2015, predicted primary aetiologies…" or providing a brief explanatory sentence if word count allows.

Response: Consistent with this suggestion, the abstract now states (line 32): "In 2015, *estimated* primary aetiologies..."

Methods:

2. UN M49 standard - The classification of the UN M49 standard is included in the supplementary material. However, I wonder if it might be possible to include this information in the main manuscript, so that readers are not required to review the supplementary material to find out this important information. This is perhaps additionally important as countries such as Zambia and Zimbabwe are often considered to be in "Southern Africa" by various other classifications, and I had actually made this assumption before reviewing the supplementary material.

Response: We have added a footnote to Table 1 in the main manuscript specifying the regional classification: *"[2] Regions classified according to UN M49 Standard. Central Africa: Angola, Cameroon, Central African Republic, Chad, Congo, Democratic Republic of Congo, Equatorial Guinea, Gabon, Sao Tome and Principe. Eastern Africa: Burundi, Comoros, Djibouti, Eritrea, Ethiopia, Kenya, Madagascar, Malawi, Mauritius, Mozambique, Rwanda, Seychelles, Somalia, South Sudan, Uganda, United Republic of Tanzania, Zambia, Zimbabwe. Southern Africa: Botswana, Eswatini, Lesotho, Namibia, South Africa. Western Africa: Benin, Burkina Faso, Cabo Verde, Cote d'Ivoire, Gambia, Ghana, Guinea-Bissau, Guinea, Liberia, Mali, Mauritania, Niger, Nigeria, Senegal, Sierra Leone, Togo."*

3. Lines 104 - 106: "Studies were included if: (1) participants were symptomatic at the time of testing, defined by the presence of either self-reported or clinician-evaluated abnormal vaginal discharge, urethral discharge, or genital ulcer" - I think it would be helpful for clarity to state that studies were included if "some" participants were symptomatic at the time of testing (rather than all participants in a study having symptoms). It is apparent from reviewing the included papers, but would be helpful to state explicitly.

Response: The inclusion criteria have been updated accordingly (line 129): *"Studies were included if: 1) there were participants symptomatic at the time of testing, defined by the presence*

of either self-reported or clinician-evaluated abnormal vaginal discharge, urethral discharge, or genital ulcer…"

4. Was there any particular rationale for a cut-off sample size of 10?

Response: The sample size limit aims to strike a balance between excluding excessively imprecise studies (those with fewer than 10 participants) while also incorporating small studies (larger than 10 but, for example, below 100 participants) and accounting for their inherent limitations. The approach ensures findings are synthesized from a diverse range of studies. A minimum sample size of 10 is a common convention in systematic reviews, as in the example references below:

- Chan GJ, Lee AC, Baqui AH, Tan J, Black RE. Risk of early-onset neonatal infection with maternal infection or colonization: a global systematic review and meta-analysis. PLoS medicine. 2013 Aug 20;10(8):e1001502.
- Mountain E, Mishra S, Vickerman P, Pickles M, Gilks C, Boily MC. Antiretroviral therapy uptake, attrition, adherence and outcomes among HIV-infected female sex workers: a systematic review and meta-analysis. PloS one. 2014 Sep 29;9(9):e105645.
- Harfouche M, Abu-Hijleh FM, James C, Looker KJ, Abu-Raddad LJ. Epidemiology of herpes simplex virus type 2 in sub-Saharan Africa: Systematic review, meta-analyses, and meta-regressions. EClinicalMedicine. 2021 May 1;35.

5. Lines 107 - 108: "diagnostic methodology for each infection was described and assessed as valid according to published recommendations." Perhaps a bit more information on how this assessment was made would be helpful.

Response: The inclusion criteria have been updated accordingly (line 136-137): *"Diagnostic test validity was assessed by cross-referencing its inclusion in either current or previous published recommendations (3,10–15)."*

6. Were fixed and random effects variables chosen a priori for models?

Response: Yes, the model terms were specified were chosen *a priori* to address the primary research question about regional levels and time trends in STI aetiologies, allowing for study heterogeneity (via observation-level random effects).

We have revised the description of the model to clarify this (line 181-186): *"Models were <u>specified a priori</u> to include fixed effects for RTI, the interaction of RTI and year (midpoint date of data collection measured as continuous calendar year), and the interaction of RTI and sex (genital ulcer only), random intercepts and slopes per year for the interaction of RTI and region (central and western, eastern, or southern Africa), and observation-level random intercepts to account for between-study heterogeneity."*

Results:

7. Lines 207-209: "Studies were predominantly in South Africa (NVD=12/83, NUD=15/53, NGU=17/78), and Nigeria (NVD=15/83) for vaginal discharge and Kenya (NGU=18/78) for genital ulcer." Was there supposed to be a reference to urethral discharge in this statement? Referring to South Africa?

Response: For urethral discharge, most studies were in South Africa, and the remaining studies were evenly distributed across countries. The statement has been updated for clarity (line 243-244): *"Studies were predominantly in South Africa (N_{VD}=13/87, N_{UD}=10/55, N_{GU}=18/80), <u>along with</u> Nigeria (N_{VD}=15/87) for vaginal discharge and Kenya (N_{GU}=18/80) for genital ulcer."*

8. The four included figures in the main manuscript are numbered as follows: figure 1, figure 2, figure 1, figure 2. Re-numbering is required.

Response: We thank the reviewer for noting this error, it has been corrected.

Discussion:

9. Lines 304-305: "The distribution of aetiologies estimated for each symptom were consistent with WHO syndromic management algorithms." As MG was diagnosed in 5.6% of urethral discharge cases and 5.4% of vaginal discharge cases, but is not included in either management guideline, I wonder if the authors would like to briefly comment on the merits of the inclusion of MG in such algorithms. This may be particularly apt given the finding that "exceptions included higher diagnosed proportions of M. genitalium than chlamydia among younger women with vaginal discharge and HIV-positive men with urethral discharge." I am aware of the global debate around MG testing, so even a comment simply acknowledging this may be appropriate.

Response: Consistent with this advice, we have highlighted the lack of attention to MG in WHO guidelines (line 358-367):

"We also identified a notable proportion of vaginal discharge cases attributed to chlamydia, emphasising recommendations for speculum examination to detect cervicitis in the absence of aetiologic testing (3). Furthermore, approximately 5% of vaginal discharge and urethral discharge cases were attributed to M. genitalium in 2015. Current WHO guidelines recommend assessing for M. genitalium only in instances of recurrent or persistent discharge (3), potentially leaving this infection untreated. Additional attention to MG is therefore warranted, particularly amid ongoing debates on aetiologic testing in higher-income settings (38). Moreover, over a quarter of vaginal discharge and genital ulcer cases lacked an identified cause, presenting a persistent treatment challenge, even if aetiologic testing becomes more widely available."

Reviewer 5:

This systematic review and characterisation of the aetiologies of the three key STI syndromes (vaginal discharge, urethral discharge and genital ulcers) in sub-saharan Africa is very timely and useful and extremely thoroughly and competently conducted, and the findings are very well and clearly written. The authors should be congratulated for an excellent job. The main text, figures and tables are very clear and concise. The supplementary material is very detailed and useful. The conclusions broadly support the current recommendations of WHO: 1) that the current syndromes management guidelines cover well the main aetiologies under each syndrome; 2) that there is a need to regularly appraise the etiological composition of syndrome as these do change over time, particularly genital ulcer syndrome, and may also vary by HIV status (again more so for genital ulcer) and slightly by age (more chlamydia and gonorrhoea I'm younger ages). Interestingly, the estimates of etiological composition do not vary much by geographical region, and the estimated distribution have remained relatively constant even despite the change of diagnostic procedures, with the introduction of NAAT tests more recently. Overall, this work supports the robustness of WHO recommendations for syndromic management, which is reassuring. The studies tend to confirm the near disappearance of some aetiologies such as Haemophilus ducreyi (chancroid) and LGV among ulcers, and T vaginalis among female and male discharges. This is quite likely the result of nearly 30 years of implementation of syndromic management, and it would be interesting to show if this was supported by prevalence studies in general populations (among asymptomatic).

Response: We thank the reviewer for their positive feedback. We have addressed each comment separately below.

There are no major comments. But a few points:

1. This reviewer could not verify all the publications that were included in the review and possibly some that may have been omitted, although data appear to have been thoroughly checked.

2. One surprising finding is the high proportion of candidiasis found as aetiology of VD (and particularly coexisting with BV and /or TV, given the vaginal pH enabling the yield of CS vs TV/BV goes in opposite directions).

Response: We agree with the reviewer's comment. Estimates for CS are also sensitive to the diagnostic test performance adjustments, although the performance values for these tests were specified using the best available evidence. We have noted this as a potential limitation (line 429-432): *"In contrast, the diagnosed proportion for CA was 45% relative to CS, which was below the expected range of 70-90% (51,52). Adjustments to the performance of gram stain and/or wet mount may have overestimated CS proportions, while CA may have been underestimated due to the limited number of observations."*

3. One aetiology for GU is surprisingly missing -- donovanosis, which used to be diagnosed in South Africa in the 1990s (eg O'Farrell) -- this would not very much change the etiological profile other than signalling its disappearance.

Response: We used the WHO 2021 guideline for symptomatic STI management to inform the selection of aetiologies included in our study. While donovanosis is briefly mentioned in the guidelines in reference to men with persistent anogenital ulcers, it has not been included in the flow chart for genital ulcer management nor the list of treatment options available for genital ulcer aetiologies, and hence was not considered in this study.

4. Regarding syphilis serology in GU, what rule did the authors (or the primary study authors) use to attribute a syphilis aetiological results, for example in the absence of demonstrable TP pathogen in the lesion?

Response: When studies used both ulcer swab tests and serology tests, our priority was to extract results from the swab test, even when no identifiable cases of the pathogen were reported. For studies that did not conduct ulcer swab tests, we extracted serologic results as available. This was outlined in the methods (line 165-167): *"When multiple diagnostic tests were used for syphilis among those with genital ulcer, we preferentially extracted observations for tests using ulcer swab specimens over serology (3)."*

A few minor comments or clarifications:

5. There is a slight discrepancy between the number of study reports mentioned in text (top of p9 and Table 1, ie NVD=83, NUD=53 NGU=78, total 183, but should not this be 214?), when figure 1 indicates 88, 53 and 86 respectively (total 198, should be 227). I would guess this is because some studies covered more than one syndrome? Perhaps that proportion should be given in text.

Response: We have revised the manuscript to indicate more clearly that several studies assess the aetiology for multiple symptoms.

The breakdown by study is outlined in Table 1 and the results section (line 237-241): "*Overall, 206 reports were included from 190 independent studies (number of studies per symptom (N): N_{VD}=87, N_{UD}=55, N_{GU}=80) spanning 1969 to 2022 (Table 1). Of these, 166 studies focused on a single symptom, 16 studies on two symptoms, and 8 studies on all three symptoms."* The PRISMA diagram (Figure 1) has now been updated to provide a similar breakdown by report.

6. It is not clear how the data from the two identified databases (NICD and CESAHHR) were aggregated to the rest of the data. The CESAHHR data needed some transformation (weighting) to account for study design (RDS survey); what about the NICD data? Were the results obtained simply added to the other data?

Response: The following has been added to the supplementary file, Text S1. Firstly, to reflect that NICD data were unweighted: *"We extracted simple (unweighted) diagnosed proportions, stratified*

by year, sex, HIV-status, and age group.” Secondly, to indicate that CeSHHAR and NICD data tabulations were merged with the other data: “*The data extracted from each database, following the approaches outlined below, were combined with published study data before analysis.”*

7. It is not clear why the year 2015 is chosen to give the results?

Response: We have described the rationale for year 2015 in the methods section (line 212-214): “*Pooled results are reported as meta-regression model predictions for the year 2015, representing the most recent quinquennium within the timespan of substantial available data.”*

8. Whilst inclusion criteria insisted on studies among symptomatic patients, there appears in several places that such studies accounted for 85% or so of patients? why?

Response: We extracted aetiologic outcomes for symptomatic study participants, although the study may also have included asymptomatic participants. The inclusion criteria have accordingly been updated for clarity (line 129): *“Studies were included if: 1) there were participants symptomatic at the time of testing, defined by the presence of either self-reported or clinician-evaluated abnormal vaginal discharge, urethral discharge, or genital ulcer…”*

9. Top of p15/line 358 -- 'our focus on discharge symptoms...' suggest this section starts as new paragraphs -- it seems to be dealing with limitations of the analyses. By the way the sentence discharge symptoms rather than syndromes is not clear.... is it because the authors may have included studies that reported patients with discharge, or dysuria etc rather than strictly defined as 'syndrome' -- and did they (or the authors) treat the aetiologies attached to each symptoms as equally contributing to the 'syndrome'?

Response: This topic falls within the penultimate ‘limitations’ paragraph of the manuscript. We have clarified our focus on discharge symptoms rather than syndromes (line 422-425): *“We specifically focused on discharge symptoms, rather than broader syndrome definitions, to accommodate varied reporting across studies and over time. However, this more inclusive definition may have influenced estimated aetiologic distributions.”*

10. The numbering of figures in main text goes off -- Fig 1, 2, are repeated twice

Response: We thank the reviewer for noting this error, it has been corrected.

11. Supplementary Table S5 = under CT diagnosis 'antibody test' done on genital fluid and/or urine: is that correct? is it not rather 'antigen detection' assay?

Response: We thank the reviewer for noting this error, it has been corrected to “ELISA”.

12. Supplementary S7 -- for syphilis - sensitivity of dark field of 80%?? this seems rather high -- it depends on performer!

Response: We used WHO documentation for the sensitivity and specificity values. The range reported for darkfield sensitivity has been fairly consistent over time: 74-86% (WHO 1999) and 75-100% (WHO 2023). While the performer’s influence on the accuracy of darkfield is well noted, in the absence of better information to quantify performance in different contexts, we have retained the WHO reported ranges for our primary analysis.

In the limitations section, we note the uncertainty about and heterogeneity in performance characteristics for many of the diagnostics (line 432-434): *“Other RTI proportions may also have been over- or under-estimated due to assigned diagnostic test sensitivity and specificity values, despite our efforts to ensure their accuracy.”*

- World Health Organization. Laboratory tests for the detection of reproductive tract infections. 1999.

- World Health Organization. Laboratory and point-of-care diagnostic testing for sexually transmitted infections, including HIV. 2023.

Reviewer 6:

This systematic review was conducted to determine whether the aetiologies of the three main STI syndromes (vaginal discharge, urethral discharge and genital ulcer) in sub-Saharan Africa (SSA) would be treated by the management algorithms recommended by WHO.

The findings are interesting and useful; however, some revisions are required.

Abstract:

1. Conclusion needs to be amended. Considering that only studies of STI syndromes (i.e. involving symptomatic patients) were analysed, the findings do not speak to the conclusion that STI surveillance using syndrome-based assessments is non-comprehensive and requires studies in asymptomatic populations. Although this statement is correct, this conclusion cannot be reached by the findings of this meta-analysis.

> **Response:** We appreciate the reviewer's comment and have accordingly removed this statement from the conclusion. The conclusion has been updated (line 42-44): *"Syndrome aetiologies in SSA align with WHO guidelines without strong evidence of geographic or demographic variation, supporting broad guideline applicability. Temporal changes underscore the need for regular aetiologic re-assessment."*

2. Would also state that aetiologic reassessment needs to be periodic or regular.

> **Response:** The has been updated in the abstract (line 44): *"Temporal changes underscore the need for regular aetiologic re-assessment."*

Introduction:

3. It is important for the authors to clarify why a symptom is used to define a particular syndrome throughout the manuscript, when STIs are generally managed as syndromes (defined by groups of symptoms) in regions having limited access to laboratory diagnostics. Aetiological studies usually investigate the causative organisms of STI syndromes.

> **Response:** We have clarified our focus on discharge symptoms rather than syndromes in our discussion of limitations (line 422-425): *"We specifically focused on discharge symptoms, rather than broader syndrome definitions, to accommodate varied reporting across studies and over time. However, this more inclusive definition may have influenced estimated aetiologic distributions."*

4. Lines 67 - 68: this statement regarding WHO needs to be substantiated. When considering the diagnostic performance of treatment algorithms and flowcharts, the WHO guideline development group reviewed the relative prevalence of aetiologies per syndrome in order to determine the predictive value of treatment algorithms (refs 25 - 27).

> **Response:** We acknowledge and agree that prevalence data were reviewed to inform development of the guidelines, however aetiologic distributions were not quantified and geographic differences were not explicitly presented. We have updated the statement to reflect this (line 90-92): *"Although the guidelines accounted for changes over time in the underlying causes of each syndrome, they lacked a comprehensive review and quantification of the STI distribution among symptomatic populations in different geographic areas."*

Methods:

5. Lines 106 - 107: how was a sample size of 10 considered appropriate for inclusion? RTI estimates likely to be very imprecise. The risk of bias assessment tool (Table S8) states that the

sample size should be justified (cannot be done for small sample sizes e.g. 10, 20) or the sample should include at least 100 participants. The authors must state how the inclusion of smaller studies was justified.

<u>Response:</u> The sample size limit aims to strike a balance between excluding excessively imprecise studies (those with fewer than 10 participants) while also incorporating small studies (larger than 10 but, for example, below 100 participants) and accounting for their inherent limitations. The approach ensures findings are synthesized from a diverse range of studies. A minimum sample size of 10 is a common convention in systematic reviews, as in the example references below:

- Chan GJ, Lee AC, Baqui AH, Tan J, Black RE. Risk of early-onset neonatal infection with maternal infection or colonization: a global systematic review and meta-analysis. PLoS medicine. 2013 Aug 20;10(8):e1001502.
- Mountain E, Mishra S, Vickerman P, Pickles M, Gilks C, Boily MC. Antiretroviral therapy uptake, attrition, adherence and outcomes among HIV-infected female sex workers: a systematic review and meta-analysis. PloS one. 2014 Sep 29;9(9):e105645.
- Harfouche M, Abu-Hijleh FM, James C, Looker KJ, Abu-Raddad LJ. Epidemiology of herpes simplex virus type 2 in sub-Saharan Africa: Systematic review, meta-analyses, and meta-regressions. EClinicalMedicine. 2021 May 1;35.

6. Line 121: authors should define key populations considered in analysis.

<u>Response:</u> The definition of population types has been updated (line 149-152): *"…population type (symptomatic clinic attendees; general populations; higher-risk general populations, such as truck drivers, mineworkers, soldiers, and bar workers; or key populations, such as sex workers, and men who have sex with men)..."*

7. Lines 140 - 141: the authors state that unknown aetiology for RTIs was extracted only from studies that tested for >=3 infectious pathogens. Does this refer to STI pathogens or even to RTIs such as BV and vulvovaginal candidiasis? This is important in the context of vaginal discharge syndrome where the aetiology is more diverse and investigation for only 3 infectious pathogens/RTIs may be insufficient to define an "unknown aetiology" category.

<u>Response:</u> We have modified the definition in the methods to clarify that we are referring to all RTIs, and not specifically STIs (line 169-170): *"The proportion with "unknown aetiology" was only extracted from studies with observations for three or more RTI pathogens, regardless of the specific pathogens tested."*

The definition has been discussed as a limitation (line 437-442): *"Finally, due to different numbers of pathogens examined across studies, we were required to subjectively define an unknown aetiology among study participants tested for three or more RTIs, irrespective of the pathogens examined or the number of potential pathogens present."*

Data Analysis:

8. Line 152 - 153: why were fixed effects models used for RTI estimation instead of random effects models? Considering the heterogeneity of methodologies used in different studies (so that the between-study variance is taken into account). This needs clarification.

<u>Response:</u> We used mixed-effects models, incorporating both fixed effects to quantify systematic trends and regional variation and random effects to account for between-study heterogeneity, as highlighted by the reviewer.

We have clarified the use of random effects account for between-study variation in the methods section (line 180-186): *"We estimated time trends in the diagnosed proportion by region <u>via generalised linear mixed-effects meta-regressions</u> separately for each symptom (19). Models were*

specified a priori to include fixed effects for RTI, the interaction of RTI and year (midpoint date of data collection measured as continuous calendar year), and the interaction of RTI and sex (genital ulcer only), random intercepts and slopes per year for the interaction of RTI and region (central and western, eastern, or southern Africa), and observation-level random intercepts *<u>to account for between-study heterogeneity.”</u>*

Results:

9. Lines 225 - 227: It is not clear whether the proportion of VD with Candida species (CS) could include those with C. albicans (CA).

<u>Response:</u> CS is defined as *“any Candida species* (line 119)”. This encompasses all species within the Candida genus, including Candida albicans.

10. Lines 225 - 231: the authors state that CS and BV are the most common VD aetiologies; however it would be important to document the proportion of RTI-STI co-infections, as aetiological studies have found that a significant proportion of these RTIs are co-infected with STI pathogens; therefore syndromic treatment of VD is complex and suboptimal (over- and under-treatment).

<u>Response:</u> Although co-infections are common among individuals with VD, it was not within the scope of our analysis to quantify the proportion with these co-infections. We have added this to the limitations (line 434-437): *“Our modelling approach and estimates did not account for relationships between similar outcomes, such as the contribution of HSV-1 and HSV-2 to unspecified HSV; <u>co-infection rates between RTIs</u>; or the negative correlation between proportions for the same symptom, which depend on co-infection rates.”*

Risk of Bias assessment:

11. Most studies used non-probability (convenience) sampling - this introduces selection bias. Was this considered?

<u>Response:</u> Yes, criteria 4 of appraisal tool assesses the method used for participant recruitment (Table S8). It assesses whether studies recruited participants appropriately, defined as using either consecutive or random sampling methods.

The results section has been updated (line 324-329): “*Most studies were at risk of selection bias; participant inclusion criteria were often clearly defined (N_{VD}=82/87, N_{UD}=50/55, N_{GU}=73/80), but <u>few studies recruited participants using consecutive or random sampling methods (N_{VD}=32/87, N_{UD}=27/55, N_{GU}=46/80),</u> had adequate participation rates (N_{VD}=34/87, N_{UD}=21/55, N_{GU}=40/80), or provided sufficient detail on participant characteristics to determine their representativeness (N_{VD}=36/87, N_{UD}=21/55, N_{GU}=56/80).”*

Discussion:

12. Lines 305 - 309: The authors should reconsider the statement that vaginal discharge management should prioritise candidiasis, BV and trichomoniasis, considering the proportion of mixed RTI-STI in aetiological studies. This differs from WHO VDS management algorithm, which emphasizes testing for STI pathogens, where possible; and where unavailable, treating for N. gonorrhoeae and C. trachomatis in high-risk women or those with visible abnormal discharge on genital exam.

<u>Response:</u> We appreciate the reviewer’s comment. The WHO guideline identifies candidiasis, BV and trichomoniasis as the main causes of VDS (page 45). The VDS algorithm recommends the following, in contexts where rapid point of care tests are unavailable (page 52, Option D): 1) treat for N. gonorrhoeae and C. trachomatis based on evidence of cervicitis on speculum examination, 2) treat for bacterial vaginosis and T. vaginalis if vaginal discharge present or based on results of

microscopy, and 3) treat for candidiasis according to type of discharge or if confirmed by microscopy. We have updated the discussion point (line 353-361): *"Aligned with guidelines, vaginal discharge management should prioritise candidiasis, bacterial vaginosis, and trichomoniasis* <u>*as primary aetiologies...*</u>*. We also identified a notable proportion of vaginal discharge cases attributed to chlamydia, emphasising recommendations for speculum examination to detect cervicitis* <u>*in the absence of aetiologic testing*</u> *(3)."*

13. Lines 306 - 309: speculum examination, although recommended in the absence of STI diagnostic tests, has poor sensitivity for diagnosing cervical infections (Flowchart 2, reference 25).

<u>Response:</u> We agree that the vaginal discharge flowchart has poor sensitivity for cervical infection diagnosis. However, including a speculum examination in the flowchart increases the sensitivity for cervicitis diagnosis from 28% (history and risk assessment) to 45% (history, risk assessment, and speculum examination), and is recommended when aetiologic testing is not available. We updated the statement to (line 358-361): "*We also identified a notable proportion of vaginal discharge cases attributed to chlamydia, emphasising recommendations for speculum examination to detect cervicitis* <u>*in the absence of aetiologic testing*</u> *(3)."*

14. Lines 316 - 321 (prevalence of pathogens by HIV status and age): these statements are not borne out by the Results (Lines 262 - 275).

<u>Response:</u> The section has been revised to align with the updated analysis (line 370-375): *"HIV-status and age group were significantly associated with the diagnosis of several RTIs, but the overall hierarchy of aetiologies for each symptom largely remained unchanged. The main exception was for HIV-positive women with vaginal discharge, where diagnosed proportions were higher for bacterial vaginosis than candidiasis, and trichomoniasis and gonorrhoea than chlamydia. However, this would not alter implementation of the management algorithm for vaginal discharge syndrome."*

15. Lines 330 - 332: while regional aetiological data may be used to inform some national syndromic management protocols, it would be important to monitor national antimicrobial resistance trends for key pathogens, where possible as this may differ in certain areas.

<u>Response:</u> We have revised this sentence to highlight the importance of monitoring antimicrobial resistance for key pathogens (line 385-388): *"In the absence of local studies, our findings suggest data from neighbouring regions or countries can be used to inform syndromic management protocols, although* <u>*national-level monitoring of antimicrobial resistance for key pathogens may still be necessary*</u> *(41).*

16. Lines 336 - 338: would also be important to emphasize the contribution of the HIV epidemic to the changing epidemiology of genital ulcer disease.

<u>Response:</u> We have revised the discussion to include (line 379-381): "*The HIV epidemic has also influenced the epidemiology of ulcerative infections over time, with documented synergy between HSV-2 and HIV."*

17. Lines 337 - 338: there is some evidence that the relative proportion of primary syphilis as a cause of genital ulcer disease may be increasing in the region (aetiological surveillance data, South Africa). This may partly be attributable to national stock-outs of benzathine penicillin G.

<u>Response:</u> We have incorporated the aetiologic surveillance data from South Africa in our analysis (NICD database) and did not identify an increase over time in the proportion of genital ulcer cases due to syphilis. This result also holds when analysing by region in SSA.

18. Line 340: not sure why trichomoniasis is included as a cause of genital ulceration.

<u>Response:</u> The reference to trichomoniasis was not linked to GUD. To avoid confusion, we have updated the statement (line 394-396): *"Decreasing aetiologic proportions for syphilis and chancroid were consistent with general population prevalence trends in SSA (16,42–45)."*

19. Line 346: to determine overall STI burden, it is necessary to determine prevalence among asymptomatic persons in the general population (representativeness and sample size important).

<u>Response:</u> We have updated that statement to reflect that data are required on STI prevalence among a representative sample of the general population. This would include both asymptomatic and symptomatic individuals (line 399-422). *"Using our results to estimate overall STI burdens requires additional data on the prevalence of STI syndromes, care seeking, and aetiologies, collected from <u>representative samples</u> of the general population."*

20. Lines 356 - 366 - only a relatively small number of studies cultured and speciated Candida isolates or did Germ tube test?

<u>Response:</u> Yes, that is correct and included as a discussion point (line 431-432): *"…CA may have been underestimated due to the limited number of observations".*

21. Limitation: studies with small sample sizes included; more likely to have positive results - subject to publication bias.

<u>Response:</u> The sample size limit aims to strike a balance between excluding excessively imprecise studies (those with fewer than 10 participants) while also incorporating small studies (larger than 10 but, for example, below 100 participants) and accounting for their inherent limitations. The approach ensures findings are synthesized from a diverse range of studies. A minimum sample size of 10 is a common convention in systematic reviews, as in the example references below:

- Chan GJ, Lee AC, Baqui AH, Tan J, Black RE. Risk of early-onset neonatal infection with maternal infection or colonization: a global systematic review and meta-analysis. PLoS medicine. 2013 Aug 20;10(8):e1001502.
- Mountain E, Mishra S, Vickerman P, Pickles M, Gilks C, Boily MC. Antiretroviral therapy uptake, attrition, adherence and outcomes among HIV-infected female sex workers: a systematic review and meta-analysis. PloS one. 2014 Sep 29;9(9):e105645.
- Harfouche M, Abu-Hijleh FM, James C, Looker KJ, Abu-Raddad LJ. Epidemiology of herpes simplex virus type 2 in sub-Saharan Africa: Systematic review, meta-analyses, and meta-regressions. EClinicalMedicine. 2021 May 1;35.

Additional points:

22. The investigators accessed raw data from two large databases in South Africa and Zimbabwe. It would be important for the investigators to document the local permissions and ethics approvals obtained for accessing these, in addition to the approval obtained for the study protocol from the IRB at Imperial College, London.

<u>Response:</u> Local IRB approval was not required for the use of these two datasets. Sharing of anonymised surveillance data for secondary analysis in meta-analysis did not meet the definition of human subjects research under South African and Zimbabwe regulations, and therefore did not require local IRB approval. Institutional data sharing agreements were established governing that only fully anonymised data were shared and the specified use of data.

- South African National Department of Health. South African Ethics in Health Research Guidelines: Principles, Processes and Structures. 3rd Edition. 2023.
- Medical Research Council of Zimbabwe. Ethics Guidelines for Health Research Involving Human Participants in Zimbabwe. Version 1.4. 2011

23. The investigators have stated that where these national databases were available, prior full text publications of these data were excluded from the meta-analysis. It would be important to give reasons for the exclusion. Prior publications from South Africa have highlighted the relatively high prevalence of RTI-STI co-infection in vaginal discharge syndrome (for which syndromic management is complex and not optimal); as well as a relative increase in the prevalence of primary syphilis as a cause of genital ulcer disease. These are not highlighted in the manuscript and are points that I raised in my review.

Response: We chose to use the two databases for outcome tabulation as it allowed the inclusion of unpublished surveillance rounds (NICD) and enabled additional stratification of outcomes (NICD and CeSHHAR). We excluded published manuscripts as they contain the same data and would result in duplication. We have updated the methods (160-161): *"When available, we used observations tabulated directly from databases and excluded corresponding published articles to avoid duplication."*

We did not include the assessment of co-infections in our analysis and have acknowledged this as a limitation (line 434-437): *"Our modelling approach and estimates did not account for relationships between similar outcomes, such as the contribution of HSV-1 and HSV-2 to unspecified HSV; co-infection rates between RTIs; or the negative correlation between proportions for the same symptom, which depend on co-infection rates."*
