# Supplementary material for: Aetiology of vaginal discharge, urethral discharge, and genital ulcer in sub-Saharan Africa: A systematic review and meta-regression
Source: PLoS Med. 2024 May 20;21(5):e1004385. doi: 10.1371/journal.pmed.1004385 (PMC11104670; doi:10.1371/journal.pmed.1004385)
Supplement: Attachment — Submitted filename: JMichalow_Response to reviewers.pdf [file pmed.1004385.s005.pdf]

Response to reviewers

General comments:

Throughout the paper, please adapt reference call-outs to the following style: "... every year [1,2]." (noting the absence of spaces within the square brackets).

Response: The reference style has been updated throughout the manuscript.

Please use person-first language throughout your manuscript e.g. ‘men with HIV’ rather than “HIV-positive men”, including in figure legends

Response: The results section has been revised as requested:

“For vaginal discharge, the odds were higher among women with HIV than women without HIV for diagnosis with MG… (line 320)*”*

“For urethral discharge, the odds of TV diagnosis were higher (aOR: 1.9 [95% CI: 1.1-3.3], nUD=35) among men with HIV than men without HIV… (line 329)*”*

“For genital ulcer, the odds of unspecified HSV (aOR: 1.8 [95% CI: 1.1-2.8], nGU=53) and HSV-2 (aOR: 1.6 [95% CI: 1.1-2.5], nGU=57) were higher among participants with HIV than those without HIV… (line 334)*”*

The legend for Figure 3 has been revised:

Estimated diagnosed proportion per pathogen among adults with and without HIV in 2015 (left) and adjusted odds of diagnosis per pathogen among adults with HIV compared to adults without HIV (right) symptomatic with (A) vaginal discharge, (B) urethral discharge, and (C) genital ulcer… Points represent the adjusted odds of diagnosis among adults with HIV…”

When referring to age, please revise to e.g. “The odds were higher among women <25 years than ≥25 years for diagnosis…”

Response: The results section of the manuscript has been revised to:

“*The odds were higher among women <25 years than ≥25 years for diagnosis…* (line 324)”

“*CT was the only RTI associated with age for urethral discharge, with higher odds among men <25 years…* (line 331)”

“…*had higher odds of diagnosis among participants <25 years* (line 338)”

To help us extend the reach of your research, please provide any Twitter handle(s) that would be appropriate to tag, including your own, your coauthors’, your institution, funder, or lab.

Response: Please see a list of relevant twitter handles below:

@julmichalow

@mkwalters3

@LanreEdun

@SungaiChabata

@dr_anne_cori

@eatonjw

@MRC_Outbreak

@nicd_sa

@CeSHHAR

@ImperialSPH

@CCDD_HSPH

@HarvardEpi

@HarvardChanSPH

Title: Please revise your title to "...Saharan Africa: A systematic review and meta-regression"

Response: The title has been revised in the manuscript and supplementary material.

Abstract:

Please ensure that your abstract is reported according to PRISMA for abstracts, following the PLOS Medicine abstract structure (Background, Methods and Findings, Conclusions): http://www.plosmedicine.org/article/info:doi/10.1371/journal.pmed.1001419

Response: The abstract has been revised to: (1) state the review objective clearly in the Introduction, (2) indicate that studies were critically appraised in Methods and Findings, and (3) include limitations of the review in the Methods and Findings.

*"**Introduction**: Syndromic management is widely used to treat symptomatic sexually transmitted infections in settings without aetiologic diagnostics. However, underlying aetiologies and consequent treatment suitability are uncertain without regular assessment. This systematic review estimated the distribution, trends, and determinants of aetiologies for vaginal discharge, urethral discharge, and genital ulcer in sub-Saharan Africa (SSA).*

***Methods and Findings**: We searched Embase, MEDLINE, Global Health, Web of Science, and grey literature from inception until December 20, 2023, for studies reporting aetiologic diagnoses among symptomatic populations in SSA. We adjusted observations for diagnostic test performance, used generalised linear mixed-effects meta-regressions to generate estimates, and critically appraised studies with an adapted checklist. Of 4418 identified records, 206 reports were included from 190 studies in 32 countries conducted between 1969 and 2022. In 2015, estimated primary aetiologies for vaginal discharge were candidiasis (69.4% [95% confidence interval (CI): 44.3-86.6%], n=50), bacterial vaginosis (50.0% [95%CI: 32.3-67.8%], n=39), chlamydia (16.2% [95%CI: 8.6-28.5%], n=50), and trichomoniasis (12.9% [95%CI :7.7-20.7%], n=80); for urethral discharge were gonorrhoea (77.1% [95%CI: 68.1-84.1%%], n=68) and chlamydia (21.9% [95%CI: 15.4-30.3%], n=48); and for genital ulcer were herpes simplex virus type 2 (HSV-2) (48.3% [95%CI: 32.9-64.1%], n=47) and syphilis (9.3% [95%CI: 6.4-13.4%], n=117). Temporal variation was substantial, particularly for genital ulcer where HSV-2 replaced chancroid as the primary cause. Aetiologic distributions for each symptom were largely the same across regions and population strata, despite HIV-status and age being significantly associated with several infection diagnoses. Limitations of the review include the absence of studies in 16 of 48 SSA countries, substantial heterogeneity in study*

observations, and impeded assessment of this variability due to incomplete or inconsistent reporting across studies.

***Conclusion**: In our study, syndrome aetiologies in SSA aligned with World Health Organization guidelines without strong evidence of geographic or demographic variation, supporting broad guideline applicability. Temporal changes underscore the importance of regular aetiologic re-assessment for effective syndromic management."*

Please provide further detail on the dates of search (from inception), types of study designs included, eligibility criteria, and synthesis/appraisal methods.

Response: The methods section of the abstract has been revised:

"We searched Embase, MEDLINE, Global Health, Web of Science, and grey literature from inception until December 20, 2023… (line 28)"

Please define CI at first use.

Response: The results section of the abstract has been revised:

"*In 2015, estimated primary aetiologies for vaginal discharge were candidiasis (69.4% [95% confidence interval (CI): 44.3-86.6%], n=50)…* (line 34)"

Please add "95% CI" to each of the square brackets for clarity.

Response: The results section of the abstract has been revised to incorporate 95% CI in each square bracket:

"In 2015, estimated primary aetiologies for vaginal discharge were candidiasis (69.4% [95% confidence interval (CI): 44.3-86.6%], n=50), bacterial vaginosis (50.0% [95%CI: 32.3-67.8%], n=39), chlamydia (16.2% [95%CI: 8.6-28.5%], n=50), and trichomoniasis (12.9% [95%CI: 7.7-20.7%], n=80); for urethral discharge were gonorrhoea (77.1% [95%CI: 68.1-84.1%%], n=68) and chlamydia (21.9% [95%CI: 15.4-30.3%], n=48); and for genital ulcer were herpes simplex virus type 2 (HSV-2) (48.3% [95%CI: 32.9-64.1%], n=47) and syphilis (9.3% [95%CI: 6.4-13.4%], n=117)."

Please define HSV at first use.

Response: Herpes simplex virus type 2 (HSV-2) has been defined in the results section of the abstract (line 38).

In the last sentence of the Abstract Methods and Findings section, please describe 2-3 of the main limitations of the study's methodology.

Response: The limitations have been added to the abstract:

"Limitations of the review include the absence of studies in 16 of 48 SSA countries, substantial heterogeneity in study observations, and impeded assessment of this variability due to incomplete or inconsistent reporting across studies (line 42-45)*."*

Please begin your Abstract Conclusions with "In this study, we observed ..." or similar, to summarize the main findings from your study, without overstating your conclusions. Please emphasize what is new and address the implications of your study, being careful to avoid assertions of primacy.

Response: The conclusion of the abstract has been revised to:

"<u>In our study</u>, syndrome aetiologies in SSA aligned with World Health Organization guidelines without strong evidence of geographic or demographic variation, supporting broad guideline applicability. Temporal changes underscore the importance of regular aetiologic re-assessment for effective syndromic management (line 46-49)*."*

Please define WHO at first use.

<u>Response</u>: The full term "World Health Organization" has been provided in the conclusions section of the manuscript (line 47).

Author Summary:
Thank you for providing an Author Summary.
Lines 48 & 68 - please revise 'Syndromic case management' for clarity for a broad readership.

<u>Response</u>: The author summary has been updated:

"<u>Symptom-based case management</u> is common for treating sexually transmitted infections (STIs) in sub-Saharan Africa (line 54)*."*

"The findings support the applicability and continued use of WHO guidelines for <u>symptomatic STI management</u> across sub-Saharan African settings (line 73)*."*

Line 69 - please define STI at first use.

<u>Response</u>: STI has been defined at first use on line 55 in the author summary.

In the final bullet point of 'What Do These Findings Mean?', please describe the main limitations of the study in non-technical language.

<u>Response</u>: The following has been added as a final bullet:

"Limitations of the review include than no data were available in 16 of 48 sub-Saharan African countries, there was large variability in the results across studies identified in the review, and certain information of interest was missing or inconsistently reported across studies (line 77-80)*."*

Methods:
Please ensure to identify any changes in the analysis (including those made in response to peer review comments) in the Methods section of the paper, with rationale.

Please add the following statement, or similar, to the Methods: "This study is reported as per the Preferred Reporting Items for Systematic Reviews and Meta-Analyses (PRISMA) guideline (S1 Checklist)." Two copies of the PRISMA checklist have been provided - please only include the version currently included as Table S9. This should be a standalone file relabelled "S1 checklist"

<u>Response</u>: The methods section includes the following statement:

"This systematic review and meta-analysis was pre-registered on PROSPERO (CRD: 42022348045) (25) and reported as per the Preferred Reporting Items for Systematic Reviews and Meta-Analyses (PRISMA) guideline (S1 Checklist) (26) (line 235-238)*"*

Table S9 has been moved to S1 Checklist. The tables in S1 Appendix and referenced in the S1 Checklist have therefore been re-numbered. The manuscript

has been updated to reference tables in S1 Appendix with the updated table numbers.

Results:
Line 258 onwards: Please add "95% CI" to each of the brackets for clarity.

Response: The results section has been updated to include 95% CI in brackets for all reported estimates.

Line 266 - please define aOR at first use.

Response: The definition is included in the methods section:

"Model coefficients are presented as adjusted odds ratios (aORs)… (line 228)."

Line 306 - please revise to "The odds were higher among women <25 years than ≥25 years for diagnosis…"

Response: This has been updated:

"*The odds were higher among women <25 years than ≥25 years for diagnosis…* (line 324)"

Discussion:
Line 349 - Should this be "1970 to 2023"?

Response: No, the studies included in our analysis were conducted during 1969 and 2022. Our estimates therefore did not extend beyond 2022.

Please present and organize the Discussion as follows: a short, clear summary of the article's findings; what the study adds to existing research and where and why the results may differ from previous research; strengths and limitations of the study; implications and next steps for research, clinical practice, and/or public policy; one-paragraph conclusion.

Response: The discussion has been restructured as follows: concise summary of key findings, comparison to existing research and implications for policy, limitations in interpreting the aetiologic proportions, limitations of the analysis, conclusion.

[Concise summary of key findings] *"This systematic review and meta-regression estimated the distribution, trends, and determinants of aetiologies for STI-related symptoms in sub-Saharan Africa from 1970 to 2022. The primary aetiologies identified for vaginal discharge were candidiasis, bacterial vaginosis, and trichomoniasis, for urethral discharge were gonorrhoea and chlamydia, and for genital ulcer were herpes and syphilis. Additionally, over 5% of vaginal discharge and urethral discharge cases were attributed to M. genitalium, and over a quarter of vaginal discharge and genital ulcer cases lacked an identified cause in 2015. Symptom aetiologies changed over time, particularly for genital ulcer where the leading cause shifted from chancroid in 1990 to HSV-2 by 2010. We did not find evidence of systematic regional variation in the aetiologic distributions. Although HIV status and age group were significantly associated with the diagnosis of several RTIs, the overall hierarchy of aetiologies for each symptom largely remained unchanged..*

[Comparison to existing research and implications for policy] *"The estimated distribution of aetiologies for each symptom was consistent with infections prioritised by WHO syndromic management guidelines [3]. Our analysis adds to previous reviews [31–35] that informed WHO syndromic algorithms by incorporating a wider range of*

studies reporting underlying syndrome aetiologies, specifically in SSA. We, however, did not find evidence for needing to adapt the algorithms for SSA settings or populations. Guidelines currently recommend assessing for M. genitalium only in instances of recurrent or persistent discharge [3], which would potentially leave this infection untreated. Additional attention to MG is therefore warranted, particularly amid ongoing debates on aetiologic testing in higher-income settings [36]. The high proportion of cases without an identifiable cause poses a persistent unaddressed treatment challenge, even with expanded access to diagnostic testing.

Changes over time in genital ulcer aetiology are consistent with existing evidence indicating the near eradication of chancroid [37]. The reduction in chancroid cases is largely attributed to the introduction of antibiotics for genital ulcer management. The prevalence of HSV-2 has also declined, only much more slowly [38]. Furthermore, the HIV epidemic has also shaped the epidemiology of ulcerative infections over time, with documented synergy between HSV-2 and HIV [39]. We identified that 2% and 48% of genital ulcer cases were attributed to HSV-1 and HSV-2 in 2015, respectively, consistent with other systematic review findings of 1.2% and 51% in SSA during 1990 and 2015 [38,40].

These temporal changes underscore the need for regular aetiologic assessment of syndromes. However, among 32 countries with data in our review, the publication rate approximated one study every ten years (median of 5 studies per country during 1969 and 2022), falling short of the WHO's recommended assessment frequency of 2 years [6]. In the absence of local studies, our findings suggest data from neighbouring regions or countries can be used to inform syndromic management protocols, although national-level monitoring of antimicrobial resistance for key pathogens may still be necessary [41].

[Limitations in interpreting the aetiologic proportions] "*Aetiologic proportions among symptomatic populations are a component of characterising RTI burden, but the two are not equivalent. Diagnosed proportions reflect the prevalence of all possible aetiologies for a particular symptom, conditional on individuals being symptomatic and seeking care. For example, while HSV-2 prevalence has decreased in the region, the proportion of genital ulcer cases attributed to HSV-2 increased over time because the prevalence of chancroid and syphilis declined more rapidly [38,42–44]. Decreasing aetiologic proportions for syphilis and chancroid were consistent with general population prevalence trends in SSA [16,42–45]. Diagnosed proportions also do not reflect the relative RTI burden among different population groups. Although aetiologic proportions were distributed similarly by HIV status, age group, and sex (genital ulcer) for each symptom, the prevalence of RTIs in SSA is higher among women and people living with HIV, and varies by age [46,47]. Using our results to estimate overall STI burdens requires additional data on the prevalence of STI syndromes, care seeking, and aetiologies, collected from representative samples of the general population.*

[Limitations of the analysis] "*Our analysis had limitations, mainly related to features of the data and studies. We extensively searched grey literature to identify all available data, particularly surveillance reports that might not appear in peer-reviewed academic literature. The grey literature search used similar criteria to our database search but was not strictly systematic. We did not find any studies in 16 countries, particularly in central Africa (5/9 countries had no studies). However, the consistency of results across countries with data provides some confidence that key syndromic management recommendations are generalisable to countries that did not have any or recent*

studies. Most studies were not nationally representative and were conducted among a convenience sample of symptomatic individuals seeking healthcare at specific facilities. Our analysis did not account for factors influencing treatment access, such as urban or rural location [48–50], or symptom severity, recurrence, or treatment history, which could have influenced the aetiologic distributions among study participants but for which data were not consistently available. Additionally, as most studies did not report HIV prevalence or age among symptomatic participants, we were unable to adjust for these factors in the overall estimates. These factors may have contributed to heterogeneity in the study observations, which was particularly large for vaginal discharge. Our definition of vaginal or urethral discharge based on abnormal symptoms may have captured a subset of those meeting criteria for diagnosis with vaginal discharge syndrome (abnormal discharge, vulval irritation, and/or itching) or urethral discharge syndrome (abnormal discharge, dysuria, and/or itching). Alternatively, our symptom-based definition may have included more individuals with non-infectious aetiologies than a clinical syndromic diagnosis. The symptom-based definition was chosen to accommodate variations in reporting across studies and over time, but may have influenced estimated aetiologic distributions. Several studies did not differentiate between HSV type or Candida species; we considered these data representative of HSV-2 and CA trends, which may have overestimated their contribution. Our estimated diagnosed proportion for HSV-2 was 85% that of unspecified HSV, which was consistent with previous studies [38]. In contrast, the diagnosed proportion for CA was 45% relative to CS, which was below the expected range of 70-90% [51,52]. Adjustments to the performance of gram stain and/or wet mount may have overestimated CS proportions, while CA may have been underestimated due to the limited number of observations. Other RTI proportions may also have been over- or under-estimated due to assigned diagnostic test sensitivity and specificity values, despite our efforts to ensure their accuracy. Our modelling approach and estimates did not account for relationships between similar outcomes, such as the contribution of HSV-1 and HSV-2 to unspecified HSV; co-infection rates between RTIs; or the negative correlation between proportions for the same symptom, which depend on co-infection rates. Finally, due to different numbers of pathogens examined across studies, we were required to subjectively define an unknown aetiology among study participants tested for three or more RTIs, irrespective of the pathogens examined or the number of potential pathogens present. Despite these limitations, to the best of our knowledge, our study provides the most comprehensive analysis to date on the aetiology of STI-related symptoms in SSA.

[Conclusion] *"In conclusion, the aetiology of three common STI-related symptoms were remarkably similar across regions in sub-Saharan Africa but have evolved over time, underscoring a changing STI transmission landscape and the need for regular re-assessment to inform syndromic management protocols. The observed aetiologic distributions in SSA were largely consistent with WHO recommended syndromic management algorithms without strong evidence of variation by country, context, or population strata, strengthening the generalisability of our findings to settings lacking data in SSA."*

Line 368 - please revise to '...no evidence for…'

Response: This has been updated to *"We, however, did not find evidence for.."* (line 383).

Line 441 - please temper assertions of primacy by adding ‘to the best of our knowledge’ or similar

Response: This statement has been updated as:

“Despite these limitations, to the best of our knowledge, our study provides the most comprehensive analysis to date on the aetiology of STI-related symptoms in SSA (line 456-458).*”*

Please remove the information on competing interests, funding and data sharing from the end of the main text. This information will appear in the article metadata, via entries in the submission form.

Response: This has been removed.

Figures:
Fig 3, 4 - Please indicate which factors are adjusted for in the figure legend

Response: The following has been added to each figure legend:

Figure 2: *“Proportions estimated using generalised linear mixed-effects model for each symptom with fixed effects for RTI and the interaction of RTI with year and sex (genital ulcer only), random intercepts and slopes per year for the interaction of RTI and region, and observation-level random intercepts.”*

Figure 3: *“Proportions and odds estimated using generalised linear mixed-effects model for each symptom with fixed effects for RTI and the interaction of RTI with year, HIV status, and sex (genital ulcer only), random intercepts for the interaction of RTI and region, and observation-level random intercepts.”*

Figure 4: *“Proportions and odds estimated using generalised linear mixed-effects model for each symptom with fixed effects for RTI and the interaction of RTI with year, age group, and sex (genital ulcer only), random intercepts for the interaction of RTI and region, and observation-level random intercepts.”*

Please define abbreviations used in the figure legend of each figure, including in the supplementary information.

Response: All figure legend abbreviations have been checked and updated in the supplementary file. Abbreviations for the main figures have already been defined.

Please consider avoiding the use of red and green in order to make your figure more accessible to those with colour blindness.

Response: All figures have been reviewed using a colour-blind simulator (https://pilestone.co.uk/pages/color-blindness-simulator), to ensure accessible interpretation.

Tables:
Please define abbreviations used in the table legend of each table, including in the supplementary information.

Response: All table legend abbreviations have been checked and updated in the supplementary file. Abbreviations for the main table have already been defined.

Supplementary tables - Where aOR are presented, please indicate which factors are adjusted for in the respective table legend

Response: The following has been added to the legends for relevant tables in the supplementary material:

Tables S12: *"Odds estimated using generalised linear mixed-effects model for each symptom with fixed effects for RTI and the interaction of RTI with year and sex (genital ulcer only), random intercepts and slopes per year for the interaction of RTI and region, and observation-level random intercepts."*

Table S13: *"Odds estimated using generalised linear mixed-effects model for each symptom with fixed effects for RTI and the interaction of RTI with year, HIV-status, and sex (genital ulcer only), random intercepts for the interaction of RTI and region, and observation-level random intercepts."*

Table S14: *"Odds estimated using generalised linear mixed-effects model for each symptom with fixed effects for RTI and the interaction of RTI with year, age group, and sex (genital ulcer only), random intercepts for the interaction of RTI and region, and observation-level random intercepts."*

When a p value is given, please specify the statistical test used to determine it. When reporting p values please report as p<0.001 and where higher as the exact p value e.g. p=0.002.

Response: We have revised our reporting of results by removing p-values from Figure S3 and Tables S12, S13 and S14 in the supplementary material. No p-values were referred to in the main manuscript.

Please provide the unadjusted comparisons as well as the adjusted comparisons.

Response: Table S18 has been added to the supplementary appendix (page 30):

Table S18: Estimated diagnosed proportion per pathogen in sub-Saharan Africa, with and without diagnostic test performance adjustment.

RTI	Adjusted proportion % (95% CI)		Unadjusted proportion % (95% CI)	
	1980	2015	1980	2015
**A. Vaginal discharge**				
CS	5.6 (1.5-18.2)	69.4 (44.3-86.6)	24.7 (14.5-38.9)	36.2 (23.9-50.6)
BV	45.5 (14.7-80.1)	50.0 (32.3-67.8)	24.7 (10.4-48.1)	37.6 (25.6-51.3)
CA	26.8 (7.6-62.1)	31.5 (12.4-59.9)	27.1 (12.8-48.6)	33.0 (16.4-55.3)
CT	5.3 (1.7-15.0)	16.2 (8.6-28.5)	4.0 (1.9-8.6)	13.1 (8.0-20.7)
TV	51.6 (32.3-70.5)	12.9 (7.8-20.7)	24.1 (15.2-36.1)	9.7 (6.5-14.2)
NG	10.6 (4.9-21.7)	6.6 (3.3-12.7)	7.0 (3.6-13.2)	5.9 (3.3-10.3)
MG	6.4 (0.2-65.2)	5.4 (1.8-14.8)	9.8 (1.0-53.4)	7.0 (3.1-15.4)
None	25.6 (6.5-63.1)	25.1 (10.7-48.4)	29.8 (12.9-54.8)	22.9 (11.9-39.5)
**B. Urethral discharge**				
NG	63.9 (47.1-77.8)	77.1 (68.1-84.1)	54.5 (40.9-67.5)	70.3 (61.6-77.7)
CT	10.6 (4.4-23.2)	21.9 (15.4-30.3)	8.3 (4.0-16.3)	19.1 (13.9-25.7)
MG	23.5 (7.0-55.4)	5.5 (3.7-8.3)	22.7 (8.3-49.1)	5.0 (3.4-7.4)
TV	29.3 (15.6-48.0)	2.8 (1.8-4.3)	12.5 (6.9-21.5)	3.9 (2.6-5.6)
None	38.9 (13.6-72.0)	10.0 (6.8-14.5)	39 (16.3-67.7)	9.5 (6.6-13.5)
**C. Genital ulcer**				
HSV	7.8 (4.3-13.7)	57.1 (40.9-71.9)	5.0 (2.6-9.5)	58.7 (41.1-74.4)
HSV-2	39.8 (19.4-64.4)	48.3 (32.9-64.1)	36.6 (16.1-63.4)	49.5 (32.6-66.5)
TP	17.9 (12.4-25.1)	9.3 (6.4-13.4)	16.2 (10.8-23.6)	7.8 (5.1-11.7)
LGV	24.6 (7.7-55.9)	2.3 (1.0-5.3)	47.4 (16.2-80.7)	1.2 (0.5-3.2)

HSV-1	1.8 (0.3-10.8)	2.0 (0.8-4.7)	2.6 (0.3-21.5)	0.6 (0.2-2.2)
HD	86.9 (79.7-91.8)	2.0 (1.2-3.3)	81.8 (71.8-88.9)	0.9 (0.5-1.7)
None	20.5 (12.9-30.9)	34.7 (25.5-45.1)	19.9 (12.0-31.2)	33.9 (24.2-45.3)

References:
Please ensure that journal name abbreviations match those found in the National Center for Biotechnology Information (NCBI) databases (http://www.ncbi.nlm.nih.gov/nlmcatalog/journals), and are appropriately formatted and capitalised. Please also see https://journals.plos.org/plosmedicine/s/submission-guidelines#loc-references for further details on reference formatting.

> **Response**: All references have been checked. Capitalisation, formatting, and journal name abbreviations have been amended as required.

Comments from Reviewers:

Reviewer #1: Thank you for considering and addressing my comments.

Reviewer #3: I appreciate the hard work that has gone into updating this review. The authors have been very accommodating to the reviewers and made substantial changes that have improved the manuscript.

Reviewer #5: Most comments I had made have been answered appropriately. Two minute crrections/additions:

I would simply nuance the statement made by the authors re use of Darkfield microscopy for TP. I could not find the statement of high sensitivity of nearly 100% in the WHO STI Laboratory manual 2023 -- at best the authors of the chapter on Syphilis stated that DF should be reserved to specialist laboratories and experienced readers. only there can sensitivity be so high. So add - ....in experienced microscopists somewhere.

> **Response**: The following statement has been added to the supplementary file, Text S2 (page 7):
>
> *"Diagnostic test performance varies based on factors such as disease stage, sample handling, and performer competency. For example, WHO advises that darkfield microscopy is used in specialised laboratories by experienced microscopists. However, we lacked data to evaluate such influencing factors."*

They somehow did not state what they considered syphilis positive when no pathogen were recovered but syphilis serology results were available -- did they go with the interpretation made by the original authors, or did they define themselves active syphilis, and if so how? (this should be mentioned in the paper)

> **Response**: We included the below explanation in the manuscript on the use of ulcer swab and serology tests:
>
> *"When multiple diagnostic tests were used for syphilis among those with genital ulcer, we preferentially extracted observations for tests using ulcer swab specimens over serology* (line 177)*."*
>
> Therefore, if ulcer swab diagnostic tests were negative, we recorded no syphilis aetiology and did not revert to using serology results.

The authors have replaced antibody test for CT by ELISA. I still assume they talk about antibody or antigen detection system, eg direct immunofluorescent assay (DFA) or Ag detection test, again as described in the relevant chapter of the WHO Lab manual 2023 -- surely no one is making a diagnosis of CT by serological ELISA.

Response: We classified the diagnostic tests according to each study's methodology. For chlamydia, the test categories include direct immunofluorescence assays (DFA), enzyme-linked immunosorbent assays (ELISA), and antigen-based rapid point-of-care assays (WHO laboratory manual, page 120) for assessment of vaginal, cervical, or urethral swab samples. We excluded studies with diagnostic methods that were assessed as invalid, including studies reporting use of serological ELISA (n = 1).
