# Supplementary material for: Aetiology of vaginal discharge, urethral discharge, and genital ulcer in sub-Saharan Africa: A systematic review and meta-regression
Source: PLoS Med. 2024 May 20;21(5):e1004385. doi: 10.1371/journal.pmed.1004385 (PMC11104670; doi:10.1371/journal.pmed.1004385)
Supplement: Attachment — Submitted filename: JMichalow_Response to reviewers.docx [file pmed.1004385.s006.docx]

**Response to reviewers**

Thank you for sharing the code that underpins the findings in your manuscript. Because Github depositions can be readily changed or deleted, please make a permanent DOI copy (e.g. in Zenodo) and provide this URL for this in your Data Availability Statement.

**Response**: The data availability statement has been updated to:

*Data extracted from included studies and used for analysis are available as supplementary material (S2 Appendix). Code reproducing the analysis is available from https://doi.org/10.5281/zenodo.10849676.*

In your Abstract, please provide further detail on the types of study designs included (e.g. cohort studies, etc), and methods for assessing risk of bias. Please ensure that all numbers presented are identical to numbers presented in the main manuscript text.

**Response**: The Methods and Findings have been updated (line 27-33):

*“We searched Embase, MEDLINE, Global Health, Web of Science, and grey literature from inception until December 20, 2023, for observational studies reporting aetiologic diagnoses among symptomatic populations in SSA. We adjusted observations for diagnostic test performance, used generalised linear mixed-effects meta-regressions to generate estimates, and critically appraised studies using an adapted Joanna Briggs Institute checklist.”*

All numbers in the abstract have been verified as identical to the numbers in the main manuscript.

Throughout the Results section, please indicate clearly where the full results are shown (table or figure numbers) for the results presented in the main text. Please ensure that all numbers presented in main text are identical to numbers presented in the tables.

**Response**: Figure and table references have been checked throughout the main text. All numbers presented in the main text have been verified as identical to the numbers presented in the tables.

Line 256 - Where is it shown that "studies were predominantly in South Africa"?

**Response**: This section of the results has been updated to (line 254-258):

*“Most studies were in eastern Africa (NVD=35/87, NUD=32/55, NGU=50/80) and few were in central Africa (NVD=4/87, NUD=2/55, NGU=2/80). Studies were predominantly in South Africa (NVD=13/87, NUD=10/55, NGU=18/80), along with Nigeria (NVD=15/87) for vaginal discharge and Kenya (NGU=18/80) for genital ulcer (S1 Appendix Figure S1).”*

Line 265: For results relating to sample size of 100 or more, NGU differs between the main text and Table 1. Please clarify.

**Response**: Thank you for noting this error. The main text contained the correct number, and Table 1 has accordingly been updated to 46 (57.5%).

Please incorporate Table S15 into the main paper.

**Response**: Tables S15 has been changed to Table 2. The tables in the supplementary appendix have therefore been renumbered. Table references have been updated in the main text.

Please refer also to Table S12C in the paragraph relating to genital ulcers.

**Response**: Table S12C has been referred to in the main text (line 295).

Line 301 - Where is it shown that "In 1980, estimates were highest for HD"?

**Response**: The sentence has been updated (line 300):

*“In 1980, estimates were highest for HD (86.9% [95% CI: 79.7-91.8%]) and lowest for HSV (7.8% [95% CI: 4.3-13.7%]; Figure 2C, S1 Appendix Table S17C).”*

Line 340 - Where is it shown that "men with genital ulcer had lower odds (aOR: 0.4 [95% CI: 0.3-0.7]; aOR: 0.6 [95% CI: 0.4-1.0]) of 341 diagnosis with HSV-2 than women"?

**Response**: The sentence has been updated (line 339):

*“In both analyses, men with genital ulcer had lower odds (aOR: 0.4 [95% CI: 0.3-0.7]; aOR: 0.6 [95% CI: 0.4-1.0]) of diagnosis with HSV-2 than women (S1 Appendix Tables S13C and S14C).”*
